# Dynamic and Kinetic Elements of µ-Opioid Receptor Functional Selectivity

**DOI:** 10.1038/s41598-017-11483-8

**Published:** 2017-09-12

**Authors:** Abhijeet Kapoor, Gerard Martinez-Rosell, Davide Provasi, Gianni de Fabritiis, Marta Filizola

**Affiliations:** 1 Department of Pharmacological Sciences, Icahn School of Medicine at Mount Sinai, New York, NY USA; 2 Computational Biophysics Laboratory (GRIB-IMIM), Universitat Pompeu Fabra, Barcelona Biomedical Research Park (PRBB), C Dr Aiguader 88, Barcelona, 08003 Spain; 3 Institució Catalana de Recerca i Estudis Avançats (ICREA), Passeig Lluis Companys 23, Barcelona, 08010 Spain

## Abstract

While the therapeutic effect of opioids analgesics is mainly attributed to µ-opioid receptor (MOR) activation leading to G protein signaling, their side effects have mostly been linked to β-arrestin signaling. To shed light on the dynamic and kinetic elements underlying MOR functional selectivity, we carried out close to half millisecond high-throughput molecular dynamics simulations of MOR bound to a classical opioid drug (morphine) or a potent G protein-biased agonist (TRV-130). Statistical analyses of Markov state models built using this large simulation dataset combined with information theory enabled, for the first time: a) Identification of four distinct metastable regions along the activation pathway, b) Kinetic evidence of a different dynamic behavior of the receptor bound to a classical or G protein-biased opioid agonist, c) Identification of kinetically distinct conformational states to be used for the rational design of functionally selective ligands that may eventually be developed into improved drugs; d) Characterization of multiple activation/deactivation pathways of MOR, and e) Suggestion from calculated transition timescales that MOR conformational changes are not the rate-limiting step in receptor activation.

## Introduction

Opioid therapeutics that target the main (orthosteric) µ-opioid receptor (MOR) binding site remain the preferred treatment for chronic pain, which is known to affect more individuals than those impacted by cancer, heart disease, and diabetes combined^1^. However, these classical opioid drugs (e.g., morphine) produce a number of dangerous side effects (e.g., respiratory depression), which have captured the public’s attention due to an increased number of opioid overdose deaths in the last decade^2–4^.

Like other members of the family of G protein-coupled receptors (GPCRs), MOR undergoes ligand-specific conformational changes upon binding, leading to the activation of G protein and/or β-arrestin signaling pathways. Notably, suppression of morphine’s analgesic efficacy in MOR knockout mice suggested that this receptor is absolutely necessary to mediate morphine action on pain pathways^5^. While mice lacking β-arrestin2 exhibited enhanced morphine analgesia suggesting that the drug’s beneficial effect is mediated by G proteins, MOR-dependent β-arrestin recruitment appeared to contribute to some of the side effects of classical opioids^6–8^.

Although the majority of known opioid analgesics activate both G protein and β-arrestin pathways, a few MOR ligands have recently been shown to have an improved pharmacological profile *in vivo* by virtue of their G protein-biased agonism. Among them is TRV-130, a potent analgesic exhibiting less respiratory depression and constipation than morphine^9, 10^, which is currently being evaluated in human clinical trials for acute pain management^11–13^.

Comparison between the high-resolution crystal structures of inactive^14^ and activated MOR^15^ bound to the morphinans β-funaltrexamine (β-FNA) and BU72, respectively, suggests very small structural differences in the extracellular region of the receptor with larger conformational changes occurring at its cytoplasmic side as the result of a large outward movement of transmembrane helix (TM) TM6 relative to TM3 and smaller inward movements of TM5 and TM7. Notably, the classical R^3.50^-D/E^6.30^ salt bridge (superscript numbers refer to the Ballesteros and Weinstein’s generic numbering scheme^16^) that stabilizes the inactive conformation of TM6 in a number of inactive GPCR crystal structures (e.g., refs 17, 18 and 19–21) does not form in MOR because of the lack of an acidic amino acid at position 6.30. This salt bridge is replaced by a hydrogen bond between R165^3.50^ and T279^6.34^ in the MOR inactive crystal structure and a hydrogen bond between R165^3.50^ and Y252^5.58^ in the MOR activated crystal structure. Together with residues N332^7.49^, Y336^7.53^, L158^3.43^, and V285^6.40^, Y252^5.58^ is also involved in a hydrogen bonding network that stabilizes the inward movement of the so-called N^7.49^PxxY^7.53^ motif towards TM5 in the MOR activated crystal structure.

To shed light on the dynamics between the inactive and activated crystallographic states of MOR, solution-state NMR was recently used to obtain spectra of the unliganded receptor, as well as MOR bound to the agonist BU72, and MOR bound to both BU72 and a G protein-mimetic nanobody^22^. These studies suggested a weak allosteric coupling between the agonist binding site and the TM5 and TM6 regions of MOR at the G protein-coupling interface. Notably, intracellular loop 1 (ICL1) and helix 8 (H8) showed larger spectral changes compared to changes in TM5 and TM6 in the presence of the agonist alone, suggesting that TM5 and TM6 motions intervene later in complex formation.

To obtain an atomic-level resolution of the ligand-specific transition between inactive and activated structures of MOR, including detailed information about the metastable conformations along the activation pathways and the system’s kinetic properties, we carried out molecular dynamics (MD) simulations of morphine-bound and TRV-130-bound MOR systems. Specifically, to increase exploration of under-sampled regions of the system’s conformational space that are inaccessible to standard MD simulations, we applied the high-throughput molecular dynamics (HTMD)^23^ adaptive sampling protocol^24^. This protocol allows one to carry out thousands of simulations in sequential batches, starting new simulations from under-sampled regions of the system’s conformational space identified using Markov State Models (MSMs) obtained from the simulations of previous batches. Comparison of the results of information theory and other state-of-the-art statistical analyses of the MSMs constructed from these simulations led to identifying specific structural, thermodynamic, and kinetic differences at the basis of MOR functional selectivity.

## Results

### Adaptive sampling simulations allow thorough explorations of ligand-specific conformational transitions between the activated and inactive crystal structures of MOR

We generated microsecond-scale trajectories for morphine-bound (~240 µs) or TRV-130-bound (~220 µs) activated MOR, in the absence of G protein mimetics and embedded in a hydrated 1-palmitoyl-2-oleoyl-sn-glycero-3-phosphocholine (POPC)/10% cholesterol bilayer, using an adaptive sampling protocol (see Methods for details). Projection of the trajectory frames onto the root mean square deviation (RMSD) from the active or inactive crystal structures of MOR shows that the reported simulations successfully explore the conformational transition between these states, in the presence of either ligand (Supplementary Results, Supplementary Fig. 1a and d for the morphine-bound and TRV-130-bound receptor, respectively). Notably, the distribution of the sidechain and backbone atom distances between residues R165^3.50^ and T279^6.34^ (Supplementary Fig. 1b and e for the morphine-bound and TRV-130-bound receptor, respectively), which are found to form a hydrogen bond in the inactive, but not the activated, crystal structures of MOR, show that the simulations starting from the activated crystal structure did sample the inactive crystallographic state of MOR. However, similar to the results of other MD simulations of MOR recently reported in the literature (e.g., see refs 25–27 or 28 for a recent review), these simulations also sampled an alternative, inactive conformation of the agonist-bound MOR, in which the hydrogen bond between the side chains of R165^3.50^ and T279^6.34^ was found to be broken while the salt bridge between D164^3.49^ and R165^3.50^ remained formed. Notably, equilibrium probabilities obtained from the simulations reported herein (see Methods) revealed that ~96% of the inactive receptor conformations sampled by either ligand-bound MOR systems contained a broken R165^3.50^-T279^6.34^ hydrogen bond. Moreover, the R165^3.50^-Y252^5.58^ hydrogen bond that has been suggested to stabilize the inward movement of TM5 in the activated crystallographic state of MOR, is broken in both these identified inactive states of MOR with either morphine or TRV-130 bound at the orthosteric site (Supplementary Fig. 1c and f, respectively).

### Markov State Modeling reveals a differential modulation of the receptor conformational landscape by morphine and TRV-130

To ascertain the most important motions driving MOR activation in the presence of different ligands, we first carried out time-independent component analyses (tICA) of the slowest conformational degrees of freedom accessible to either morphine-bound or TRV-130-bound MOR, including descriptors of protein conformation and protein-ligand interactions (see details in Methods). The significantly different tICA results obtained for the two ligand-receptor complexes prevented us from applying a common definition of microstates and macrostates for the two systems. Supplementary Tables 1–10 list the top 75 (1%) structural descriptors of the slowest conformational degrees of freedom accessible to morphine-bound and TRV-130-bound MOR identified by dominant tICA dimensions 1–10. The reduced conformational space defined by these 10 dominant tICA coordinates for either morphine-bound or TRV-130-bound MOR was discretized into 1000 microstates, and a series of MSMs was built for increasing lag-times (see Methods). Supplementary Fig. 2a,b show convergence of the dynamics of the morphine-bound and TRV-130-bound MOR systems for time resolutions equal or longer than 10 ns. Kinetic analyses reported thereafter use an MSM constructed at this lag time, which estimates the system’s slowest relaxation timescale to be ~5 µs for the morphine-bound MOR, and ~9 µs for the TRV-130-bound receptor (see blue line in Supplementary Fig. 2a and b). The aforementioned 1000 microstates were grouped into 14 macrostates (metastable states) based on their kinetic similarity (see Methods).

The consistency between the dynamics described by the MSM and the actual MD trajectories was verified for both ligand-bound MOR systems (Supplementary Figs 3 and 4), and the equilibrium probabilities of the 14 macrostates were calculated (Supplementary Fig. 5a,b). To understand whether the identified 14 kinetic macrostates corresponded structurally to inactive, activated, or intermediate conformational states along the MOR activation pathway, the trajectory frames corresponding to each macrostate were projected onto order parameters describing some of the largest conformational changes occurring upon activation, namely the TM6 outward motion relative to TM3, and the backbone distortion involving the N^7.49^ PxxY^7.53^ motif at the cytoplasmic end of TM7. The former was represented by the distance between the Cα atoms of residues R165^3.50^ and T279^6.34^ while the latter was measured as the root mean square deviation (RMSD) of the N332^7.49^ PxxYA337^7.54^ segment from the corresponding region in the inactive MOR crystal structure.

**Figure 1:**
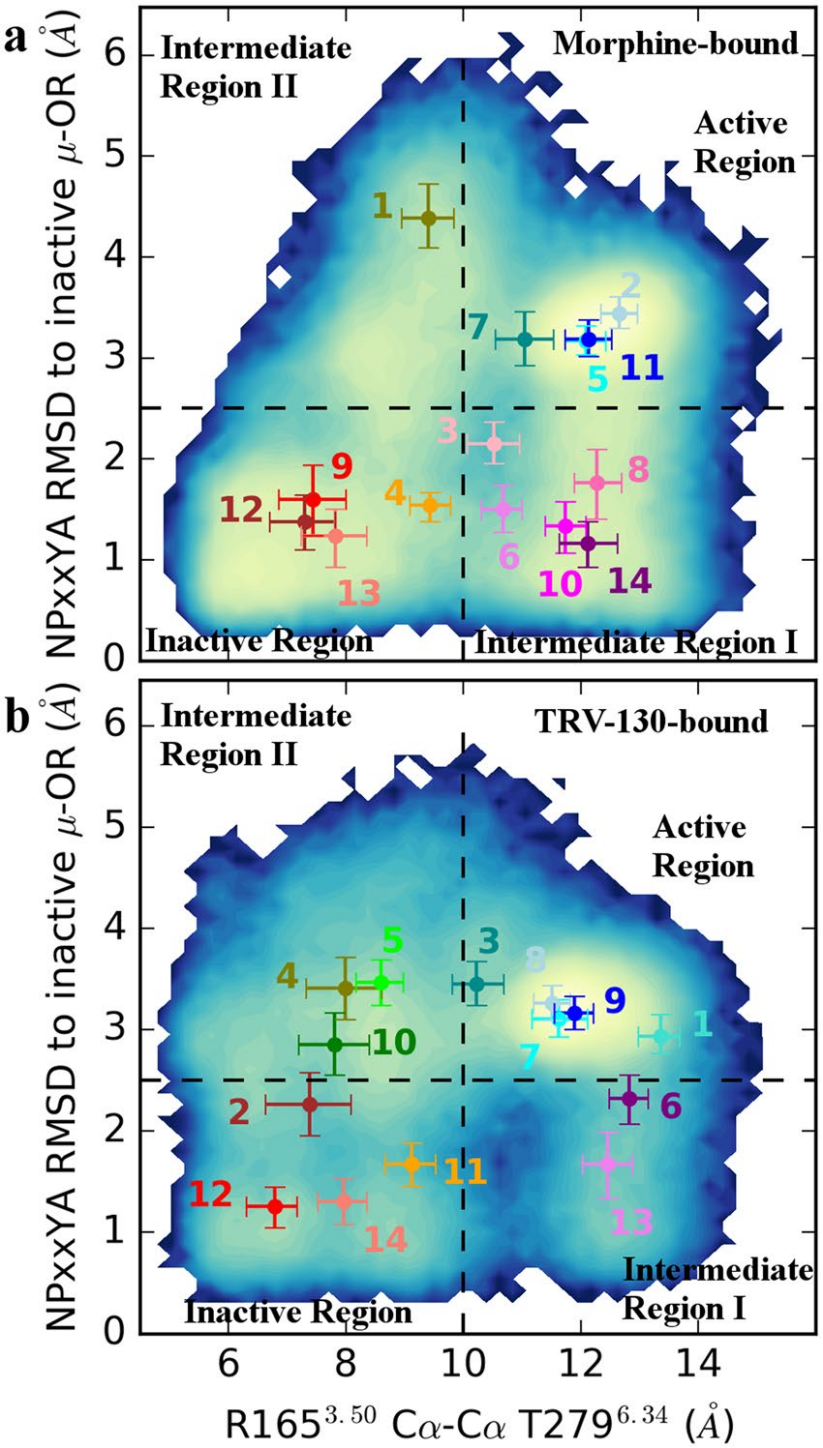
Projection of kinetic macrostates onto density maps of order parameters known to describe receptor activation and sampled during simulation of the (**a**) morphine-bound and (**b**) TRV-130-bound MOR systems. Order parameters are: the TM6 outward motion relative to TM3 (represented by the R165^3.50^-T279^6.34^ Cα distance, on the x axis), and the backbone distortion involving the NPxxY motif at the TM7 cytoplasmic end (represented by the RMSD of the N332^7.49^-A337^7.54^ segment from the corresponding region in the inactive MOR crystal structure, on the y axis). The two-dimensional histograms represent the count density of these parameters calculated for each system, with increasing density values shown from blue to yellow. Dotted black lines define the boundaries of conformational regions characterized by high density of these order parameters (yellow regions). Specifically, these correspond to active and inactive crystallographic states of MOR, as well as intermediate states in regions I and II. Dots represent averages (reweighted according to the MSM stationary probability) of the two order parameters in each of the 14 macrostates identified based on kinetic similarity. These macrostates are numbered as in Supplementary Fig. 5 and are colored based on the region they fall within. Specifically, macrostates in the “Active region”, the “Inactive region”, “Intermediate region I” and the “Intermediate region II” are colored in shades of blue, red/orange, magenta, and green, respectively. Vertical and horizontal bars refer to the first (lower bound) and third (upper bound) quartiles of the distributions of the two order parameters in each macrostate.

Figure 1a and b show two-dimensional histograms of the count density of these two order parameters sampled during simulation of morphine-bound and TRV-130-bound MOR, respectively. The high density regions of these parameters define four regions of the conformational space. These four regions correspond to states that are structurally close to the inactive MOR crystal structure, to the activated MOR crystal structure, and to two intermediate conformational states between these inactive and activated MOR structures, herein called intermediate I and II regions (see Fig. 1). Averages (reweighted according to the MSM stationary probability) of the two order parameters in each of the 14 kinetic macrostates are represented by colored dots.

Based on calculated equilibrium probabilities of the identified 14 kinetic macrostates for the morphine-bound MOR system (Supplementary Fig. 5a), the overall steady-state probability for the inactive region (macrostates #4, #9, #12, and #13) of this system is 50% (45%, 54%), while the probabilities of the active (#2, #5, #7, and #11), intermediate I (#3, #6, #8, #10, and #14), and intermediate II (#1) regions are 16% (14%, 18%), 32% (28%, 35%), and 0.083% (0.076%, 0.089%), respectively. For the TRV-130-bound receptor (Supplementary Fig. 5b), the respective probabilities of the inactive (#2, #11, #12, #14), active (#1, #3, #7, #8, and #9), intermediate I (#6, #13), and intermediate II (#4, #5, and #10) regions are 58% (55%, 60%), 22% (18%, 27%), 12% (11%, 13%), and 6.6% (6.1%, 7.2%), respectively. Notably, the most probable kinetic macrostates of the morphine-bound and TRV-130-bound MOR systems (#12 and #14, respectively; see Supplementary Fig. 5a and b, respectively) fall within the “Inactive region” of Fig. 1. Accordingly, the kinetic macrostate #12 of morphine-bound MOR is characterized by TM6 in a conformation that is structurally close to the inactive MOR crystal structure, with a less than 1 Å divergent R165^3.50^-T279^6.34^ Cα atom distance (Fig. 1a), but an entirely different conformation of ICL3 compared to the structure revealed by the inactive δ-opioid receptor (DOR) crystal structure^29^. In contrast, while still being closer to the inactive than the activated MOR crystal structure, the representative conformation of the most probable kinetic macrostate of TRV-130-bound MOR (macrostate #14) shows a conformation of TM6 that is intermediate between the inactive and activated MOR crystal structures, with a R165^3.50^-T279^6.34^ Cα atom distance that is, on average, 1.5 Å larger than the corresponding distance in the inactive MOR crystal structure (Fig. 1b), and a broken hydrogen bond between the residue sidechains.

**Figure 2:**
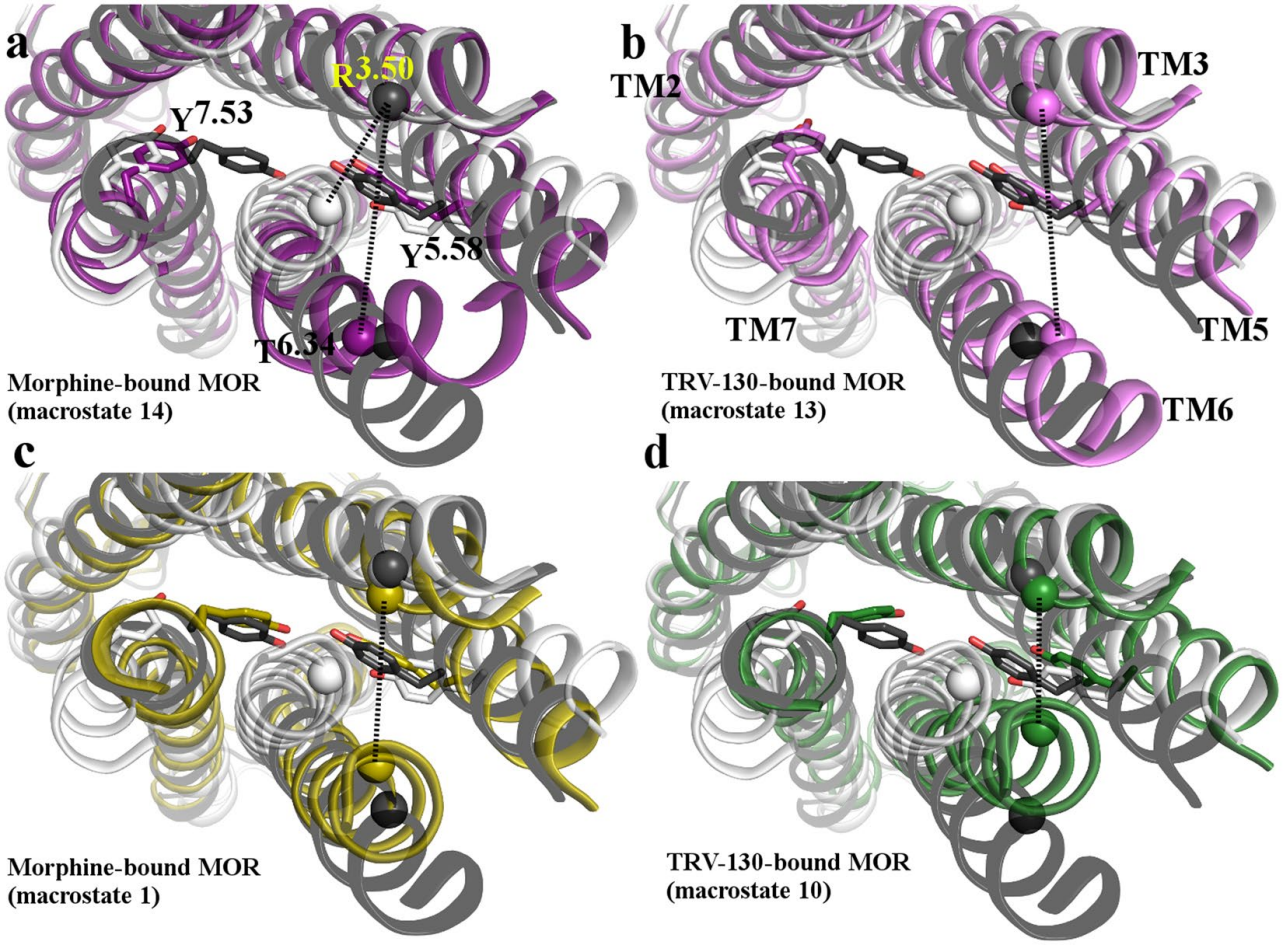
Structural superposition between representative conformations of the most probable kinetic macrostates of intermediate regions I and II of the morphine-bound and TRV-130-bound MOR systems with inactive and activated MOR crystal structures. Cytoplasmic views of overlaps of the active (gray) and inactive (white) crystal structures of MOR with representative structures of: (**a**,**c**) the most probable kinetic macrostates #14 and #1 found in the intermediate region I and II, respectively, sampled by morphine-bound MOR, and (**b**,**d**) the most probable kinetic macrostates #13 and #10 located in the intermediate region I and II, respectively, sampled by TRV-130-bound MOR. Residues Y252^5.58^ and Y336^7.53^ are shown as sticks. Cα atoms of residues R165^3.50^ and T279^6.34^ are shown as spheres, and are connected by dotted line. TM1, TM4, helix 8 and intracellular loops are not shown for clarity.

While roughly the same number of kinetic macrostates represents overall structural features of active or inactive MOR conformations in both morphine-bound and TRV-130-bound MOR systems (see Fig. 1a and b, respectively), the two intermediate regions contain a different number of macrostates, revealing a different kinetic behavior of the two systems. Notably, all macrostates belonging to these intermediate regions also differ substantially from the currently available crystal structures of MOR. Figure 2 shows overlaps between active (gray) and inactive (white) crystal structures of MOR relative to representative structures of the most probable kinetic macrostates found in the intermediate regions I and II sampled by morphine-bound MOR (#14 and #1, respectively) and TRV-130-bound MOR (#13 and #10, respectively).

As illustrated in Fig. 2a,b, in representative structures of the most probable kinetic macrostates of the intermediate region I (i.e., #14 for morphine-bound MOR and #13 for TRV-130-bound MOR), the backbone of the TM7 N^7.49^ PxxY^7.53^ region is close to that of the inactive MOR conformation, with the side chain of Y336^7.53^ facing TM2 and also adopting a conformation similar to the one observed in the inactive MOR crystal structure. In both these intermediate I states (Fig. 2a and b), TM6 assumes an active-like conformation while TM5 can adopt an intermediate conformation between inactive and active MOR crystal structures, as judged by values of the R165^3.50^-T279^6.34^ Cα-Cα and R165^3.50^-Y252^5.58^ sidechain Cζ -OH distances, respectively (compare pink-shaded intermediate region I macrostates with the red inactive or blue active macrostates in Supplementary Fig. 6). Notably, comparison between the R165^3.50^-Y252^5.58^ sidechain distances in the kinetic macrostates of morphine-bound (Supplementary Fig. 6a) and TRV-130-bound (Supplementary Fig. 6b) MOR reveals a region of the conformational space that is only accessible to the third most probable (Supplementary Fig. 5a), inactive (Fig. 1a) kinetic macrostate of the morphine-bound MOR (i.e., macrostate #13). In this state, the Y252^5.58^ side chain adopts an alternative, lipid exposed orientation compared to either MOR crystal structure.

In contrast to representative conformations of the intermediate region I, representative structures of the most probable kinetic macrostates in the intermediate region II (i.e., #1 and #10 for the morphine-bound and TRV-130 bound receptor, respectively; see Fig. 2c and d), exhibit an N^7.49^PxxY^7.53^ conformation similar to that seen in the active MOR crystal structure, with the Y336^7.53^ side chain pointing in the space between TM3 and TM6, while TM6 adopts a conformation that is intermediate between the MOR inactive and active crystal structures.

### Morphine and TRV-130 induce distinct conformational rearrangements of the receptor’s intracellular region during MOR activation

The majority of top correlated motions in the morphine-bound and TRV-130-bound MOR systems identified by tICA coordinates 1–10 suggest slowest motions at the receptor’s cytoplasmic side (Supplementary Tables 1–10). However, the specific top correlated residue pairs are different in the two systems, especially according to the most dominant tICA coordinates 1–3. Supplementary Figure 7 shows the projection of the simulation data onto the first two tICA components, along with their average values in each of the macrostates. While in morphine-bound MOR the highest correlated motions identified by tICA coordinates 1 and 2 mostly involve residue pairs within TM6 or between TM6 and TM3/TM5 (Supplementary Table 1 and 2, respectively), the corresponding highest correlated motions in TRV-130-bound MOR involve residues at the cytoplasmic end of TM5 as well as residues within TM1, TM2, TM3, TM7, and helix 8.

The highest correlated motion identified by tICA component 1 that involves side chain residues on different helices of morphine-bound MOR draws attention to changes in the L259^5.65^-K271^6.26^ distance (see Supplementary Table 1), which assumes values of 4.5 Å and 6.2 Å in the inactive and activated MOR crystal structures, respectively. As shown in Supplementary Figs 7a and 8a, changes in the L259^5.65^-K271^6.26^ residue pair separate macrostates #14 and #10 of the intermediate region I of morphine-bound MOR from the other identified kinetic macrostates. This and other changes identified by tICA component 1 are linked to a pronounced bending at the cytoplasmic end of TM6, which is seen in all kinetic macrostates of the intermediate region I of morphine-bound MOR, with the exception of macrostate #8. In the most probable of these kinetic macrostates (i.e., macrostate #14 of morphine-bound MOR; see Supplementary Fig. 9a), this TM6 bending is accompanied by a rearrangement of TM6 residue sidechain with respect to the active MOR crystal structure, the breaking of the intra-helical backbone hydrogen bond between residues T279^6.34^-L283^6.38^, and the formation of a hydrogen bond between the backbone amino group of L283^6.38^ and the backbone carbonyl group of I278^6.33^ (Supplementary Fig. 10a). A different rearrangement of TM6 residues is observed in macrostate #10 of the morphine-bound MOR (Supplementary Fig. 9b), which is separated from macrostate #14 by tICA 2 as clearly shown by the distribution of the highest correlated motion involving residue pair M281^6.36^-Y252^5.58^ in the various macrostates (Supplementary Figs 7a and 8b). In this kinetic macrostate of morphine-bound MOR, TM6 bending is accompanied by the breaking of intra-helical backbone hydrogen bonds between residues L283^6.38^-A287^6.42^ and V284^6.39^-V288^6.43^ (Supplementary Figs 9b and 10b).

Not only is this TM6 bending and intra-helical backbone hydrogen bond rearrangement not observed in any of the identified kinetic macrostates of the TRV-130-bound MOR (e.g., see the most probable macrostate #13 of the intermediate region I in Supplementary Fig. 9c and Supplementary Fig. 10c,d), but the top correlated inter-helical residue pairs identified by tICA component 1 (Y252^5.58^-Y336^7.53^) and 2 (T120^2.56^-N150^3.35^) appear to be unique to TRV-130-bound MOR (Supplementary Tables 1 and 2). Minimum sidechain heavy atom distances between Y252^5.58^-Y336^7.53^ and T120^2.56^-N150^3.35^ in the various macrostates of TRV-130-bound MOR are shown in Supplementary Fig. 11a and b, respectively. While the available inactive and activated crystal structures of MOR have drawn attention to the importance of changes in the Y252^5.58^-Y336^7.53^ distance, the high T120^2.56^-N150^3.35^ correlation revealed by tICA 2 is specific to TRV-130-bound MOR. As shown in Supplementary Fig. 11b, the T120^2.56^-N150^3.35^ sidechain distance separates green-shaded kinetic macrostates, which include intermediate region II conformations of TRV-130-bound MOR, from most macrostates. Notably, while the coordinated conformational change between the active and inactive region is captured mainly by tICA 1 in TRV-130-bound MOR (see Supplementary Fig. 7b), the tICA 2 component also draws attention to the slow motion of helix 8 (e.g., see L88^1.52^-F350 in Supplementary Table 2) in TRV-130-bound MOR. As seen in the majority of macrostates of intermediate region II and the active region of TRV-130-bound MOR, helix 8 is tilted towards the cytosol compared to its orientation in the MOR inactive crystal structure, which is more parallel to the membrane (Supplementary Fig. 12a).

Differences in the top correlated inter-helical residue pairs between morphine-bound and TRV-130-bound MOR are also suggested by tICA 3 (Supplementary Table 3), with highest correlations involving residues of ICL1 in the morphine-bound receptor (e.g., M99^ICL1^-I105^2.41^; see distribution in the different macrostates in Supplementary Fig. 8c) or TM5/TM6 residues within the ligand binding pocket (e.g., A240^5.46^-H297^6.52^; see distribution in the different macrostates in Supplementary Fig. 11c) of TRV-130-bound MOR. While most kinetic macrostates of morphine-bound MOR adopt the same conformation of ICL1 seen in the active MOR crystal structures, macrostate #5 exhibits a substantially different conformation (Supplementary Fig. 12b). In this state, residue M99 loses contacts with residues in TM1 and TM2 (e.g., I105^2.41^, as seen in MOR active crystal structure) whereas other ICL1 residues, in particular K98 and K100, become more exposed to the cytosol.

Not only was this ICL1 conformation not explored in TRV-130-bound MOR, but the tICA 3 of TRV-130-bound MOR captured different changes, specifically in the ligand-binding pocket. For instance, an increased minimum distance between sidechains of residues A240^5.46^ and H297^6.52^, which are 4.4 Å and 4.0 Å in the inactive and activated MOR crystal structures, respectively is mostly seen in macrostates of the TRV-130-bound MOR corresponding to the inactive region (Supplementary Fig. 11c). In particular, in the most probable of the macrostates showing largest changes in the A240^5.46^-H297^6.52^ distance (macrostate #11) in TRV-130-bound MOR, the H297^6.52^ sidechain moves inwards into the orthosteric pocket whereas the backbone of TM5 in the region around A240^5.46^ moves away from TM6. This is accompanied by a change in the sidechain dihedral angle of the toggle switch^15^ residue W293^6.48^, which is rotated towards TM5.

Notably, the lower the correlation between residue pairs the higher the occurrence of slowest conformational degrees of freedom that are the same between the morphine-bound and the TRV-130-bound MOR systems (see green labels in Supplementary Tables 4–10).

### Morphine and TRV-130 transfer information differently across the receptor

**Figure 3:**
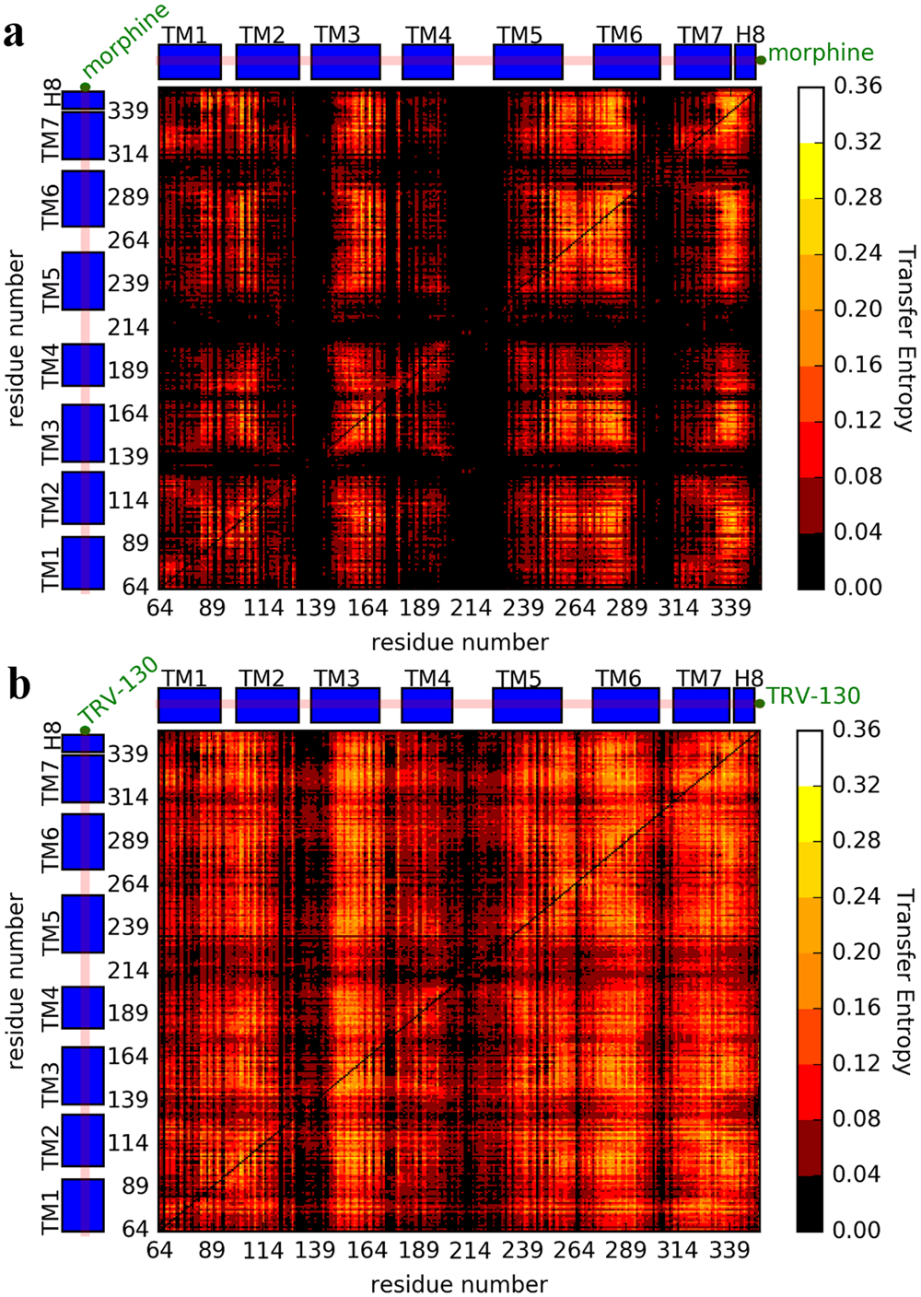
Pairwise transfer entropy matrices calculated for the two simulated ligand-bound MOR systems. Specifically, these matrices are calculated from simulation of (**a**) the morphine-bound receptor and (**b**) the TRV-130-bound receptor. Each column of the matrix represents the information the column residue j transfers to the row residue i, whereas each row of the matrix represents the information the row residue i receives from column residue j.

To understand how the G protein biased ligand TRV-130 affects the allosteric communication across the receptor compared to the classical opioid ligand morphine, we computed the transfer entropy between each pair of receptor residues, as well as receptor residues and the ligand, for each of the simulated ligand-bound MOR systems (see Methods for details). Figures 3a and b show the pairwise transfer entropy matrix for the morphine-bound and TRV-130-bound MOR, respectively. The analysis highlights striking differences in the information dynamics in the two ligand-bound MOR systems with a bi-directional flow of information involving different receptor regions. For instance, not only do TM6 helices transfer information to different regions of the receptor (e.g., compare TM6 region on the columns of the matrix in Fig. 3a and b) but they also receive information from different receptor regions (e.g., compare TM6 region on the rows of the matrix in Fig. 3a and b). In general, there appears to be more transfer of information across the receptor in TRV-130-bound MOR than morphine-bound MOR. In the latter system, certain parts of the receptor, particularly the three extracellular loop regions (i.e., regions between TM2-TM3, TM4-TM5, and TM6-TM7 on the columns of the matrix in Fig. 3a), are not involved in any information transfer whereas the extracellular ends of TM2, TM3, TM5, and TM6, as well as entire TM4, appear to receive information from other parts of the receptor (see corresponding rows of the matrix in Fig. 3a).

**Figure 4:**
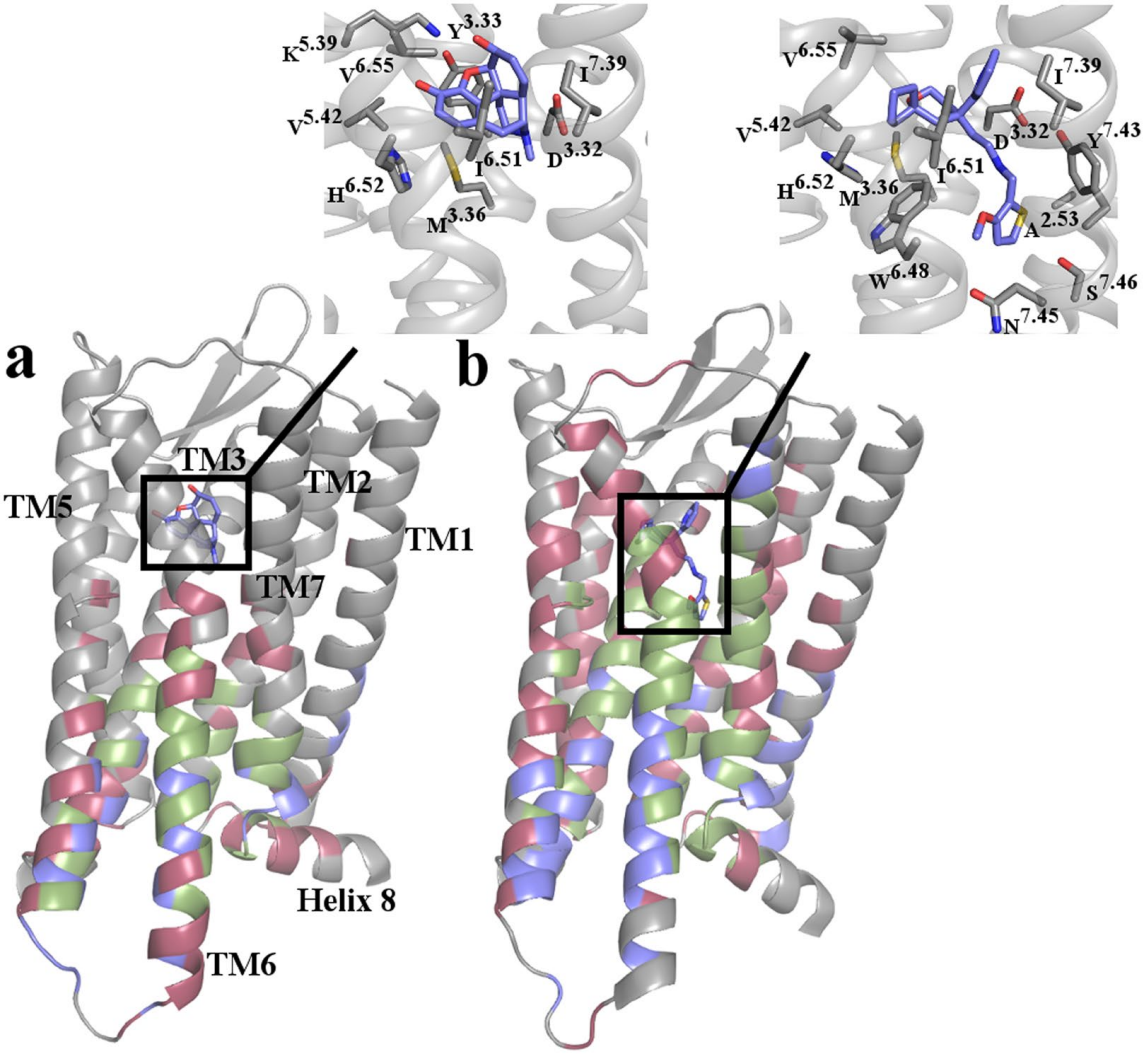
Spatial distribution of top transmitters, receivers, and connectors across the two simulated ligand-bound MOR systems. Identified top transmitters (blue), receivers (red), and connectors (green) are mapped onto cartoon representations of starting conformations of **(a)** morphine-bound MOR and **(b)** TRV-130-bound MOR. Morphine and TRV-130 are shown as sticks with their carbon atoms colored in blue. Figures in insets highlight the MOR residues within 3.5 Å of the starting conformation of morphine or TRV-130, respectively. Residues are shown as sticks with their carbon atoms colored in gray.

To single out key transmitter and receiver residues, the calculated pairwise transfer entropy values were used as weights for the edges of a directed graph representation of the protein residues and the ligand, and the information transfer contribution of each receptor residue and the ligand ranked. Specifically, two scores were assigned to each residue: an authority score, which estimates the overall importance of a residue as a “receiver” of information, and a hub score, which reflects the residue’s overall importance as a “transmitter” of information (see Methods for details). Residues that are both good receivers and good transmitters, as determined by high authority and hub scores, are called “connectors”. These residues are expected to play an important role in the allosteric coupling between different receptor regions, or in modulating ligand bias. The top transmitters, receivers, and connectors in the two-ligand bound MOR systems are listed in Supplementary Tables 11, 12 and 13, respectively, and their location on the ligand-bound MOR systems is shown in Fig. 4.

Consistent with the transfer entropy matrices shown in Fig. 3, fewer residues are identified as key transmitters, receivers, and connectors in the morphine-bound MOR compared to the TRV-130-bound MOR, and no key transmitters, receivers, or connectors are identified in the receptor extracellular regions as well as on the entire TM4 (Supplementary Tables 11–13 and Fig. 4). The distribution of key residues around the ligand is also strikingly different in the two-ligand bound MOR systems (Fig. 4a and b), with several of the residues within and around the ligand-binding pocket identified as key residues in TRV-130-bound MOR, but not in morphine-bound MOR (see Supplementary Tables 11–13 for details). For instance, residues within 6 Å from TRV-130 that are identified as connectors in the TRV-130-bound, but not the morphine-bound, MOR system are: Y148^3.33^, I296^6.51^, H297^6.52^, W318^7.35^, I322^7.39^, and Y326^7.43^ (see Supplementary Table 13).

Transfer entropy analysis also reveals differences in the way the ligands TRV-130 and morphine communicate with receptor residues (Supplementary Fig. 13). Specifically, while the TRV-130 molecule is identified as a good transmitter (Supplementary Fig. 13b and hub value of 0.65), but a poor receiver (its authority score of 0.09 is the lowest calculated value), morphine is identified as a weaker transmitter (hub value of 0.55) but a stronger receiver (authority value of 0.33; Supplementary Fig. 13a) than TRV-130.

Another set of residues that may play an important role in biased information flow are those that are assigned to a given group (i.e., transmitter, receiver, or connector) in one ligand-bound receptor system, but a different one in the other. Notably, several residues that undergo conformational changes upon receptor activation (e.g., the toggle switch residue W293^6.48^ and the conserved core triad^15^ residue I155^3.40^) and/or participate in the extensive network of polar interactions between the orthosteric binding pocket and the G protein-binding interface that rearranges upon activation as seen in the few available activated GPCR crystal structures^15^ (e.g., W293^6.48^, N328^7.45^, and S329^7.46^ as well as the Na^+^ ion-coordinating residues D114^2.50^, N150^3.35^, and S154^3.39^) are identified as receivers in morphine-bound MOR but as connectors in TRV-130-bound MOR (see red residues in Supplementary Table 13). Notably, the change of the toggle switch residue W293^6.48^ from receiver in the morphine-bound MOR to connector in TRV-130-bound MOR is accompanied by an increased flexibility of the sidechain in several kinetic macrostates of TRV-130-bound MOR.

Finally, several residues that change their role from connectors in morphine-bound MOR to either transmitters or receivers in TRV-130-bound MOR are located at the intracellular end of TM6 (e.g., N274^6.29^, T279^6.34^, L283^6.38^, V285^6.40^, and V286^6.41^; see red and green labels in Supplementary Table 13). These residues are also involved in the slowest conformational degrees of freedom accessible to morphine-bound MOR as assessed by tICA components 1 and 2 (see Supplementary Tables 1 and 2). As shown in Fig. 4, while the TRV-130-bound MOR system has more transmitters at the intracellular end of TM6, the morphine-bound MOR system has more receivers and connectors.

### Ligand-specific activation pathways of MOR

**Figure 5:**
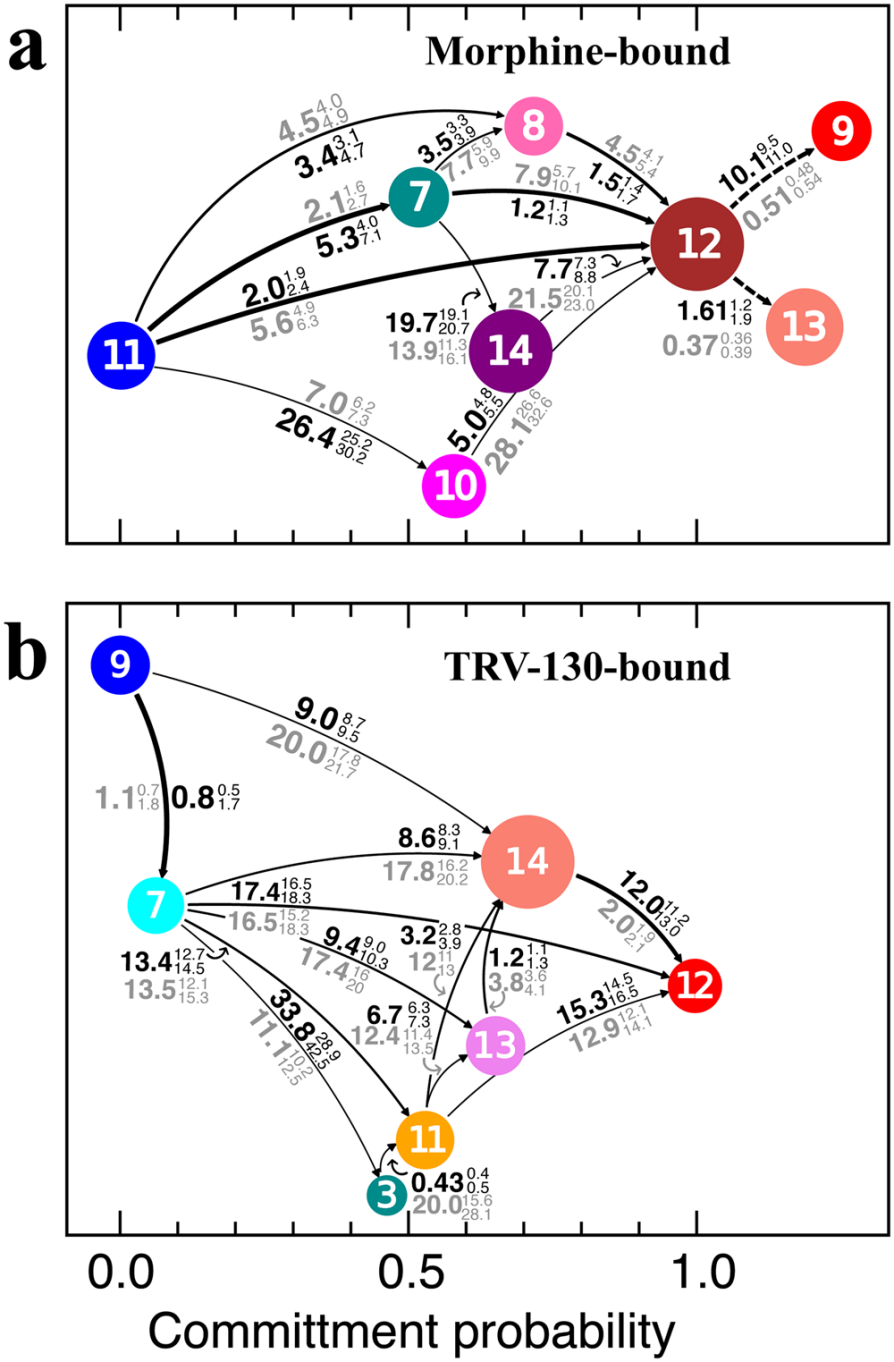
Deactivation/activation pathways of morphine-bound and TRV-130-bound MOR systems. Transition path theory-predicted pathways with more than 1% of the flux linking (**a**) the active-like kinetic macrostate #11 and the inactive-like macrostate #12 of morphine-bound MOR, and (**b**) the active-like macrostate #9 and the inactive-like macrostate #12 of TRV-130-bound MOR. Arrow thickness is proportional to the net fluxes between two kinetic macrostates. Macrostates are represented as circles of size proportional to their equilibrium probability and are color-coded as in Fig. 1. Numbers next to each arrow represent the mean first passage time (in units of µs) to go from one macrostate to the other in the direction of the arrows (black) or in the reverse direction (gray). First and third quartiles are indicated as superscript and subscript, respectively. Dotted lines in panel (**a**) connect alternative inactive macrostates #9 and #13 that can be reached from the active state only from macrostate #12.

Transition Path Theory was used to investigate the pathways between the identified kinetic macrostates of the morphine-bound or TRV-130-bound MOR systems that are most similar to the available inactive and active crystal structures of MOR as assessed by RMSD calculated on Cα atoms of the TM region only. For the morphine-bound MOR, these macrostates are the active-like macrostate #11 and the inactive-like macrostate #12 (average RMSD of 1.6 ± 0.1 Å and 1.5 ± 0.1 Å from the active and inactive crystal structures, respectively). Supplementary Table 14 lists the flux associated with different activation/deactivation pathways connecting these two macrostates of morphine-bound MOR, while Fig. 5a reports the mean-first passage times (MFPTs) for transitions between all kinetic macrostates involved in these pathways.

The two most probable pathways of the morphine-bound MOR, accounting for over 70% of the flux (Supplementary Table 14), involve either a direct transition to the inactive-like macrostate #12, or one that is interposed by the active-like macrostate #7, in which TM6 has moved on average ~1.3 Å away from its position in the active macrostate #11 (see different values of the R165^3.50^-T279^6.34^ Cα-Cα distance in Fig. 1a) towards the inactive macrostate #12. The other most probable pathways, accounting for ~20% of the flux (Supplementary Table 14), involve transitions through a low equilibrium probability state (macrostate #8) of the intermediate region I of the morphine-bound MOR. Other two transition pathways are also observed in morphine-bound MOR, but they exhibit a much smaller flux (Supplementary Table 14). Specifically, these pathways proceed to the inactive state of the receptor through slow transitions to macrostates in the intermediate region I exhibiting a bent helix TM6, specifically: macrostate #14 (i.e., the most probable macrostate of intermediate region I) and macrostate #10. These transitions are characterized by slow dynamics along tICA components 1 and 2, and by much longer MFPTs (20–26 µs) from the active-like macrostate #11 (Fig. 5a) compared to the pathways involving other intermediate region I macrostate #8 (3–5 µs), which does not feature a TM6 bending.

Notably, the other two inactive-like macrostates (#9 and #13) of morphine-bound MOR that are structurally very similar to macrostate #12 cannot be reached directly from the active macrostate #11 but rather passing through macrostate #12 (indicated by dotted lines in Fig. 5a). Unlike macrostate #12, macrostate #9 exhibits an ICL3 conformation that is similar to that seen in the ultra-high resolution DOR crystal structure^29^ (Supplementary Fig. 14). In this conformation, the side chains of ICL3 residues A264 and M265 are directed towards the helical bundle, forming hydrophobic interactions with TM3, TM5, and TM6 residues (Supplementary Fig. 14a). Notably, while the direct transition from the active macrostate #11 to macrostate #12 is rather fast (MFPT of 2.0 µs; see Fig. 5a), the conformational rearrangement of the ICL3 loop to adopt the crystal-like conformation seen in the inactive macrostate #9 requires longer times (MFPT over 10 µs). Transition Path Theory analysis also shows that the system can also quickly transition (MFPT 1.6 µs) from macrostate #12 to the other identified inactive-like macrostate #13, in which the Y252^5.58^ side chain adopts an alternative, lipid exposed orientation, compared to either MOR crystal structures.

For the TRV-130-bound MOR system, the transition was analyzed between macrostates #9 and #12, which exhibited average RMSD values of 1.8 ± 0.1 Å and 1.7 ± 0.1 Å from the active and inactive MOR crystal structures, respectively. Supplementary Table 15 lists the flux associated with different pathways connecting these two states in TRV-130-bound MOR, while Fig. 5b reports the MFPTs for transitions between all kinetic macrostates involved in these pathways.

The most probable pathway linking the active-like macrostate #9 and the inactive-like macrostate #12 of TRV-130-bound MOR (Supplementary Table 15 and Fig. 5b), accounts for ~32% of the flux. This transition is not direct but rather involves an intermediate active-like state (macrostate #7; Supplementary Table 15), which is characterized by TRV-130 adopting several kinetically similar, but conformationally distinct, binding poses within the orthosteric binding site of MOR. Compared to the TRV-130-MOR interactions in the active-like macrostate #9, in macrostate #7, TRV-130 forms a reduced number of hydrophobic interactions with the sidechains of the conserved triad residue I155^3.40^, as well as with the residues in the sodium binding pocket, i.e. D114^2.50^, N150^3.35^, and S154^3.39^ (Supplementary Fig. 15). Notably, these residues are also identified as connectors in TRV-130-bound MOR but as receivers in morphine-bound MOR (Supplementary Table 13). In the high flux pathways, the inactive macrostate #12 of TRV-130-bound MOR is reached from the active-like macrostate #7 either directly or transitioning through macrostates of the intermediate region I (macrostate #13) or the inactive region (macrostates #11 and #14; Supplementary Table 15). Approximately 64% of the flux to the MOR inactive state involves the most probable kinetic macrostate of the TRV-130-bound system (macrostate #14).

Although pathways that do not involve transitions to the intermediate regions are the single pathways with largest flux in this analysis, approximately 60–70% of transitions do include states in these regions. Inspection of the kinetic evolution of the occupation probability of the different macrostates starting from the inactive receptor (Supplementary Fig. 16) suggests that these intermediate states are indeed important states and play a different role in the two ligand-receptor MOR systems. For instance, at least one macrostate (i.e., macrostate #14) of the intermediate region I of the morphine-bound MOR system is populated with significant probability (~20%) after ~10 µs whereas no intermediate state is significantly populated in the TRV-130-bound MOR at any time before the system reaches its equilibrium probability starting from its inactive state.

## Discussion

Seven important observations about the MOR activation process and elements of biased agonism can be derived from the study reported here. First off, while adaptive sampling allows a complete transition between activated and inactive crystal structures of MOR, the simulations reported herein reveal that the R165^3.50^-T279^6.34^ hydrogen bond, which is equivalent to the TM3-TM6 salt-bridge in other GPCRs, is mostly broken in both the morphine-bound and the TRV-130-bound MOR systems, including inactive states of the receptor. Although the crystal structures of inactive opioid receptors suggested a formed hydrogen bond between R3.50 and T6.34, the results of recent MD simulations of inactive opioid receptors^28^ showed that unlike the R3.50-D3.49 salt bridge, the R3.50-T6.34 hydrogen bond is very dynamic and is found to be broken at times. This observation is corroborated by the free-energy evaluations reported here and is also in line with earlier experimental observations that the inter-helical TM3-TM6 interaction in rhodopsin-like GPCRs breaks more easily than the intra-helical salt bridge between R3.50 and D3.49^30^.

A second important observation is the existence of two distinct intermediate metastable regions in addition to those closely resembling activated and inactive crystal structures of MOR. Notably, these two intermediate regions contain a different number of kinetic macrostates in the morphine-bound and TRV-130-bound MOR systems, revealing a different dynamic behavior of the receptor bound to a classical opioid agonist or a G protein-biased agonist. These intermediate macrostates differ substantially from the currently available crystal structures of MOR, and may be particularly useful for *in silico* identification of novel functionally selective ligands that may eventually be developed into improved drugs. Although previous MD simulations of the β2-adrenergic receptor (β2AR) deactivation process^31^ and ligand-free M2 muscarinic receptor (M2R) activation^32^ had also been able to sample inactive and activated crystallographic states of these receptors, they identified only one of the two intermediate metastable regions revealed by the simulations reported here, specifically intermediate region I in the case of β2AR and intermediate region II for M2R. Since the simulations reported here identified both intermediate region I and II metastable states, we wonder whether this feature is specific to opioid receptors or may be dependent on exhaustive conformational sampling.

A third observation is that the most probable kinetic macrostates of the morphine-bound and TRV-130-bound MOR systems correspond to inactive-like conformations of the receptor with alternative ICL3 conformations, which are more extended towards the cytoplasm compared to the corresponding region in the inactive δ-OR crystal structure^29^. While the latter is the only ICL3 crystal structure of an opioid receptor available to date, it may or may not be representative of the most stable conformation of the ICL3 region in inactive MOR. Notably, equilibrium probabilities from our simulations suggest otherwise, at least in the presence of an agonist at the orthosteric binding pocket. The fact that the receptor with either morphine or TRV-130 at the orthosteric binding pocket is found to populate, with significantly higher probability, conformational states characterized by inactive-like structural features is in agreement with recently published evidence suggesting that while opioid agonist binding initiates the activation process^22^, binding of G protein mimetics is required for full MOR activation.

The fourth observation is that the slowest dynamic motions in the morphine-bound and TRV-130-bound MOR systems are substantially different. While in morphine-bound MOR the slowest motions involve the cytoplasmic ends of TM6, TM3, and TM5 only, the dynamic behavior of TRV-130-bound MOR is much more diffuse, with highest correlated motions involving residues in TM1, TM2, TM3, TM5, TM7, and helix 8. While a pronounced bending occurs at the cytoplasmic end of TM6 in morphine-bound MOR, as a result of the rearrangement of TM6 residue sidechain, as well as the breaking and formation of intra-helical backbone hydrogen bonds, this TM6 bending and intra-helical backbone hydrogen bond rearrangement is not seen in any of the identified kinetic macrostates of TRV-130-bound MOR. Differences between the morphine-bound and the TRV-130-bound MOR systems also pertain to ICL1 conformational rearrangements in the morphine-bound receptor or changes involving TM5/TM6 residues within the ligand binding pocket in TRV-130-bound MOR. The specifics of these changes provide testable hypotheses of molecular determinants that are involved in biased agonism.

The fifth observation is that the G protein-biased ligand TRV-130 affects the allosteric communication across MOR differently from the classical opioid ligand morphine, with a generally more pronounced transfer of information across the receptor in TRV-130-bound MOR than morphine-bound MOR. The analysis highlights a number of good receivers, transmitters, and/or connectors that are expected to play an important role in the allosteric coupling between different receptor regions, or in modulating ligand bias. Overall, morphine-bound MOR has fewer key transmitters, receivers, or connectors compared to TRV-130-bound MOR, and none in the receptor extracellular regions or TM4. Moreover, different combinations of transmitters, receivers, and connectors are seen at the intracellular end of TM6, with more transmitters in this region of the TRV-130-bound system compared to the morphine-bound MOR system. Notably, some of the residues that are transmitters in the TRV-130-bound MOR system are connectors in the morphine-bound receptor complex (i.e., T279^6.34^, L283^6.38^, V285^6.40^, and V286^6.41^), suggesting a more expanded role of these specific residues in morphine-bound MOR. Given the involvement of the intracellular end of TM6 in the main conformational changes leading to receptor activation, it does not come as a complete surprise that this region contains several centers of information transfer, and that they have different roles in the two ligand-bound MOR systems, possibly highlighting elements of functional selectivity.

Several of the key residues identified within and around the ligand-binding pocket are also different between the TRV-130-bound and morphine-bound MOR systems, drawing attention to specific residues as connectors in the TRV-130-bound, but not the morphine-bound, MOR system. Two of these residues, specifically W318^7.35^ and Y326^7.43^, were shown to control ligand bias in a recent experimental study of MOR^34^. Among the residues that belong to a given group (i.e., transmitter, receiver, or connector) in TRV-130-bound MOR, but a different one in morphine-bound MOR, is the Na^+^-coordinating residue N150^3.35^. Not only was mutation of this residue shown to modulate agonist binding in DOR, but it also led to enhanced β-arrestin constitutive activity^29^. Another interesting residue that is identified as a connector in TRV-130-bound MOR, but as a receiver in morphine-bound MOR, is the so-called toggle switch residue W293^6.48^. The sidechain of this residue was seen to adopt multiple conformations in several TRV-130-bound MOR kinetic macrostates, but not in morphine-bound MOR. Interestingly, the conformational rearrangement of W^6.48^ upon phenylalanine mutation of G^7.42^, another identified connector in TRV-130-bound MOR, was recently shown to produce G-protein biased signaling in the CCR5 receptor^35^.

The sixth observation is that multiple activation/deactivation pathways are identified in both morphine-bound and TRV-130-bound MOR. Notably, for both systems, only a very small fraction of these pathways involve transition to the inactive MOR state through intermediate region II macrostates, while most indirect pathways proceed through intermediate region I. Moreover, although both inactive macrostates of the morphine-bound and TRV-130-bound MOR do not adopt an ICL3 conformation analogous to that of DOR, the receptor inactive state can be reached faster in the case of morphine-bound MOR than the TRV-130-bound system.

The seventh and last observation is that the calculated time scales describing transitions between active-like and inactive-like MOR conformations in the presence of bound agonists are tens of microseconds. These results are consistent with very broad order-of-magnitude estimates (i.e., microseconds to low milliseconds) derived from NMR peak intensity obtained for MOR in the presence of agonists^22^. Also interesting is that rearrangements of the intracellular regions of the receptor (i.e., ICL1, ICL3, and H8) are identified among the slowest dynamical features. This behavior has also been observed in the recently published NMR spectra of MOR^22^, which revealed that the most prominent dynamical changes of the agonist bound receptor in the absence of G protein mimetics involve loop ICL1 and H8.

Notably, for the morphine-bound receptor, the slowest calculated time scales from the kinetic model do not refer to the largest conformational change (i.e. the outward movement of TM6 relative to TM3). In contrast, the time-limiting conformational changes involve reaching states of the intermediate region I that require the remodeling of the intra-helical H-bonding network at the intracellular end of TM6. Interestingly, these changes are specific to morphine-bound MOR and absent in the TRV-130-bound receptor, for which no macrostates with a bent TM6 intracellular end were observed. Finally, it is also worth mentioning that the timescales derived from simulations are much faster than those inferred from intramolecular FRET studies of prototypic GPCRs^36, 37^ (i.e., tens of milliseconds for small-molecule agonist-induced receptor activation), suggesting that the receptor conformational changes that precede interactions with intracellular partners may not correspond to the slowest degrees of freedom in receptor activation, but other kinetic bottlenecks (e.g., the ligand acquiring a bioactive conformation along the pathway) must underlie the rate-limiting steps in receptor activation.

## Methods

### System setup and adaptive sampling simulations

The nanobody-bound active crystal structure of MOR was simulated with its N-terminal region truncated at residue S64, in the absence of the nanobody, and with morphine or TRV-130 bound at the orthosteric binding site. Specifically, the MOR active crystal structure supplied by the Kobilka lab prior to its publication was used for the simulations reported herein. Virtually indistinguishable from the deposited PDB entry 5C1M^15^, this structure corresponds to a receptor construct with M264A and L265M mutations in ICL3. While morphine was docked manually to this structure using the position and orientation of the co-crystallized morphinan derivative BU72 as a reference, the starting TRV-130-bound MOR active structure was taken from our recently published microsecond-scale MD simulations of TRV-130 binding to MOR and its resulting most stable complex^33^.

The ligand-bound MOR complexes were each simulated in a pre-equilibrated 80 × 80 × 120 Å POPC/10% cholesterol bilayer solvated by TIP3P water molecules, as well as Na^+^, and Cl^−^ ions to neutralize the system’s overall charge and to achieve a physiological ionic strength. The final ligand-bound systems contained approximately 65,000 atoms. The CHARMM36 force field^38^ was used to simulate protein and lipids, while initial parameters for the ligands were obtained from the CHARMM General force field^39^ using the ParamChem webserver. Ligand parameters were verified and, when necessary, optimized, following published protocols^39^. Energy minimization was followed by preliminary equilibrations of the solvent with positional restraints on the membrane, the protein, and the ligand (1 ns in the NVT ensemble followed by 1 ns in the NPT ensemble). Restraints were gradually released over 3 ns from the lipids first, and then from the protein, followed by an additional 20 ns of unrestrained NPT equilibration. System temperature and pressure were maintained at 300 K and 1 bar, respectively, using the velocity rescale thermostat^40^ for temperature coupling, and the Parrinello-Rahman barostat^41^ for pressure coupling. A 12 Å cutoff was applied to nonbonded interactions using the Verlet scheme while setting a force switch at 10 Å for the van der Waals modifier. All equilibration runs were carried out with Gromacs 5.0.6^42^.

Production MD runs were carried out using the ACEMD software^43^ on a GPUGRID distributed network^44^. Two different sets of simulations using the adaptive sampling protocol implemented in the high-throughput molecular dynamics (HTMD) platform^23^ were performed. The first set of ~1500 simulations × 2 independent batches × 40 ns, for a total of ~120 µs for each ligand-receptor system, used as exploration metrics (a) the intramolecular contacts of the protein Cα atoms and (b) the inter-molecular contacts between the ligand heavy atoms and the protein Cα atoms. A cut-off of 8 Å was used to define these contacts, and only one every three Cα atoms between residues S64 and I352 were used to reduce computational cost. The second set of ~1500 simulations × 2 independent batches × 40 ns per ligand-receptor system, for an additional total of ~120 µs per ligand, used as exploration metrics (a) the distance between residues R165^3.50^ and T279^6.34^ and (b) the Cα RMSD of the NPxxYA segment (residues N332^7.49^ to A337^7.54^) from its corresponding conformation in the inactive crystal structure of MOR (PDB entry 4DKL)^14^. Overall, a total of 5981 and 5588 trajectories were generated for the morphine-bound and TRV-130-bound systems, respectively, for a total MD simulation time of approximately half millisecond.

### Simulation Analysis

Simulation trajectories were analyzed using the HTMD platform^23^, which provides a framework for efficiently handling a large number of simulations from multiple systems. HTMD functionalities were used to project combined trajectories onto a number of order parameters defined in the Results section (e.g. RMSD, distances between selected atoms, and sidechain dihedrals), as well as to perform dimensionality reduction, MSM generation, and transition path analysis between metastable states (see details below).

### Dimensionality reduction

Reliable kinetic characterization of a macromolecular system using MSMs strongly depends on the ability to finely discretize the phase space along slow order parameters. A recent application of the time-lagged independent component analysis **(**tICA) method was shown to successfully identify the slow-subspace of a high-dimensional phase space, and to provide the ideal order parameters for constructing MSMs^45^. Briefly, tICA combines information from the covariance matrix (**C**) and a time-lagged covariance matrix (**C** ^**tl**^) defined as:

1 $${\bf{C}}=\langle {\boldsymbol{x}}({\boldsymbol{t}}){{\boldsymbol{x}}}^{{\boldsymbol{T}}}({\boldsymbol{t}})\rangle $$

2 $${{\bf{C}}}^{{\boldsymbol{tl}}}(\tau )=\langle {\boldsymbol{x}}(t){{\boldsymbol{x}}}^{{\boldsymbol{T}}}({\boldsymbol{t}}+{\boldsymbol{\tau }})\rangle $$

where x(t) is the high-dimensional data vector at time t, τ is the lag time, and $\langle \ldots \rangle$ denotes time-average. The tICA components are then identified by solving the generalized eigenvalue problem:

3 $${{\bf{C}}}^{{\boldsymbol{tl}}}(\tau ){\bf{U}}={\bf{C}}{\bf{U}}{\boldsymbol{\Lambda }}$$

where **U** and **Λ** are the eigenvector and eigenvalue matrices respectively, so that the slowest reaction coordinates are the eigenvectors corresponding to the largest eigenvalues. Here, we obtain the slowest tICA reaction coordinates using interactions that describe protein conformations as well as protein-ligand interactions.

The protein conformations were described combining two sets of structural descriptors: (1) 4,656 pairs of Cα-Cα distances for 97 uniformly spaced residues (one every three to reduce computational cost) along the protein sequence, and (2) 2,396 sidechain-sidechain contact maps (using a cutoff of 7 Å on the minimum distance between sidechain heavy atoms) for all pairs that had a contact in either the active or inactive MOR crystal structures. Additionally, protein-ligand contacts were described using 289 residue-ligand pair contact maps, with contacts between the ligand and each of the MOR residues defined using a cutoff of 7 Å on the minimum distance between residue and ligand heavy atoms. Overall, a total of 7,341 dimensions per trajectory frame were used to obtain the slowest tICA reaction coordinates. Inspection of the marginal probabilities for the projection of the trajectories on the tICA eigenvector basis revealed that, for each of the two-ligand bound MOR systems, the distributions were qualitatively normal for components after the first 10. We therefore projected onto the first 10 tICAs to achieve dimensionality reduction in the definition of the microstates for Markov analysis. The slowest, and therefore most important, conformational degrees of freedom accessible to MOR were identified as those with maximal correlation in trajectory frames projected onto the 10 dominant tICA coordinates.

### Markov State Model analysis and transition pathways of MOR activation

MSMs provide a powerful method for integrating simulation data from several MD trajectories of varying length into a single stochastic model from which important information such as metastable states, thermodynamics, and kinetics of the system can be derived^46–49^. The system’s thermodynamic and kinetic information can be recovered from the eigenvectors and eigenvalues of the MSM transition matrix T(τ), which was obtained from the normalized counts of transitions occurring in a lag-time τ between a set of states (microstates), using maximum likelihood estimation under the constraint of detailed-balance. In this work, these states were obtained by finely discretizing the 10-dimensional conformational space defined by the dominant tICA coordinates into 1000 microstates, using the k-means clustering algorithm. Increasing or decreasing the value of this parameter by ~10% yields similar results. The relaxation timescales of the ligand-bound MOR systems are calculated as:

4 $${{\bf{t}}}_{{\bf{i}}}({\boldsymbol{\tau }}^{\prime} )=-\frac{{\boldsymbol{\tau }}^{\prime} }{{\bf{l}}{\bf{n}}|{{\boldsymbol{\lambda }}}_{{\boldsymbol{i}}}({\boldsymbol{\tau }}^{\prime} )|}$$

where $\tau ^{\prime}$ is the lag-time used for constructing the transition matrix, λ_i_ is the *i*-th eigenvalue of the transition probability matrix T(τ’), and t_i_ is the “implied” timescale corresponding to the *i*-th relaxation mode of the system. To ensure that the Markov transition matrix accurately describes the dynamics of the system (i.e., memory-less transitions between states), the lag-time τ’ was chosen by inspecting the convergence of the implied timescales of the model over increasing lag-times τ. Here, the implied relaxation times scales converge at a lag time of ~10 ns, suggesting that the dynamics of the system is Markovian for time resolutions equal or longer than such value. The eigenvectors corresponding to a particular eigenvalue (λ_i_ for i > 1) provide information on structural changes occurring at a specific timescale, while the eigenvector corresponding to the largest eigenvalue (i.e. λ_1_ = 1) represents the equilibrium probability of each of the microstates and were used to calculate the corresponding free-energies.

The metastable states of the system were identified by lumping these microstates together based on their kinetic similarity using spectral clustering of the transition matrix T(τ’) with the robust Perron Cluster Analysis (PCCA+) algorithm^50^, and the equilibrium probability of a macrostate was obtained by summing the equilibrium probability of all microstates assigned to that macrostate. The number of macrostates was chosen such that the intra-state fluctuations for the TM6 outward motion relative to TM3 and the backbone distortions involving the NPxxYA region at the TM7 cytoplasmic end would stay within a few angstroms.

The consistency between the dynamics described by the MSM and the actual MD simulation data was assessed with the Chapman-Kolmogorov test. Specifically, for increasing multiples of the MSM lag time, the probability of remaining in a given macrostate calculated directly from the MD trajectories was compared to that calculated from the MSM transition matrix.

A macrostate was deemed to belong to the inactive region of the conformational space if the MD conformations comprising a macrostate had an average Cα atom distance between residues R165^3.50^ and T279^6.34^ within 10 Å and the average Cα atom RMSD of the NPxxYA region (residues N332^7.49^ to A337^7.54^) from its conformation in the inactive crystal structure was within 2.5 Å. The macrostate properties such as averages of different order parameters reported in the Results section were calculated as weighted average of their microstates, where the weights are the equilibrium probabilities of the microstates. Kinetic evolution of macrostate probabilities was calculated by starting from an initial probability proportional to the equilibrium macrostate probability in the inactive macrostate and zero elsewhere, calculating the evolution by sequential applications of the transition probability matrix, and finally projecting the microstate probabilities onto each macrostate. The most probable pathways between the inactive and active metastable states of MOR were obtained using the Transition Path Theory^51^ implementation in PyEMMA^52^ accessed through HTMD^23^.

### Transfer entropy analysis

To investigate the information flow in the two ligand-bound trajectories, we employed the information theory measure of transfer entropy^53^ which captures the causality of correlated motion. The dynamics of residues was described using each pair of receptor residues and residue-ligand pairs using 2,741 residue-residue contact pairs (determined with a cutoff of 7 Å on the minimum distance between heavy atoms in either the active or inactive MOR crystal structures) and 289 residue-ligand contacts. Thus, a total of 3,030 dimensions per trajectory frame were used to obtain the transfer entropy matrix. Specifically, the transfer entropy between residue J and residue I was defined as

5 $${T}_{J\to I}=\sum _{t}p({x}_{t+1}^{(I)},{x}_{t}^{(I)},{x}_{t}^{(J)})\,\mathrm{log}\,\frac{p({x}_{t+1}^{(I)}|{x}_{t}^{(I)},{x}_{t}^{(J)})}{p({x}_{t+1}^{(I)}|{x}_{t}^{(I)})}$$

6 $$=H({x}_{t+1}^{(I)}|{x}_{t}^{(I)})+H({x}_{t+1}^{(I)},{x}_{t}^{(I)})-H({x}_{t+1}^{(I)},{x}_{t}^{(I)},{x}_{t}^{(J)})$$

where *x* ^(I)^ is the vector of contacts formed by residue I. In this equation, H is the entropy associated to a given random variable, defined as H(x) = ∫ p(x) log p(x) dx. The values of the transfer entropy matrix were corrected using shuffled trajectories for residue J as:

7 $${\tilde{T}}_{J\to I}={T}_{J\to I}-\,{{T}^{(S)}}_{J\to I}$$

where the T^(S)^ is the value of the transfer entropy calculated after a random permutation of the time indices for x^(J)^ in equation (6).

To reduce the computational cost, the transfer entropy calculation was performed on a randomly selected set of 30% of the trajectories. For each ligand-bound MOR system, 3 independent transfer entropy calculations were performed and the average of the three runs was reported as the final transfer entropy matrix in the results section. For each ligand-bound MOR system, the resulting transfer entropy matrix was used to calculate a directed graph, where each node in the graph represented a residue as well as the ligand, and the directed edge between two nodes indicated the transfer entropy between them. Link analysis was then performed on the computed graph using the HITS algorithm^54^, which ranks each node based on two values: the authority score, which reflects the node value based on the incoming links (i.e. estimates a residue value as a receiver), and the hub score, which reflects the node value based on outgoing links (i.e. estimates a residue value as a transmitter). Transfer entropy was calculated using the MDEntropy package, while graph calculations were performed with the NetworkX python library.

### Structural interaction fingerprint

For each MOR residue, ligand-receptor interaction fingerprints were encoded in a binary representation using 7 bits per residue, corresponding to seven types of interactions (specifically: apolar (van der Waals), aromatic face to face, aromatic edge to face, hydrogen bond with protein residues as hydrogen bond donors, hydrogen bond with protein residues as hydrogen bond acceptors, electrostatic interaction with positively charged protein residues, and electrostatic interaction with negatively charged protein residues). A distance cutoff of 4.5 Å was used to identify apolar interactions between two non-polar atoms, while a cutoff of 4 Å was used to identify aromatic and electrostatic interactions. Ligand-residue interactions reported here refer to interactions with residue side chain only, and were calculated using an in-house python script.

### Error estimation

Errors in MSM estimations (e.g., steady-state probabilities and mean-first passage times) were obtained by bootstrapping. A total of 500 bootstrap samples, each consisting of 95% of randomly selected trajectories including all frames, were generated and the first and the third quartile of the 500 estimates were used to calculate error bars.

### Data availability

The data that support the findings of this study are available from the corresponding author upon request.

## Electronic supplementary material

Supplementary Information
