# Supplementary material for: Dynamic and Kinetic Elements of µ-Opioid Receptor Functional Selectivity
Source: Sci Rep. 2017 Sep 12;7:11255. doi: 10.1038/s41598-017-11483-8 (PMC5595830; doi:10.1038/s41598-017-11483-8)
Supplement: Supplementary file 1 — Supplementary Information [file 41598_2017_11483_MOESM1_ESM.doc]

**SUPPLEMENTARY INFORMATION**

**Dynamic and Kinetic Elements of µ-Opioid Receptor Functional Selectivity**

Abhijeet Kapoor,^a,§^ Gerard Martinez-Rosell,^b,§^ Davide Provasi,^a,§^ Gianni de Fabritiis,^b,*^ and Marta Filizola^a,*^

*^a^Department of Pharmacological Sciences, Icahn School of Medicine at Mount Sinai, New York, NY, USA.*

*^b^Computational Biophysics Laboratory (GRIB-IMIM), Universitat Pompeu Fabra, Barcelona Biomedical Research Park (PRBB), Barcelona, Spain.*

Supplementary Fig. 1-16……………………………………………………...………pg. 2-16

Supplementary Table 1-15……………………………………………………………pg. 17-43

**Supplementary Results**


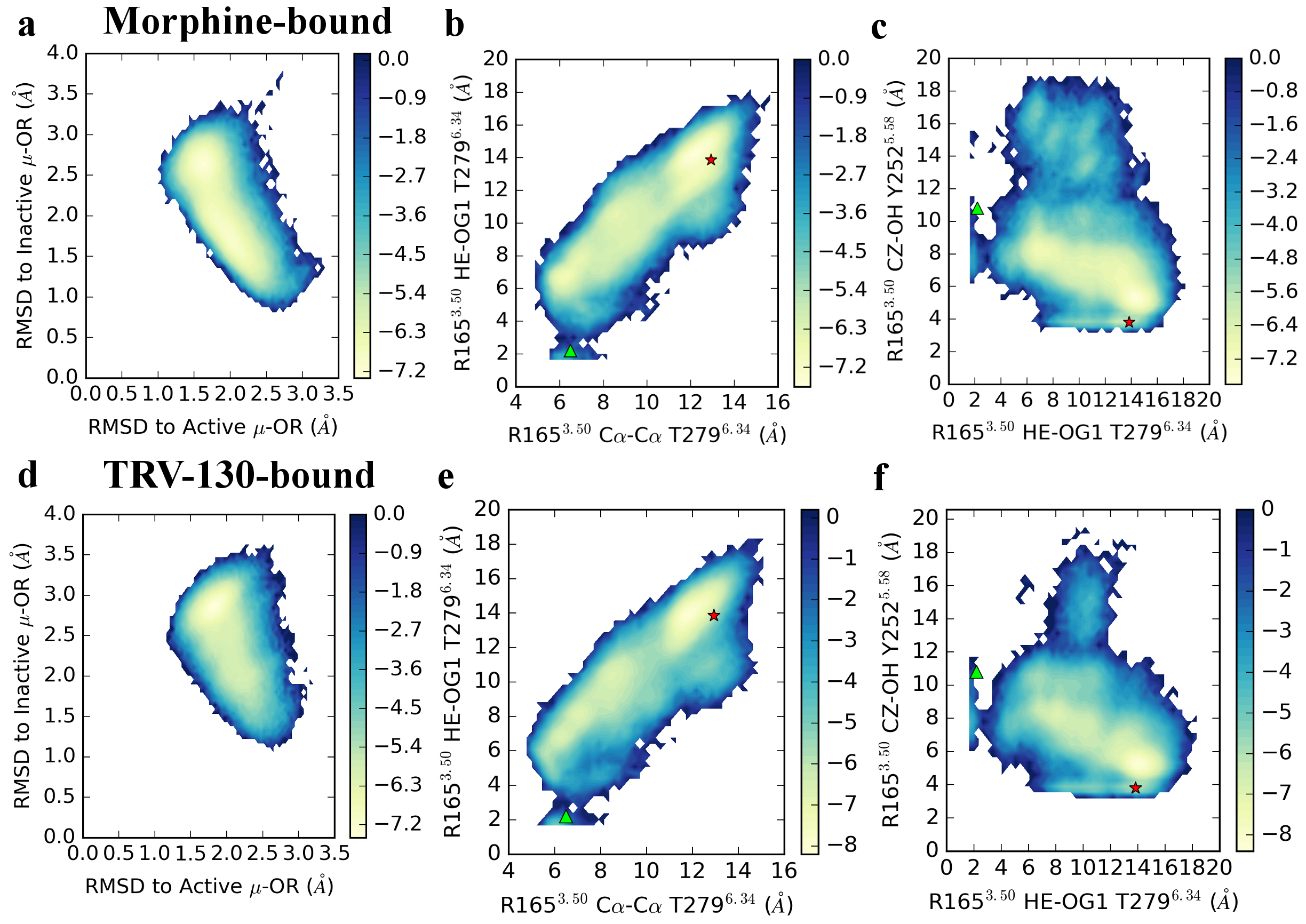


**Supplementary** **Figure 1.** Projection of morphine-bound (a-c) and TRV-130-bound (d-f) trajectory frames onto order parameters measuring: **a, d.** C_α_-atom RMSD of transmembrane helices relative to inactive (PDB ID: 4DKL) and active (PDB ID: 5C1M) crystal structures of µ-OR. **b, e.** Distance between C_α_-atoms and sidechain atoms involved in hydrogen bonds between residues R165^3.50^ and T279^6.34^ (a measure of TM6 outward movement relative to TM3). **c, f.** Distance between sidechain atoms of residues R165^3.50^ and T279^6.34^, and R165^3.50^ and Y252^5.58^ (a measure of TM5 inward movement). Corresponding distances in active (PDB ID: 5C1M) and inactive (PDB ID: 4DKL) crystal structures are shown as red stars and green triangles, respectively.


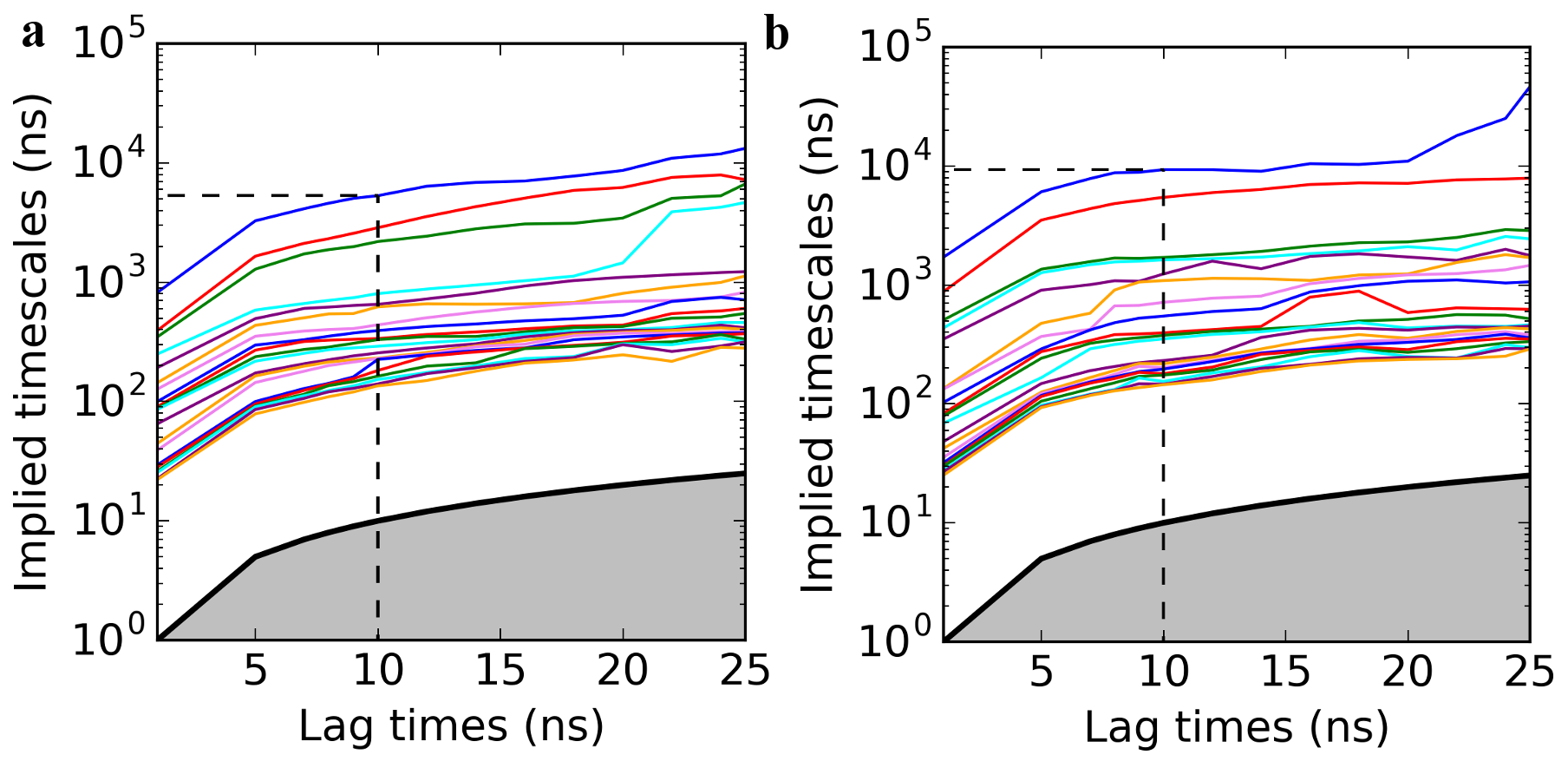


**Supplementary Figure 2.** Top 20 implied timescales as a function of the lag time, derived from the transition probability matrix with 1000 microstates for the **(a)** morphine-bound MOR and **(b)** TRV-130-bound MOR systems. The Markov state model constructed at a lag time of 10 ns was used for subsequent analyses. Dotted lines indicate the slowest relaxation timescale for each system as predicted by a model with a 10 ns lag time. The gray area indicates the region of relaxation faster than the lag time, which cannot be properly described by the model.


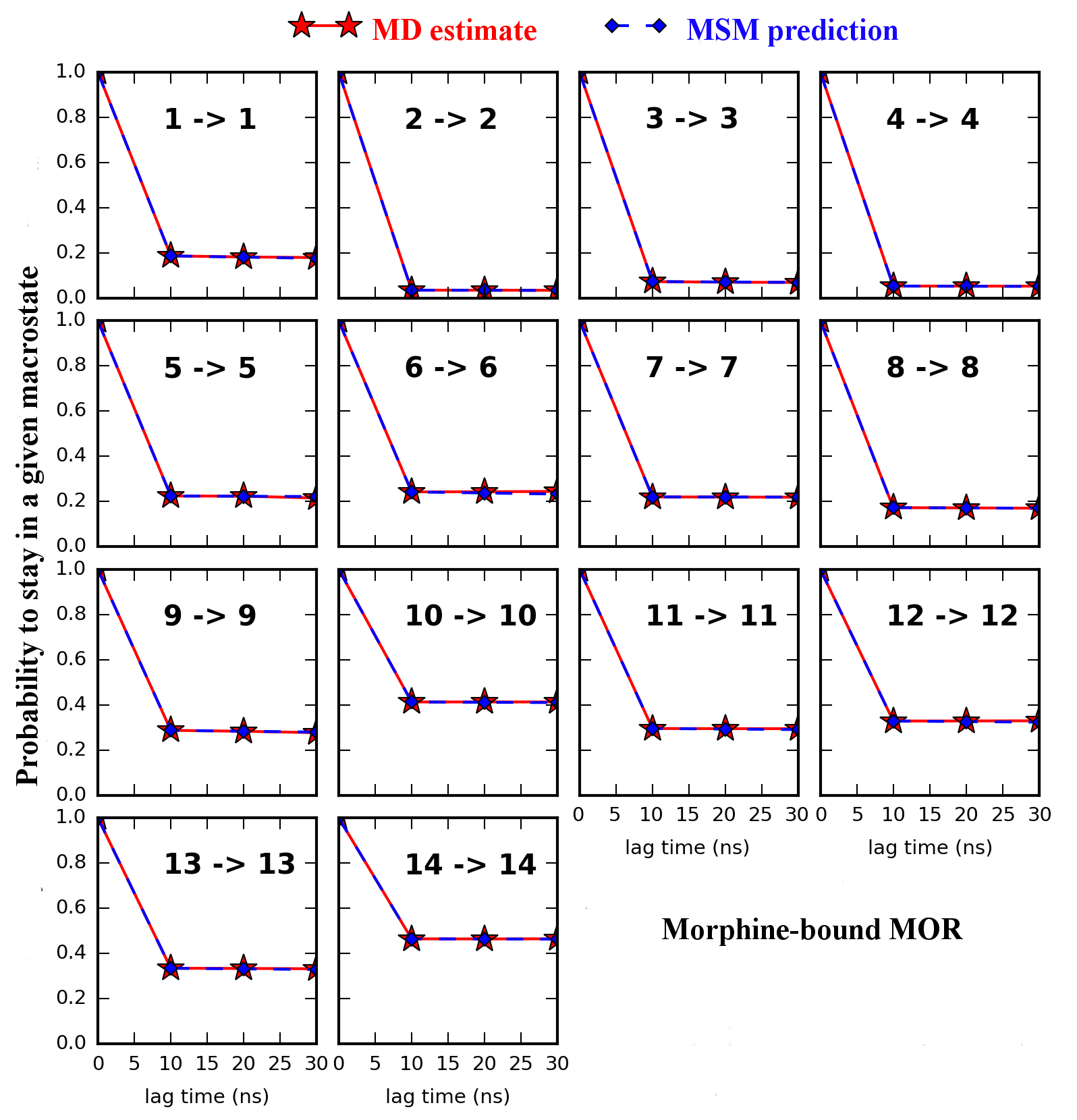


**Supplementary Figure 3.** Chapman-Kolmogorov test of the Markov state model generated at lag time of 10 ns for the morphine-bound MOR system. For each of the identified 14 kinetic macrostates (metastable states), the probabilities to remain in that macrostate (indicated as 1→1, 2→2, and so on) calculated from the Markov model transition matrix (dotted blue lines with diamond markers) are nearly identical to those derived directly from MD simulation data (red line with star markers), for up to three lag times (i.e., 10 ns, 20 ns, and 30 ns, respectively).

**
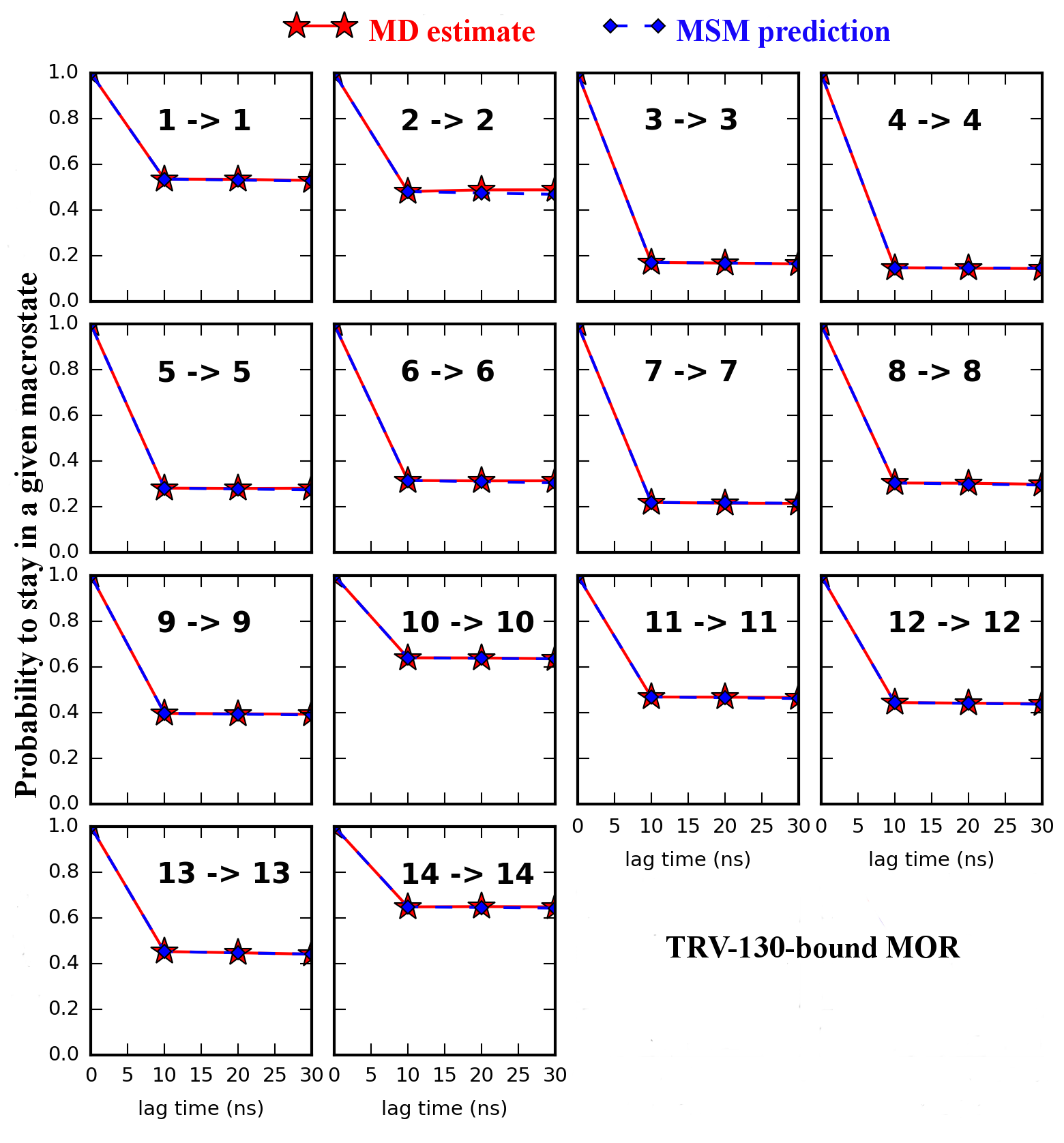
**

**Supplementary Figure 4.** Chapman-Kolmogorov test of the Markov state model generated at lag time of 10 ns for the TRV-130-bound MOR system. For each of the identified 14 kinetic macrostates (metastable states), the probabilities to remain in that macrostate (indicated as 1→1, 2→2, and so on) calculated from the Markov model transition matrix (dotted blue lines with diamond markers) are nearly identical to those derived directly from MD simulation data (red line with star markers), for up to three lag times (i.e., 10 ns, 20 ns, and 30 ns, respectively).


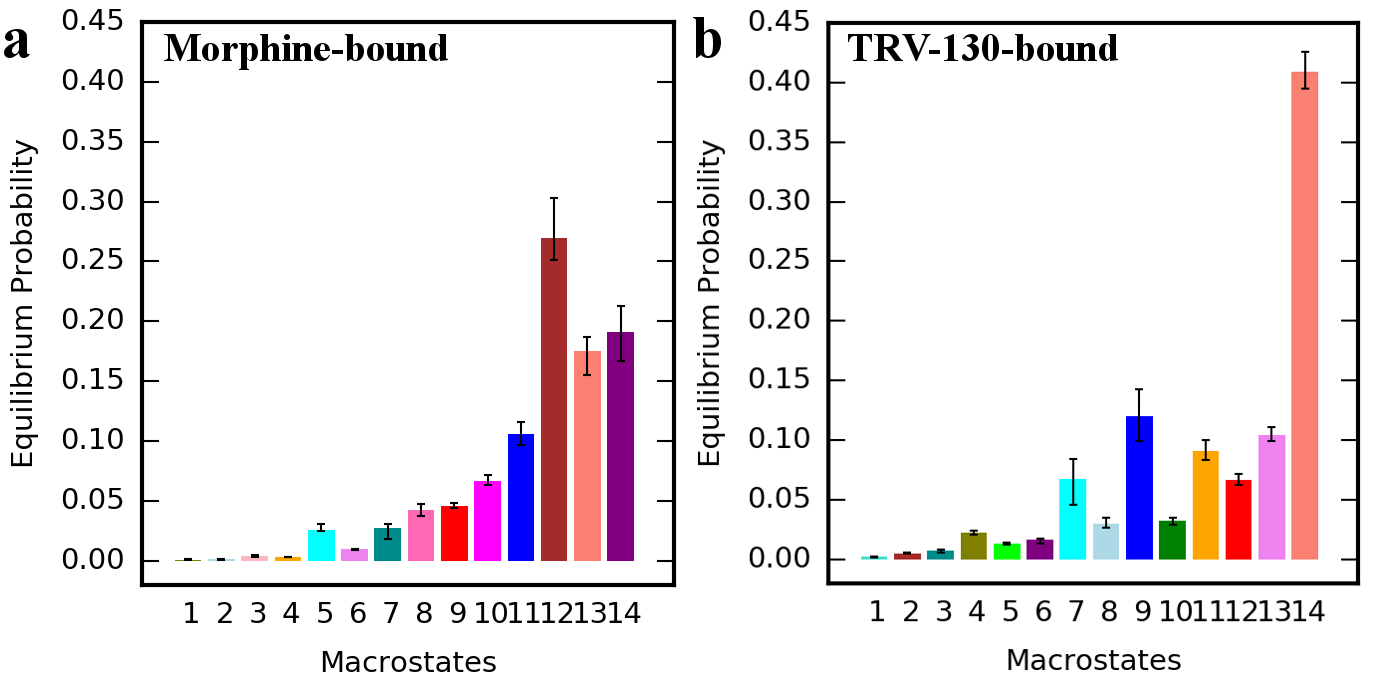


**Supplementary Figure 5.** Equilibrium probabilities of the identified 14 kinetic macrostates (metastable states) for the **(a)** morphine-bound MOR, and **(b)** TRV-130-bound MOR systems. The lower and upper bounds of the error bars represent the first and third quartiles, respectively, of the 500 bootstrap samples.


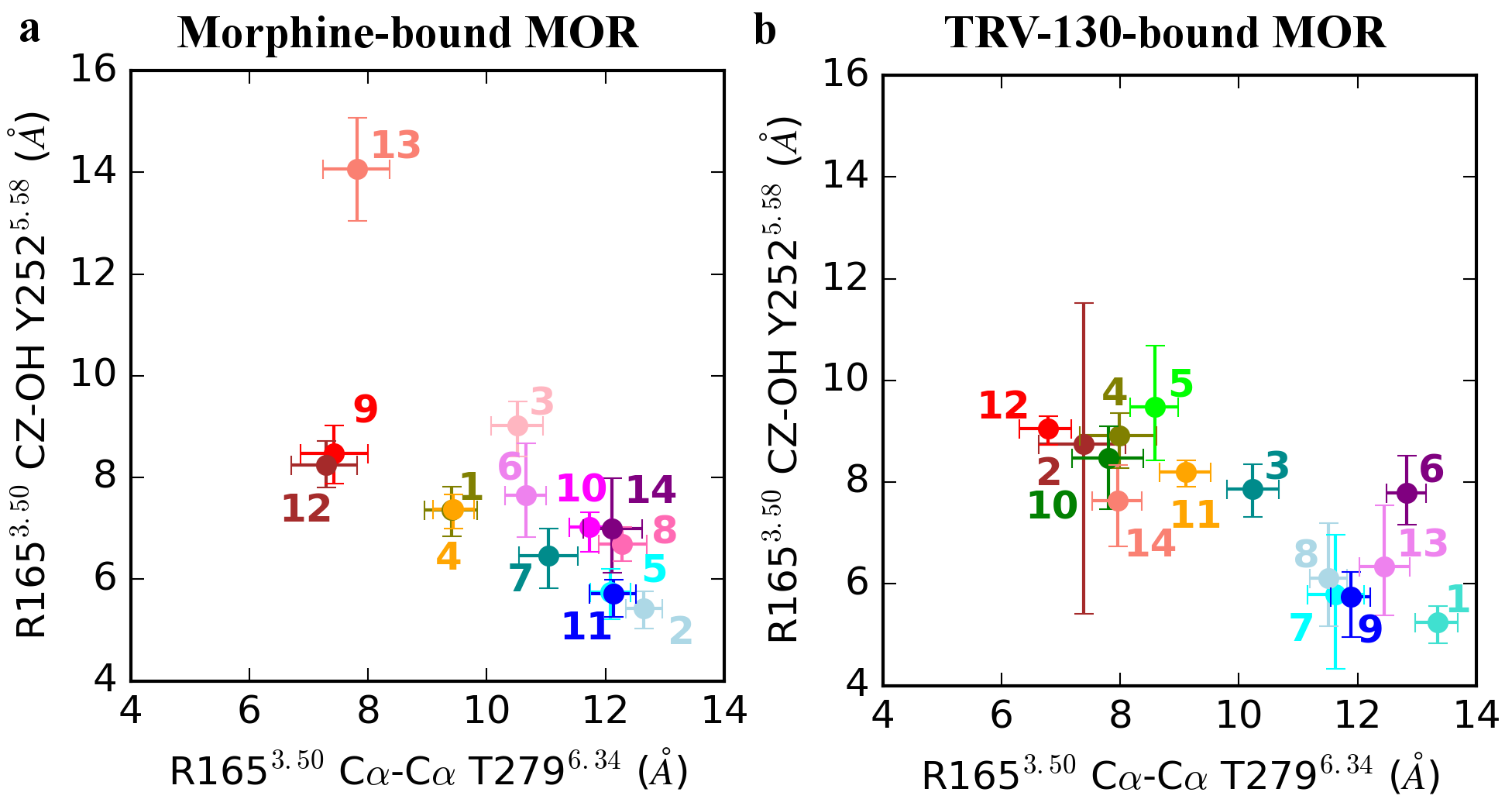


**Supplementary Figure 6.** Microstate-based reweighted averages of the TM3-TM6 distance (x-axis) and TM3-TM5 distance (y-axis) for the identified 14 kinetic macrostates of the **(a)** morphine-bound MOR and **(b)** TRV-130-bound MOR systems. Specifically, these distances correspond to the R165^3.50^ - T279^6.34^ Cα- Cα distance (x axis) and the Cζ-OH sidechain distance between residues R165^3.50^ – Y252^5.58^ (y axis). Error bars show the first quartile as the lower bound and the third quartile as the upper bound of the two order parameter values in each of the macrostates. Color codes for the macrostates are the same as in Figure 1 of the main text.


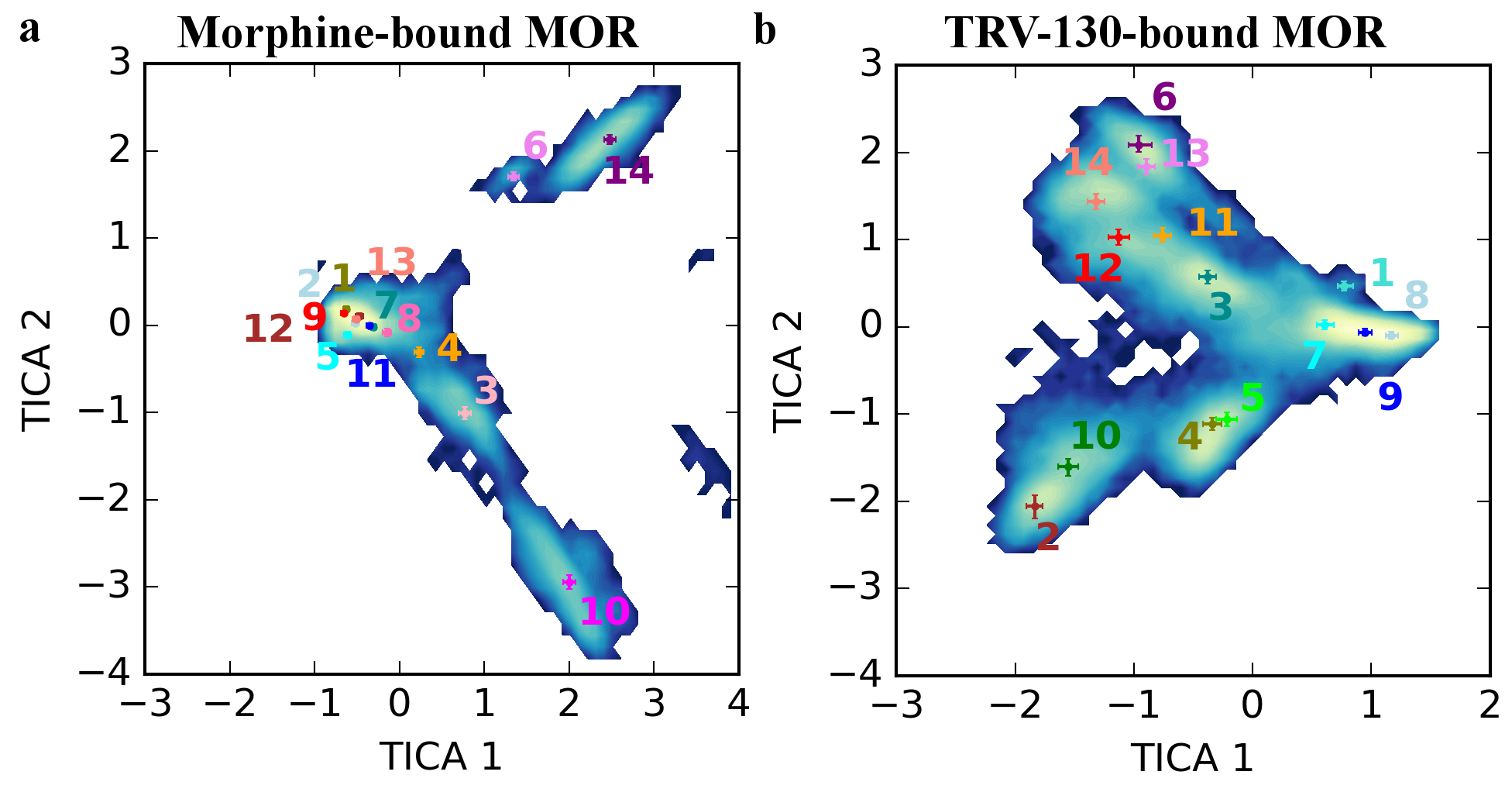


**Supplementary Figure 7.** Projections of morphine-bound (a) and TRV-130-bound (b) MOR trajectory frames onto their first two tICA components. The two-dimensional histograms represent the count density of the projected values, with increasing density values shown from blue to yellow. Dots represent averages (reweighted according to the MSM stationary probability) of the two projections in each of the 14 macrostates identified based on kinetic similarity. These macrostates are numbered as in Supplementary Figure 5 and are colored based on the region they fall within, as shown in Figure 1 of the main text. Vertical and horizontal bars refer to the first (lower bound) and third (upper bound) quartiles of the distributions of the two projections in each macrostate.

**
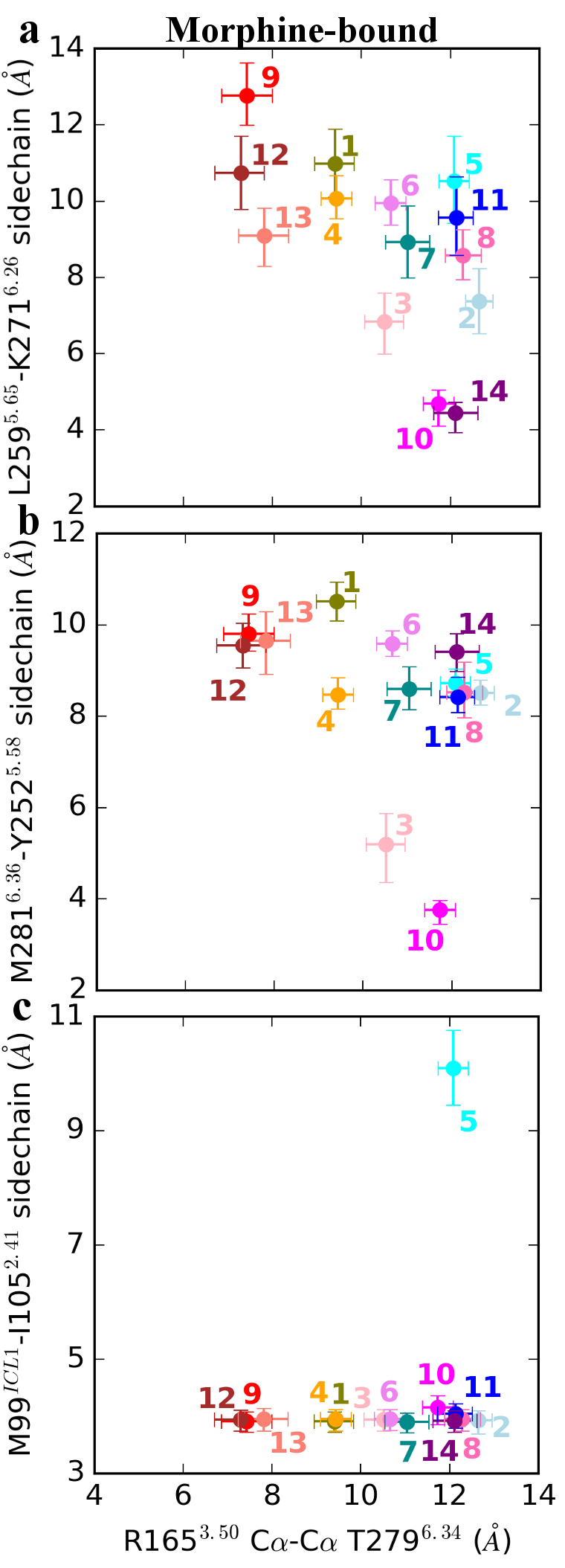
**

**Supplementary Figure 8.** Microstate-based reweighted averages of the TM3-TM6 distance (x-axis) vs. sidechain residue pair distances (y axis) with highest correlation according to tICAs 1 **(a)**, 2 **(b)**, and 3 **(c)** in the morphine-bound MOR system. Specifically, the TM3-TM6 distance is exemplified by the R165^3.50^ - T279^6.34^ Cα distance (x axis), whereas the y axis reports the minimum sidechain heavy atom distance between residue pairs **(a)** L259^5.65^-K271^6.26^, **(b)** M281^6.36^-Y252^5.58^, and **(c****)** M99^ICL1^-I105^2.41^. Macrostates are shown as dots and color coded as in Figure 1 of the main text. Error bars show the first and third quartiles of the order parameter values in each of the macrostates.


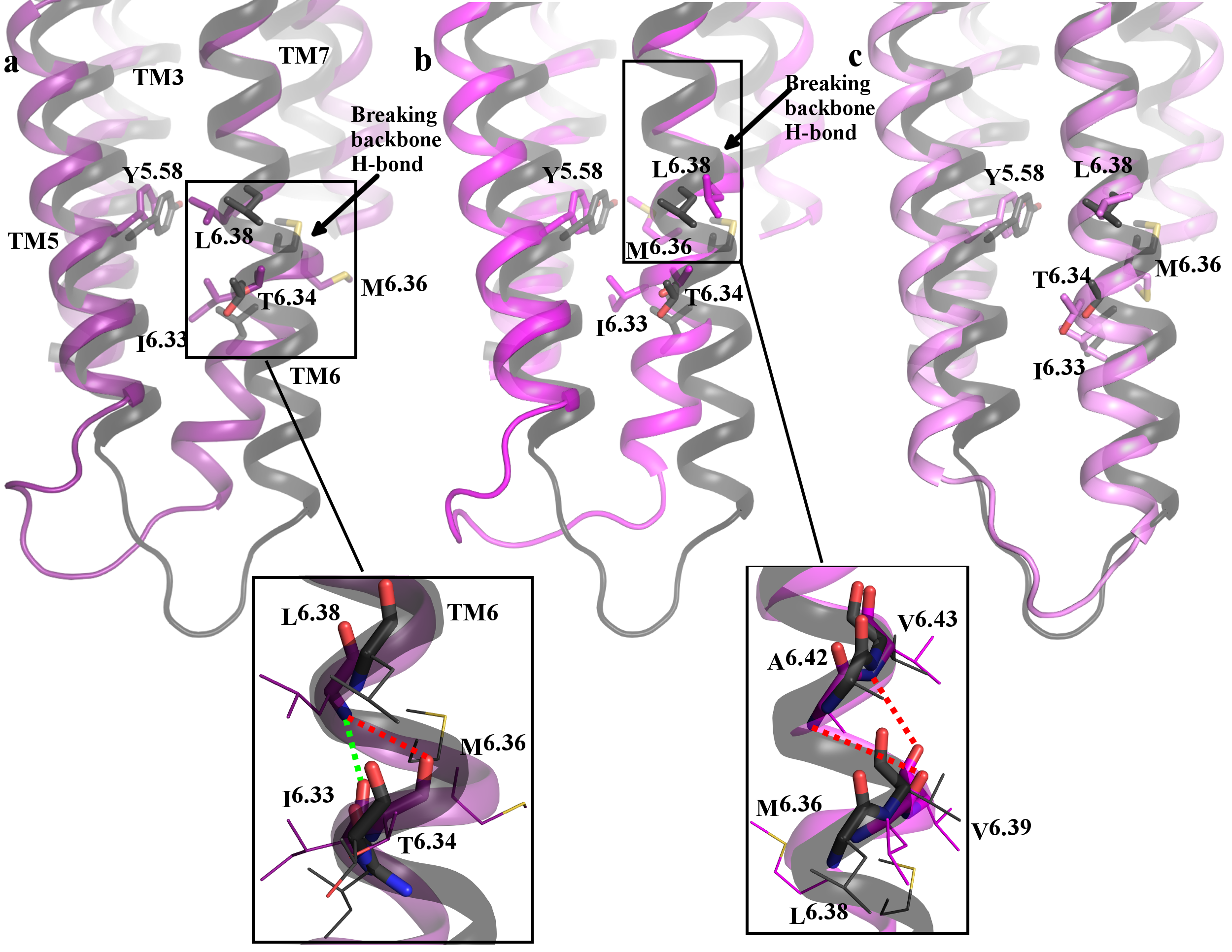


**Supplementary Figure 9. Vertical views of the activated crystal structure of MOR superimposed to highly probable kinetic macrostates of the intermediate region I of the morphine-bound or TRV-130-bound MOR systems.** The activated MOR crystal structure is shown in gray. Also shown are: **(a)** macrostate #14 of the morphine-bound MOR system, **(b)** macrostate #10 of the morphine-bound MOR system, and **(c)** macrostate #13 of the TRV-130-bound MOR. In panels a, b, the position of the backbone hydrogen bonds that either break or form as a result of TM6 bending is indicated with an arrow, and the residue sidechains that rearrange as a result of this bending are shown as sticks. Insets to panels a, b zoom in the forming hydrogen bonds between the backbone amide nitrogen and carbonyl oxygen atoms (dotted green line) as well as the breaking backbone hydrogen bonds (dotted red lines). In these insets, backbone atoms are shown as sticks and sidechain atoms are shown as lines. For clarity, TM1, TM2, and TM4 are not shown.


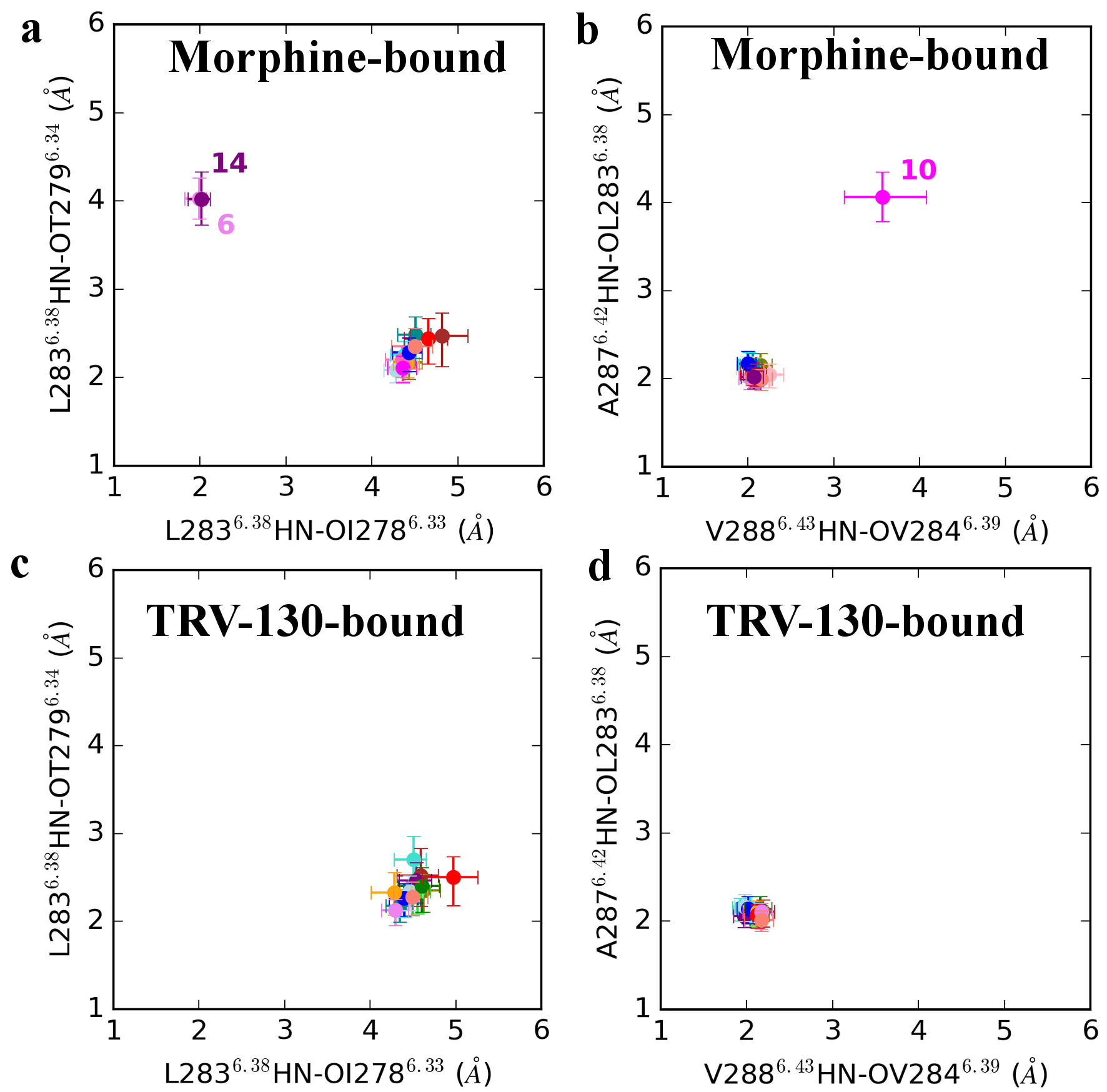


**Supplementary Figure 10.** Microstate-based reweighted averages of order parameters measuring backbone hydrogen bond distances between residues **(a,c)** T279^6.34^-L283^6.38^ vs. I278^6.33^-L283^6.38^, and **(b,d)** L283^6.38^-A287^6.42^ vs. V284^6.39^-V288^6.43^ for the identified 14 kinetic macrostates of morphine-bound or TRV-130-bound MOR systems. Reweighted average values of these order parameters are shown as dots. Error bars represent the first quartile as the lower bound and third quartile for the upper bound. Color codes for macrostates are as in Figure 1 of the main text.

**
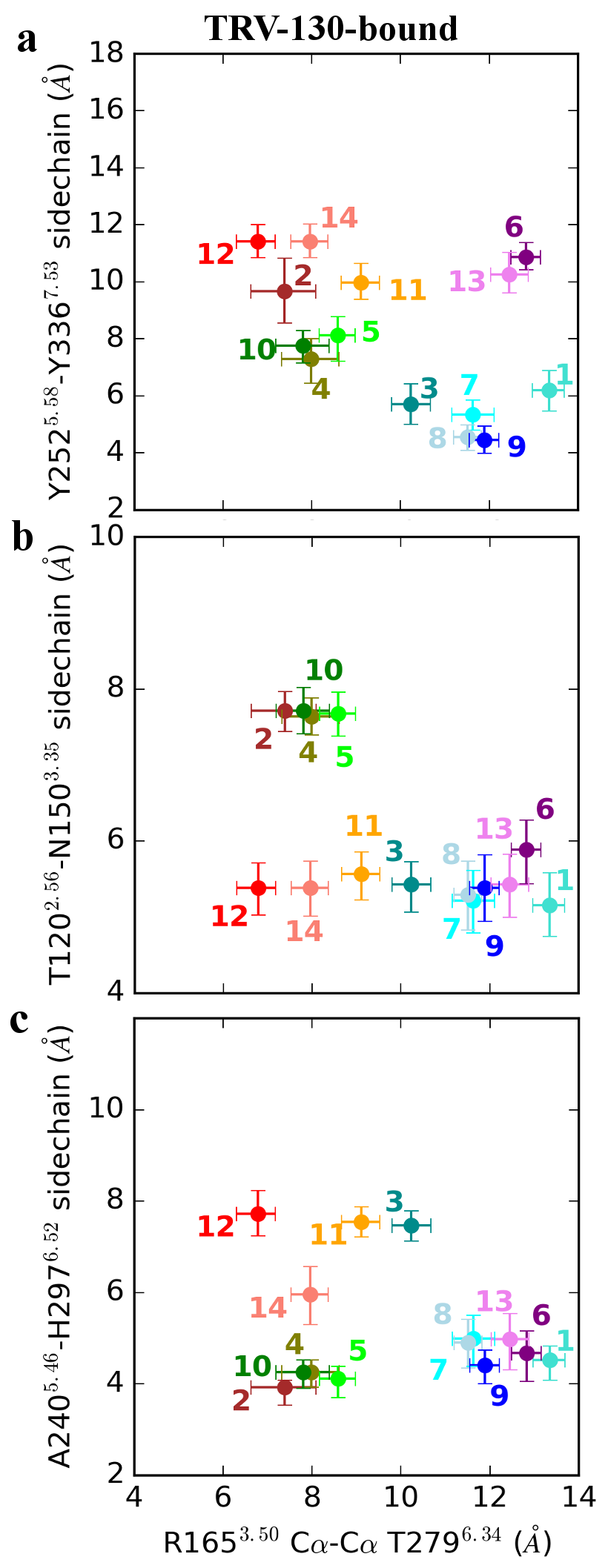
**

**Supplementary Figure 11.** Microstate-based reweighted averages of the TM3-TM6 distance (x-axis) vs. sidechain residue pair distances (y axis) with highest correlation according to tICAs 1 **(a)**, 2 **(b)**, and 3 **(c)** for the TRV-130-bound system (y axis). Specifically, the TM3-TM6 distance is exemplified by the R165^3.50^ - T279^6.34^ Cα distance (x axis), whereas the y axis reports the minimum sidechain heavy atom distance between residues **(a)** Y252^5.58^-Y336^7.53^ **(b)** T120^2.56^-N150^3.35^, and **(c)** A240^5.46^-H297^6.52^. Color codes for the macrostates are the same as in Figure 1 of the main text. Error bars show the first quartile as the lower bound and the third quartile as the upper bound of the order parameter values in each of the macrostates.

**
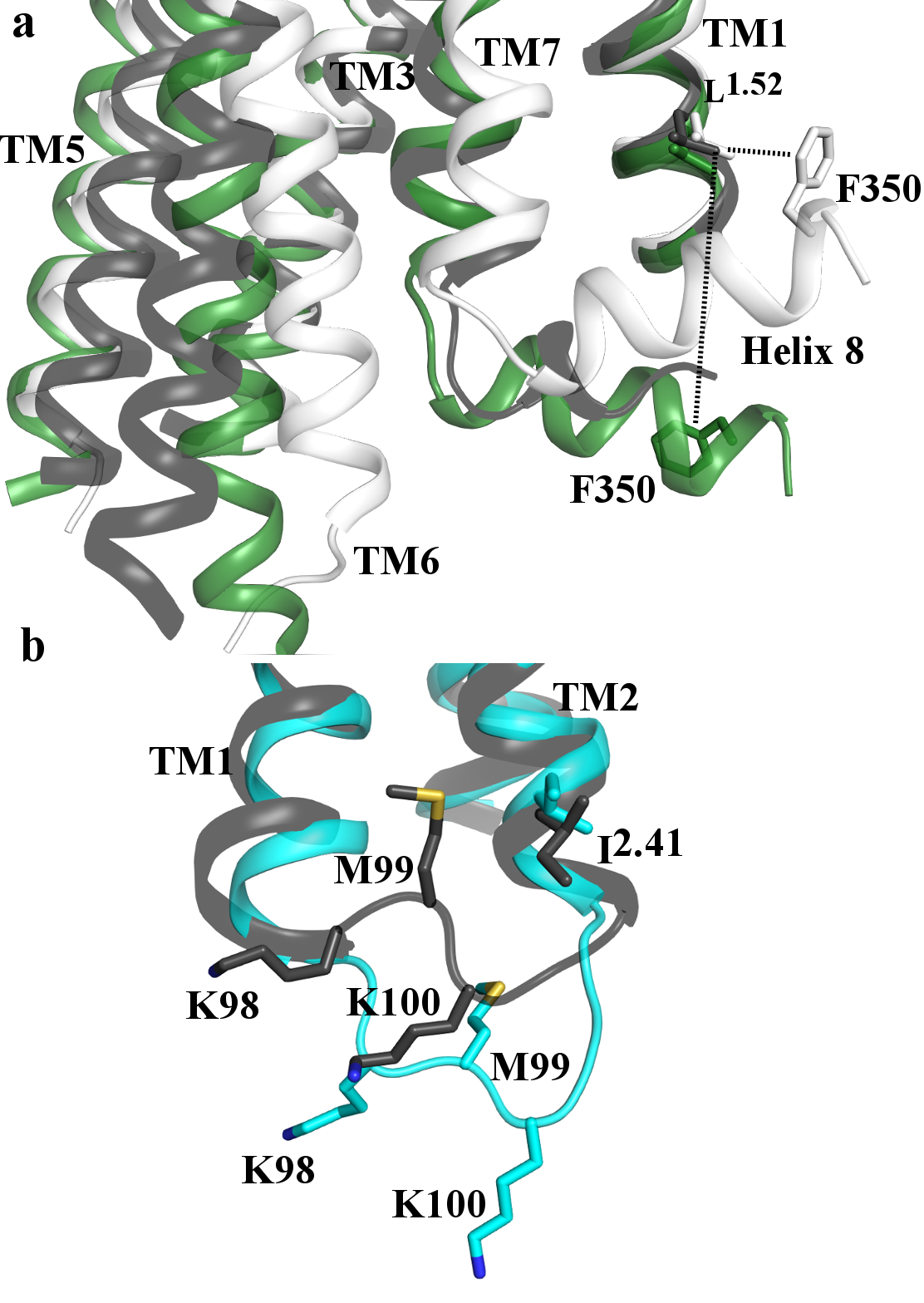
**

**Supplementary Figure 12. Conformational rearrangements of helix 8 and ICL1 domains during simulation**. **(a)** Vertical view of a representative conformation of the most probable macrostate of the intermediate region II of the TRV-130-bound system (macrostate #10, in green) overlapped onto the MOR active (gray) and inactive (white) crystal structures. Dotted lines indicate the distance between the sidechains of residues L88^1.52^ and F350 in the inactive MOR crystal structure compared to macrostate #10. TM2, TM4, and intracellular loops are not shown for clarity. **(b)** Vertical view of a representative conformation of macrostate 5 (cyan) of the morphine-bound system compared to the corresponding region in the MOR active crystal state (in gray). Only TM1, TM2, and ICL1 are shown for clarity.

**
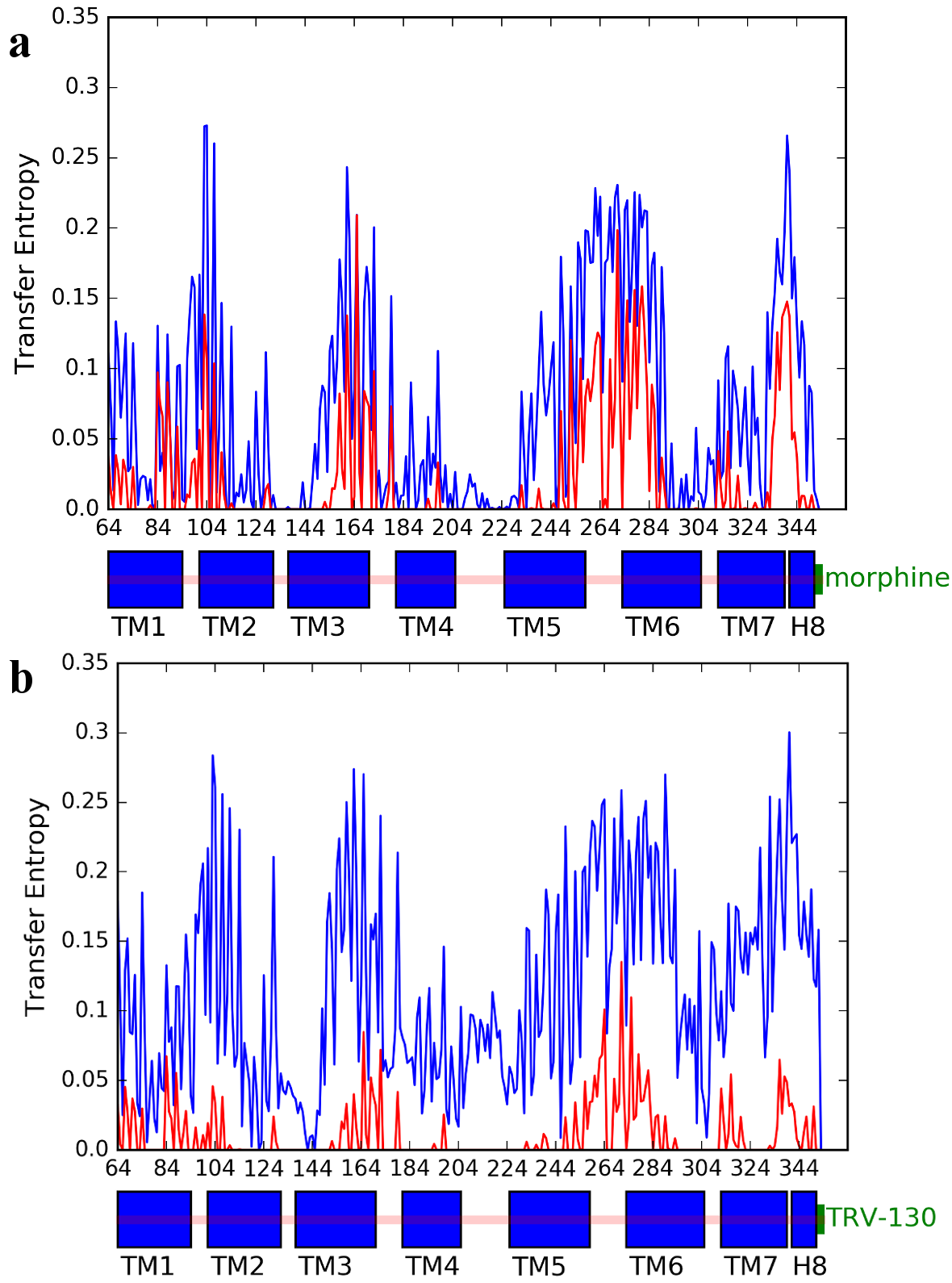
**

**Supplementary Figure 13. Information transfer between ligands and MOR residues in** **(a) morphine-bound MOR and (b) TRV-130-bound MOR**. Information transferred from the ligand to the receptor is in blue, whereas information received by the ligand from the receptor is in red.

**
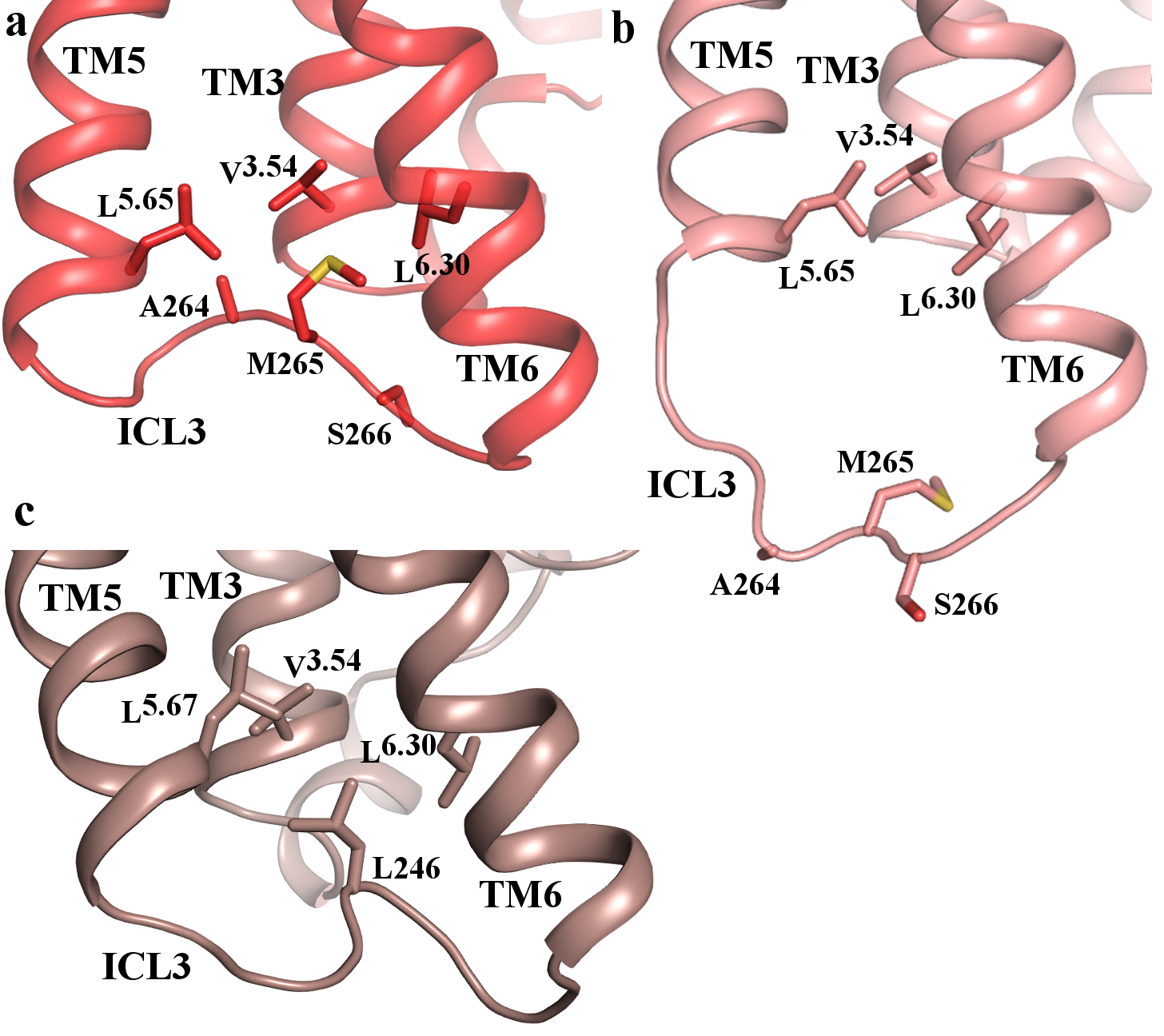
**

**Supplementary Figure 14. ICL3 conformations sampled during dynamics of the morphine-bound MOR system compared to the corresponding region in the inactive delta-opioid crystal structure**. Vertical views of the ICL3 domain of representative conformations of two inactive region macrostates of the morphine-bound MOR system, specifically: **(a)** macrostate #9, and **(b)** macrostate #12. (c) ICL3 conformation in the ultra-high resolution crystal structure of the inactive delta-opioid receptor (PDB id: 4N6H).

**
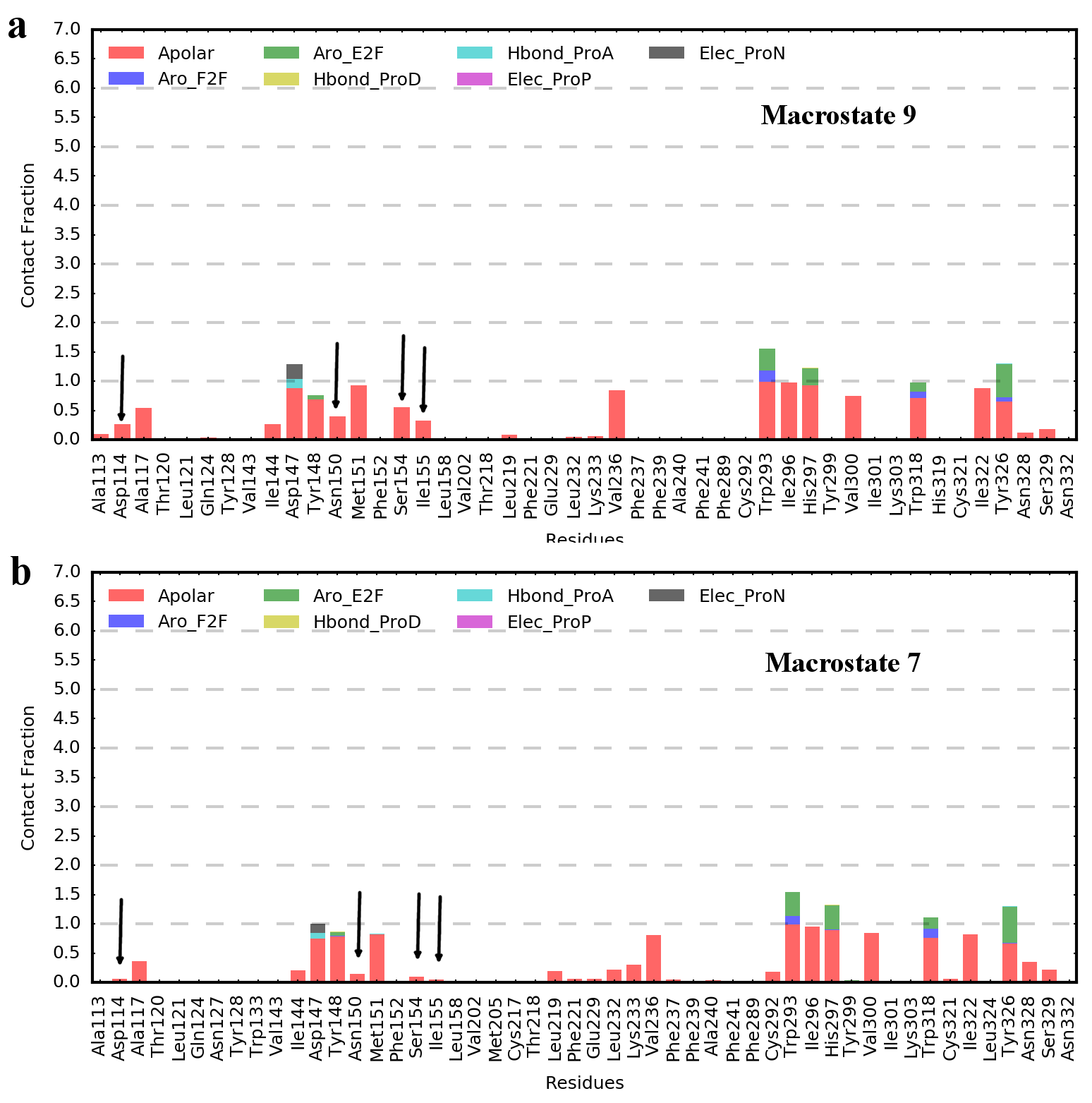
**

**Supplementary Figure 15.** TRV-130-MOR interaction fingerprints calculated using trajectory frames corresponding to (a) macrostate #9 and (b) macrostate #7 of the TRV-130-bound MOR system. For each MOR residue, seven interaction types are calculated: apolar, aromatic face-to-face (Aro_F2F, blue), aromatic edge-to-face (Aro_E2F, green), hydrogen bond with the protein as hydrogen bond donor (Hbond_ProD, yellow), hydrogen bond with the protein as hydrogen bond acceptor (Hbond_ProA, cyan), electrostatic interaction with the protein positively charged (Elec_ProP, magenta), and electrostatic interaction with the protein negatively charged (Elec_ProN, gray). The contact fraction on the y axis represents the probability of each of the 7 interaction types, and is calculated as the fraction of trajectory frames in which a given interaction is formed. For each interaction type, the contact fraction varies from 0 to 1. Ligand interactions reported here are calculated with residue side chain atoms only.

**
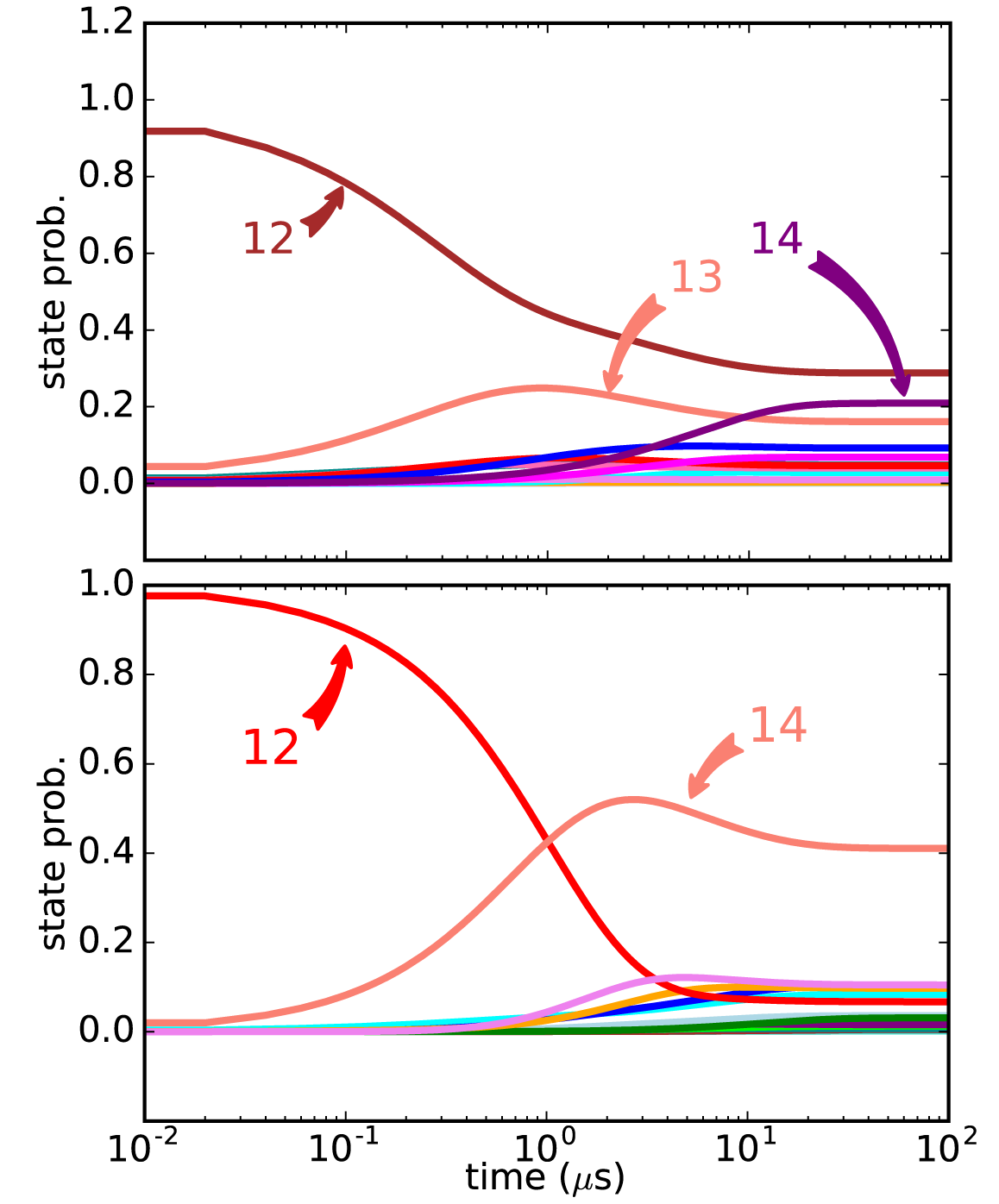
**

**Supplementary Figure 16.** Kinetic evolution of the probability of each macrostate when starting from the inactive morphine-bound (top panel) and TRV-130-bound (bottom) MOR systems (states #12 for both). Macrostates with probability >20% at any time are indicated by arrows.

**Table 1. Top 75 (~1%) structural descriptors of the slowest conformational degrees of freedom accessible to morphine-bound and TRV-130-bound MOR identified by tICA dimension 1. Structural descriptors involving correlated residue sidechains are highlighted in red. Commonalities, if any, between the morphine-bound MOR and TRV-130-bound MOR are highlighted in green.**

| **Morphine-bound MOR** | | **TRV-130-bound MOR** | |
| --- | --- | --- | --- |
| **Structural Descriptors** | **Correlation** | **Structural Descriptors** | **Correlation** |
| N274^6.29^_Cα--I298^6.53^_Cα | -0.83 | G82^1.46^_Cα--V250^5.56^_Cα | -0.85 |
| L259^5.65^--K271^6.26^ | 0.83 | S76^1.40^_Cα--V250^5.56^_Cα | -0.85 |
| K271^6.26^_Cα--I298^6.53^_Cα | -0.82 | G82^1.46^_Cα--G253^5.59^_Cα | -0.85 |
| N274^6.29^_Cα--V286^6.41^_Cα | -0.81 | S76^1.40^_Cα--G253^5.59^_Cα | -0.85 |
| N274^6.29^_Cα--I301^6.56^_Cα | -0.80 | G253^5.59^_Cα--L331^7.48^_Cα | -0.84 |
| V250^5.56^_Cα--K271^6.26^_Cα | -0.80 | C79^1.43^_Cα--G253^5.59^_Cα | -0.84 |
| V250^5.56^_Cα--N274^6.29^_Cα | -0.79 | C79^1.43^_Cα--V250^5.56^_Cα | -0.84 |
| K271^6.26^_Cα--P295^6.50^_Cα | -0.79 | G85^1.49^_Cα--G253^5.59^_Cα | -0.84 |
| N274^6.29^_Cα--A304^6.59^_Cα | -0.79 | G85^1.49^_Cα--V250^5.56^_Cα | -0.83 |
| I247^5.53^_Cα--N274^6.29^_Cα | -0.79 | G82^1.46^_Cα--I247^5.53^_Cα | -0.83 |
| N274^6.29^_Cα--P295^6.50^_Cα | -0.79 | G253^5.59^_Cα--N328^7.45^_Cα | -0.83 |
| R277^6.32^_Cα--V286^6.41^_Cα | -0.79 | A73^1.37^_Cα--V250^5.56^_Cα | -0.83 |
| K271^6.26^_Cα--I301^6.56^_Cα | -0.78 | G82^1.46^_Cα--I256^5.62^_Cα | -0.83 |
| G253^5.59^_Cα--K271^6.26^_Cα | -0.78 | S76^1.40^_Cα--I247^5.53^_Cα | -0.83 |
| I238^5.44^_Cα--N274^6.29^_Cα | -0.78 | I256^5.62^_Cα--L331^7.48^_Cα | -0.83 |
| K271^6.26^_Cα--A304^6.59^_Cα | -0.77 | A73^1.37^_Cα--G253^5.59^_Cα | -0.83 |
| L259^5.65^_Cα--K271^6.26^_Cα | -0.77 | V250^5.56^_Cα--N328^7.45^_Cα | -0.83 |
| K271^6.26^_Cα--T307^ECL3^_Cα | -0.77 | A73^1.37^_Cα--I247^5.53^_Cα | -0.83 |
| I256^5.62^_Cα--K271^6.26^_Cα | -0.76 | S76^1.40^_Cα--I256^5.62^_Cα | -0.83 |
| Y252^5.58^--I278^6.33^ | 0.76 | G85^1.49^_Cα--I256^5.62^_Cα | -0.82 |
| F241^5.47^_Cα--N274^6.29^_Cα | -0.75 | V250^5.56^_Cα--L331^7.48^_Cα | -0.82 |
| I278^6.33^--L283^6.38^ | 0.75 | C79^1.43^_Cα--I256^5.62^_Cα | -0.82 |
| P244^5.50^_Cα--N274^6.29^_Cα | -0.75 | C79^1.43^_Cα--I247^5.53^_Cα | -0.82 |
| I247^5.53^_Cα--K271^6.26^_Cα | -0.74 | G253^5.59^_Cα--V334^7.51^_Cα | -0.81 |
| I238^5.44^_Cα--K271^6.26^_Cα | -0.74 | I256^5.62^_Cα--V334^7.51^_Cα | -0.81 |
| N274^6.29^--L259^5.65^ | 0.74 | I256^5.62^_Cα--N328^7.45^_Cα | -0.80 |
| W226^5.31^_Cα--N274^6.29^_Cα | -0.74 | G85^1.49^_Cα--I247^5.53^_Cα | -0.80 |
| C235^5.41^_Cα--N274^6.29^_Cα | -0.74 | V250^5.56^_Cα--G325^7.42^_Cα | -0.80 |
| N274^6.29^_Cα--T307^ECL3^_Cα | -0.73 | A73^1.37^_Cα--P244^5.50^_Cα | -0.80 |
| G253^5.59^_Cα--N274^6.29^_Cα | -0.72 | G253^5.59^_Cα--V286^6.41^_Cα | -0.80 |
| E229^5.35^_Cα--N274^6.29^_Cα | -0.72 | A73^1.37^_Cα--I256^5.62^_Cα | -0.80 |
| F241^5.47^_Cα--K271^6.26^_Cα | -0.72 | G82^1.46^_Cα--P244^5.50^_Cα | -0.80 |
| K271^6.26^_Cα--V286^6.41^_Cα | -0.71 | T118^2.54^_Cα--G253^5.59^_Cα | -0.80 |
| L232^5.38^_Cα--N274^6.29^_Cα | -0.71 | I247^5.53^_Cα--N328^7.45^_Cα | -0.80 |
| W226^5.31^_Cα--K271^6.26^_Cα | -0.71 | L88^1.52^_Cα--G253^5.59^_Cα | -0.80 |
| T279^6.34^--V284^6.39^ | 0.70 | G82^1.46^_Cα--L259^5.65^_Cα | -0.80 |
| C235^5.41^_Cα--K271^6.26^_Cα | -0.69 | G85^1.49^_Cα--L259^5.65^_Cα | -0.80 |
| T279^6.34^--V282^6.37^ | -0.69 | S76^1.40^_Cα--P244^5.50^_Cα | -0.80 |
| N274^6.29^_Cα--L283^6.38^_Cα | -0.69 | T118^2.54^_Cα--V250^5.56^_Cα | -0.79 |
| P244^5.50^_Cα--K271^6.26^_Cα | -0.69 | V250^5.56^_Cα--V286^6.41^_Cα | -0.79 |
| R277^6.32^_Cα--F289^6.44^_Cα | -0.68 | I247^5.53^_Cα--G325^7.42^_Cα | -0.79 |
| L259^5.65^_Cα--S268^ICL3^_Cα | -0.68 | S76^1.40^_Cα--L259^5.65^_Cα | -0.79 |
| Y166^3.51^_Cα--N274^6.29^_Cα | -0.68 | G253^5.59^_Cα--G325^7.42^_Cα | -0.79 |
| N274^6.29^_Cα--F289^6.44^_Cα | -0.68 | C79^1.43^_Cα--P244^5.50^_Cα | -0.79 |
| E229^5.35^_Cα--K271^6.26^_Cα | -0.67 | L88^1.52^_Cα--V250^5.56^_Cα | -0.79 |
| R280^6.35^--L283^6.38^ | -0.67 | A73^1.37^_Cα--F241^5.47^_Cα | -0.79 |
| V262^ICL3^_Cα--R280^6.35^_Cα | 0.67 | C79^1.43^_Cα--L259^5.65^_Cα | -0.79 |
| I256^5.62^_Cα--N274^6.29^_Cα | -0.67 | L121^2.57^_Cα--G253^5.59^_Cα | -0.79 |
| K271^6.26^_Cα--L283^6.38^_Cα | -0.66 | A115^2.51^_Cα--G253^5.59^_Cα | -0.79 |
| H223^ECL2^_Cα--N274^6.29^_Cα | -0.66 | L121^2.57^_Cα--V250^5.56^_Cα | -0.78 |
| L232^5.38^_Cα--K271^6.26^_Cα | -0.66 | V250^5.56^_Cα--V334^7.51^_Cα | -0.78 |
| R277^6.32^--V282^6.37^ | 0.66 | V250^5.56^_Cα--F289^6.44^_Cα | -0.78 |
| V250^5.56^_Cα--R280^6.35^_Cα | 0.66 | L88^1.52^_Cα--I256^5.62^_Cα | -0.78 |
| I256^5.62^_Cα--S268^ICL3^_Cα | -0.66 | F241^5.47^_Cα--G325^7.42^_Cα | -0.78 |
| R277^6.32^_Cα--I298^6.53^_Cα | -0.65 | G253^5.59^_Cα--F343^H8^_Cα | -0.78 |
| R277^6.32^--R165^3.50^ | 0.65 | G253^5.59^_Cα--C346^H8^_Cα | -0.78 |
| L259^5.65^_Cα--N274^6.29^_Cα | -0.64 | T118^2.54^_Cα--I256^5.62^_Cα | -0.78 |
| G253^5.59^_Cα--S268^ICL3^_Cα | -0.64 | Y252^5.58^--Y336^7.53^ | 0.78 |
| I247^5.53^_Cα--R277^6.32^_Cα | -0.63 | I256^5.62^_Cα--F343^H8^_Cα | -0.78 |
| V163^3.48^_Cα--N274^6.29^_Cα | -0.63 | I256^5.62^_Cα--C346^H8^_Cα | -0.78 |
| K271^6.26^_Cα--C292^6.47^_Cα | -0.63 | A115^2.51^_Cα--V250^5.56^_Cα | -0.78 |
| R277^6.32^_Cα--A304^6.59^_Cα | -0.63 | L259^5.65^_Cα--L331^7.48^_Cα | -0.78 |
| I256^5.62^--L275^6.30^ | 0.62 | L121^2.57^_Cα--I247^5.53^_Cα | -0.78 |
| R277^6.32^_Cα--I301^6.56^_Cα | -0.62 | S76^1.40^_Cα--F241^5.47^_Cα | -0.78 |
| H223^ECL2^_Cα--K271^6.26^_Cα | -0.62 | I247^5.53^_Cα--L331^7.48^_Cα | -0.78 |
| I238^5.44^_Cα--R277^6.32^_Cα | -0.61 | L259^5.65^_Cα--C346^H8^_Cα | -0.78 |
| V250^5.56^_Cα--S268^ICL3^_Cα | -0.61 | L259^5.65^_Cα--F343^H8^_Cα | -0.78 |
| S261^5.67^--K271^6.26^ | 0.61 | T118^2.54^_Cα--I247^5.53^_Cα | -0.77 |
| F241^5.47^_Cα--R277^6.32^_Cα | -0.61 | L259^5.65^_Cα--E349^H8^_Cα | -0.77 |
| E229^5.35^_Cα--R277^6.32^_Cα | -0.61 | I256^5.62^_Cα--E349^H8^_Cα | -0.77 |
| K271^6.26^_Cα--F289^6.44^_Cα | -0.61 | L259^5.65^_Cα--V334^7.51^_Cα | -0.77 |
| W226^5.31^_Cα--R277^6.32^_Cα | -0.61 | A115^2.51^_Cα--I247^5.53^_Cα | -0.77 |
| N274^6.29^_Cα--C292^6.47^_Cα | -0.60 | G253^5.59^_Cα--E349^H8^_Cα | -0.77 |
| P244^5.50^_Cα--R277^6.32^_Cα | -0.60 | A115^2.51^_Cα--I256^5.62^_Cα | -0.77 |
| S261^5.67^--A264^ICL3^ | 0.60 | A73^1.37^_Cα--L259^5.65^_Cα | -0.77 |

**Table 2. Top 75 (~1%) structural descriptors of the slowest conformational degrees of freedom accessible to morphine-bound and TRV-130-bound MOR identified by tICA dimension 2. Structural descriptors involving correlated residue sidechains are highlighted in red. Commonalities, if any, between the morphine-bound MOR and TRV-130-bound MOR are highlighted in green.**

| **Morphine-bound MOR** | | **TRV-130-bound MOR** | |
| --- | --- | --- | --- |
| **Structural Descriptors** | **Correlation** | **Structural Descriptors** | **Correlation** |
| M281^6.36^--V286^6.41^ | -0.77 | T157^3.42^_Cα--L331^7.48^_Cα | 0.81 |
| V282^6.37^--A287^6.42^ | -0.75 | T157^3.42^_Cα--V334^7.51^_Cα | 0.80 |
| V282^6.37^--V285^6.40^ | 0.74 | L121^2.57^_Cα--C346^H8^_Cα | -0.78 |
| L283^6.38^--V286^6.41^ | 0.72 | A115^2.51^_Cα--C346^H8^_Cα | -0.78 |
| Y252^5.58^--M281^6.36^ | -0.71 | T118^2.54^_Cα--C346^H8^_Cα | -0.77 |
| I278^6.33^--L283^6.38^ | 0.64 | N127^2.63^_Cα--C346^H8^_Cα | -0.77 |
| V284^6.39^--A287^6.42^ | 0.64 | M130^2.66^_Cα--C346^H8^_Cα | -0.76 |
| I247^5.53^_Cα--L283^6.38^_Cα | -0.63 | Q124^2.60^_Cα--C346^H8^_Cα | -0.76 |
| R280^6.35^_Cα--V286^6.41^_Cα | 0.62 | A115^2.51^_Cα--E349^H8^_Cα | -0.76 |
| T279^6.34^--V282^6.37^ | -0.61 | G82^1.46^_Cα--C346^H8^_Cα | -0.75 |
| D340^H8^--R280^6.35^ | -0.58 | T67^1.31^_Cα--C346^H8^_Cα | -0.75 |
| L283^6.38^_Cα--V286^6.41^_Cα | -0.57 | S154^3.39^_Cα--L331^7.48^_Cα | 0.74 |
| R277^6.32^--V282^6.37^ | 0.57 | T70^1.34^_Cα--C346^H8^_Cα | -0.74 |
| R165^3.50^--V282^6.37^ | 0.57 | T118^2.54^_Cα--E349^H8^_Cα | -0.73 |
| Y166^3.51^_Cα--L283^6.38^_Cα | -0.56 | C79^1.43^_Cα--C346^H8^_Cα | -0.73 |
| V250^5.56^_Cα--L283^6.38^_Cα | -0.55 | T120^2.56^--N150^3.35^ | 0.73 |
| P244^5.50^_Cα--L283^6.38^_Cα | -0.55 | L121^2.57^_Cα--E349^H8^_Cα | -0.73 |
| R280^6.35^--L283^6.38^ | -0.54 | W133^ECL1^_Cα--C346^H8^_Cα | -0.73 |
| T279^6.34^--V284^6.39^ | 0.54 | A73^1.37^_Cα--C346^H8^_Cα | -0.72 |
| L283^6.38^_Cα--C292^6.47^_Cα | 0.53 | Q124^2.60^_Cα--E349^H8^_Cα | -0.72 |
| R280^6.35^_Cα--F289^6.44^_Cα | 0.52 | S64^Nterm^_Cα--C346^H8^_Cα | -0.72 |
| V163^3.48^_Cα--L283^6.38^_Cα | -0.52 | S214^ECL2^_Cα--C346^H8^_Cα | -0.72 |
| L283^6.38^--V288^6.43^ | -0.51 | S76^1.40^_Cα--C346^H8^_Cα | -0.72 |
| I278^6.33^--M281^6.36^ | -0.51 | N127^2.63^_Cα--E349^H8^_Cα | -0.71 |
| L283^6.38^_Cα--V316^7.33^_Cα | 0.50 | G85^1.49^_Cα--C346^H8^_Cα | -0.71 |
| V169^3.54^_Cα--L283^6.38^_Cα | -0.49 | F178^ICL2^_Cα--L331^7.48^_Cα | 0.70 |
| L283^6.38^_Cα--P295^6.50^_Cα | 0.48 | A111^2.47^--Y336^7.53^ | 0.70 |
| L283^6.38^_Cα--F313^7.30^_Cα | 0.48 | S214^ECL2^_Cα--E349^H8^_Cα | -0.70 |
| P172^ICL2^_Cα--L283^6.38^_Cα | -0.48 | T160^3.45^_Cα--V334^7.51^_Cα | 0.70 |
| R280^6.35^_Cα--L283^6.38^_Cα | 0.47 | L110^2.46^--N150^3.35^ | -0.70 |
| L283^6.38^_Cα--H319^7.36^_Cα | 0.47 | F178^ICL2^_Cα--A337^7.54^_Cα | 0.70 |
| Y252^5.58^--L283^6.38^ | 0.46 | N86^1.50^--Y336^7.53^ | 0.70 |
| N274^6.29^_Cα--L283^6.38^_Cα | -0.46 | F178^ICL2^_Cα--V334^7.51^_Cα | 0.70 |
| A175^ICL2^_Cα--L283^6.38^_Cα | -0.46 | L121^2.57^_Cα--F343^H8^_Cα | -0.70 |
| T160^3.45^_Cα--L283^6.38^_Cα | -0.43 | W133^ECL1^_Cα--E349^H8^_Cα | -0.70 |
| R280^6.35^--V285^6.40^ | -0.43 | T157^3.42^_Cα--N328^7.45^_Cα | 0.70 |
| F178^ICL2^_Cα--L283^6.38^_Cα | -0.43 | M130^2.66^_Cα--E349^H8^_Cα | -0.70 |
| Y128^2.64^--Y326^7.43^ | 0.42 | M130^2.66^_Cα--F343^H8^_Cα | -0.69 |
| I238^5.44^_Cα--L283^6.38^_Cα | -0.42 | T157^3.42^_Cα--A337^7.54^_Cα | 0.69 |
| L259^5.65^_Cα--L283^6.38^_Cα | -0.40 | S154^3.39^_Cα--V334^7.51^_Cα | 0.69 |
| V187^4.45^_Cα--L283^6.38^_Cα | -0.40 | T160^3.45^_Cα--L331^7.48^_Cα | 0.69 |
| F241^5.47^_Cα--L283^6.38^_Cα | -0.40 | N109^2.45^_Cα--S154^3.39^_Cα | -0.69 |
| A184^4.42^_Cα--L283^6.38^_Cα | -0.40 | G82^1.46^_Cα--E349^H8^_Cα | -0.69 |
| L121^2.57^--Y128^2.64^ | 0.39 | C217^ECL2^_Cα--E349^H8^_Cα | -0.68 |
| C190^4.48^_Cα--L283^6.38^_Cα | -0.39 | T103^2.39^_Cα--A337^7.54^_Cα | 0.68 |
| G253^5.59^_Cα--L283^6.38^_Cα | -0.39 | G82^1.46^_Cα--F343^H8^_Cα | -0.68 |
| I256^5.62^--L283^6.38^ | 0.39 | C79^1.43^_Cα--F343^H8^_Cα | -0.68 |
| C235^5.41^_Cα--L283^6.38^_Cα | -0.39 | K100^ICL1^_Cα--A337^7.54^_Cα | 0.68 |
| R280^6.35^_Cα--C292^6.47^_Cα | 0.39 | P181^4.39^_Cα--A337^7.54^_Cα | 0.68 |
| R277^6.32^_Cα--L283^6.38^_Cα | -0.39 | A184^4.42^_Cα--A337^7.54^_Cα | 0.68 |
| M161^3.46^--M281^6.36^ | -0.39 | T157^3.42^--Y336^7.53^ | -0.68 |
| P181^4.39^_Cα--L283^6.38^_Cα | -0.39 | T118^2.54^_Cα--F343^H8^_Cα | -0.68 |
| R280^6.35^--E341^H8^ | -0.38 | S76^1.40^_Cα--F343^H8^_Cα | -0.67 |
| E229^5.35^_Cα--R280^6.35^_Cα | 0.38 | N127^2.63^_Cα--F343^H8^_Cα | -0.67 |
| I256^5.62^--V282^6.37^ | -0.37 | A115^2.51^_Cα--F343^H8^_Cα | -0.67 |
| K271^6.26^_Cα--L283^6.38^_Cα | -0.37 | L88^1.52^--F350^H8^ | 0.67 |
| L232^5.38^_Cα--R280^6.35^_Cα | 0.37 | A73^1.37^_Cα--F343^H8^_Cα | -0.67 |
| I256^5.62^_Cα--L283^6.38^_Cα | -0.37 | V187^4.45^_Cα--A337^7.54^_Cα | 0.67 |
| R280^6.35^--R165^3.50^ | -0.37 | N328^7.45^_Cα--E349^H8^_Cα | -0.67 |
| I193^4.51^_Cα--L283^6.38^_Cα | -0.37 | T67^1.31^_Cα--E349^H8^_Cα | -0.67 |
| F241^5.47^_Cα--R280^6.35^_Cα | 0.37 | S64^Nterm^_Cα--E349^H8^_Cα | -0.67 |
| T157^3.42^_Cα--L283^6.38^_Cα | -0.37 | T97^ICL1^_Cα--A337^7.54^_Cα | 0.67 |
| Y128^2.64^--Morphine | 0.36 | L112^2.48^_Cα--C292^6.47^_Cα | -0.67 |
| C235^5.41^_Cα--R280^6.35^_Cα | 0.35 | V187^4.45^_Cα--V334^7.51^_Cα | 0.66 |
| W226^5.31^_Cα--R280^6.35^_Cα | 0.35 | C79^1.43^_Cα--E349^H8^_Cα | -0.66 |
| R280^6.35^_Cα--P295^6.50^_Cα | 0.35 | C190^4.48^_Cα--A337^7.54^_Cα | 0.66 |
| R280^6.35^_Cα--I298^6.53^_Cα | 0.35 | T67^1.31^_Cα--F343^H8^_Cα | -0.66 |
| R280^6.35^_Cα--A304^6.59^_Cα | 0.34 | Y96^ICL1^--F347^H8^ | -0.66 |
| H223^ECL2^_Cα--R280^6.35^_Cα | 0.34 | Q124^2.60^_Cα--F343^H8^_Cα | -0.66 |
| R280^6.35^_Cα--G325^7.42^_Cα | 0.34 | G85^1.49^_Cα--E349^H8^_Cα | -0.66 |
| Y128^2.64^--I322^7.39^ | 0.34 | T70^1.34^_Cα--F343^H8^_Cα | -0.65 |
| R280^6.35^_Cα--I301^6.56^_Cα | 0.34 | T103^2.39^_Cα--L331^7.48^_Cα | 0.65 |
| I238^5.44^_Cα--R280^6.35^_Cα | 0.34 | T70^1.34^_Cα--E349^H8^_Cα | -0.65 |
| L283^6.38^_Cα--E310^ECL3^_Cα | 0.34 | L112^2.48^_Cα--E349^H8^_Cα | -0.65 |
| S196^4.54^_Cα--L283^6.38^_Cα | -0.34 | A175^ICL2^_Cα--L331^7.48^_Cα | 0.65 |

**Table 3. Top 75 (~1%) structural descriptors of the slowest conformational degrees of freedom accessible to morphine-bound and TRV-130-bound MOR identified by tICA dimension 3. Structural descriptors involving correlated residue sidechains are highlighted in red. Commonalities, if any, between the morphine-bound MOR and TRV-130-bound MOR are highlighted in green.**

| **Morphine-bound MOR** | | **TRV-130-bound MOR** | |
| --- | --- | --- | --- |
| **Structural Descriptors** | **Correlation** | **Structural Descriptors** | **Correlation** |
| M99^ICL1^--I105^2.41^ | 0.74 | F241^5.47^_Cα--C292^6.47^_Cα | 0.72 |
| M99^ICL1^--F108^2.44^ | 0.72 | I238^5.44^_Cα--C292^6.47^_Cα | 0.70 |
| I93^1.57^--M99^ICL1^ | 0.72 | C235^5.41^_Cα--C292^6.47^_Cα | 0.70 |
| I256^5.62^_Cα--F343^H8^_Cα | 0.71 | F289^6.44^_Cα--P295^6.50^_Cα | -0.65 |
| L259^5.65^_Cα--F343^H8^_Cα | 0.71 | A240^5.46^--H297^6.52^ | -0.63 |
| K100^ICL1^_Cα--P295^6.50^_Cα | -0.71 | L232^5.38^_Cα--C292^6.47^_Cα | 0.62 |
| T103^2.39^--M99^ICL1^ | -0.71 | A184^4.42^_Cα--I298^6.53^_Cα | -0.60 |
| G253^5.59^_Cα--F343^H8^_Cα | 0.70 | V291^6.46^--T294^6.49^ | -0.60 |
| I256^5.62^_Cα--C346^H8^_Cα | 0.69 | L112^2.48^_Cα--I301^6.56^_Cα | -0.59 |
| T101^2.37^--N104^2.40^ | 0.69 | P244^5.50^_Cα--C292^6.47^_Cα | 0.59 |
| L259^5.65^_Cα--C346^H8^_Cα | 0.69 | V187^4.45^_Cα--I298^6.53^_Cα | -0.59 |
| L259^5.65^_Cα--E349^H8^_Cα | 0.68 | L112^2.48^_Cα--I298^6.53^_Cα | -0.59 |
| I256^5.62^_Cα--E349^H8^_Cα | 0.68 | Y166^3.51^_Cα--P295^6.50^_Cα | -0.59 |
| G253^5.59^_Cα--C346^H8^_Cα | 0.68 | V163^3.48^_Cα--P295^6.50^_Cα | -0.58 |
| K100^ICL1^_Cα--I298^6.53^_Cα | -0.68 | A184^4.42^_Cα--P295^6.50^_Cα | -0.58 |
| L259^5.65^_Cα--I352^H8^_Cα | 0.67 | P181^4.39^_Cα--I298^6.53^_Cα | -0.58 |
| G85^1.49^_Cα--I256^5.62^_Cα | 0.67 | V286^6.41^_Cα--P295^6.50^_Cα | -0.57 |
| V94^1.58^--M99^ICL1^ | 0.67 | V187^4.45^_Cα--P295^6.50^_Cα | -0.57 |
| G253^5.59^_Cα--E349^H8^_Cα | 0.66 | V169^3.54^_Cα--P295^6.50^_Cα | -0.57 |
| K100^ICL1^_Cα--Y106^2.42^_Cα | -0.66 | A184^4.42^_Cα--I301^6.56^_Cα | -0.57 |
| V250^5.56^_Cα--F343^H8^_Cα | 0.66 | P244^5.50^_Cα--V286^6.41^_Cα | 0.57 |
| I256^5.62^_Cα--I352^H8^_Cα | 0.66 | V187^4.45^_Cα--I301^6.56^_Cα | -0.56 |
| L88^1.52^_Cα--I256^5.62^_Cα | 0.66 | N109^2.45^_Cα--I298^6.53^_Cα | -0.56 |
| T103^2.39^_Cα--P295^6.50^_Cα | -0.66 | F241^5.47^--I290^6.45^ | -0.56 |
| G85^1.49^_Cα--L259^5.65^_Cα | 0.66 | P172^ICL2^_Cα--P295^6.50^_Cα | -0.56 |
| L88^1.52^_Cα--L259^5.65^_Cα | 0.65 | F289^6.44^--T294^6.49^ | 0.56 |
| V250^5.56^_Cα--C346^H8^_Cα | 0.65 | A115^2.51^_Cα--I298^6.53^_Cα | -0.55 |
| K100^ICL1^_Cα--I301^6.56^_Cα | -0.65 | A115^2.51^_Cα--I301^6.56^_Cα | -0.55 |
| Y91^1.55^_Cα--I256^5.62^_Cα | 0.65 | P181^4.39^_Cα--I301^6.56^_Cα | -0.55 |
| F178^ICL2^_Cα--S268^ICL3^_Cα | -0.65 | P181^4.39^_Cα--P295^6.50^_Cα | -0.55 |
| A175^ICL2^_Cα--S268^ICL3^_Cα | -0.64 | T160^3.45^_Cα--P295^6.50^_Cα | -0.55 |
| T160^3.45^_Cα--S268^ICL3^_Cα | -0.64 | N109^2.45^_Cα--I301^6.56^_Cα | -0.55 |
| G85^1.49^_Cα--G253^5.59^_Cα | 0.64 | A175^ICL2^_Cα--P295^6.50^_Cα | -0.55 |
| K98^ICL1^--K100^ICL1^ | 0.64 | T160^3.45^_Cα--I298^6.53^_Cα | -0.54 |
| P172^ICL2^_Cα--S268^ICL3^_Cα | -0.64 | F241^5.47^_Cα--V286^6.41^_Cα | 0.54 |
| T103^2.39^_Cα--I298^6.53^_Cα | -0.64 | A184^4.42^_Cα--P244^5.50^_Cα | -0.54 |
| T157^3.42^_Cα--S268^ICL3^_Cα | -0.64 | V163^3.48^_Cα--I298^6.53^_Cα | -0.54 |
| G253^5.59^_Cα--I352^H8^_Cα | 0.63 | C190^4.48^_Cα--I298^6.53^_Cα | -0.54 |
| L88^1.52^_Cα--G253^5.59^_Cα | 0.63 | M151^3.36^--F241^5.47^ | 0.53 |
| G82^1.46^_Cα--I256^5.62^_Cα | 0.63 | P181^4.39^_Cα--P244^5.50^_Cα | -0.53 |
| Y91^1.55^_Cα--L259^5.65^_Cα | 0.63 | Y166^3.51^_Cα--I298^6.53^_Cα | -0.53 |
| V250^5.56^_Cα--E349^H8^_Cα | 0.63 | I290^6.45^--P295^6.50^ | 0.53 |
| T103^2.39^_Cα--Y106^2.42^_Cα | -0.63 | C235^5.41^_Cα--V286^6.41^_Cα | 0.53 |
| V187^4.45^_Cα--S268^ICL3^_Cα | -0.63 | Y106^2.42^_Cα--I298^6.53^_Cα | -0.53 |
| V169^3.54^_Cα--S268^ICL3^_Cα | -0.63 | V94^1.58^_Cα--I301^6.56^_Cα | -0.53 |
| V163^3.48^_Cα--S268^ICL3^_Cα | -0.63 | I256^5.62^_Cα--P295^6.50^_Cα | -0.53 |
| A115^2.51^_Cα--I256^5.62^_Cα | 0.63 | Y91^1.55^_Cα--I301^6.56^_Cα | -0.52 |
| A184^4.42^_Cα--S268^ICL3^_Cα | -0.63 | L259^5.65^_Cα--P295^6.50^_Cα | -0.52 |
| C190^4.48^_Cα--S268^ICL3^_Cα | -0.63 | C190^4.48^_Cα--I301^6.56^_Cα | -0.52 |
| P181^4.39^_Cα--S268^ICL3^_Cα | -0.63 | V94^1.58^_Cα--I298^6.53^_Cα | -0.52 |
| I256^5.62^_Cα--D340^H8^_Cα | 0.62 | I238^5.44^_Cα--V286^6.41^_Cα | 0.52 |
| G82^1.46^_Cα--L259^5.65^_Cα | 0.62 | V169^3.54^_Cα--I298^6.53^_Cα | -0.52 |
| S154^3.39^_Cα--S268^ICL3^_Cα | -0.62 | V245^5.51^--I290^6.45^ | -0.52 |
| K100^ICL1^_Cα--F289^6.44^_Cα | -0.62 | L112^2.48^_Cα--A304^6.59^_Cα | -0.52 |
| L112^2.48^_Cα--I256^5.62^_Cα | 0.62 | Y91^1.55^_Cα--I298^6.53^_Cα | -0.52 |
| I193^4.51^_Cα--S268^ICL3^_Cα | -0.62 | L112^2.48^_Cα--P295^6.50^_Cα | -0.51 |
| T103^2.39^_Cα--I301^6.56^_Cα | -0.62 | A184^4.42^_Cα--F241^5.47^_Cα | -0.51 |
| I247^5.53^_Cα--F343^H8^_Cα | 0.62 | I256^5.62^_Cα--T307^ECL3^_Cα | -0.51 |
| Y91^1.55^_Cα--G253^5.59^_Cα | 0.61 | V187^4.45^_Cα--F241^5.47^_Cα | -0.51 |
| Y106^2.42^_Cα--I256^5.62^_Cα | 0.61 | I256^5.62^_Cα--I298^6.53^_Cα | -0.51 |
| N328^7.45^_Cα--D340^H8^_Cα | 0.61 | T97^ICL1^_Cα--I298^6.53^_Cα | -0.51 |
| A115^2.51^_Cα--L259^5.65^_Cα | 0.61 | A175^ICL2^_Cα--I298^6.53^_Cα | -0.51 |
| K100^ICL1^_Cα--F313^7.30^_Cα | -0.61 | N109^2.45^_Cα--P295^6.50^_Cα | -0.51 |
| K100^ICL1^_Cα--C292^6.47^_Cα | -0.61 | P172^ICL2^_Cα--I298^6.53^_Cα | -0.51 |
| Y106^2.42^_Cα--S268^ICL3^_Cα | -0.61 | Y106^2.42^_Cα--P295^6.50^_Cα | -0.50 |
| N109^2.45^_Cα--I256^5.62^_Cα | 0.61 | V187^4.45^_Cα--P244^5.50^_Cα | -0.50 |
| M99^ICL1^--C346^H8^ | -0.61 | L259^5.65^_Cα--T307^ECL3^_Cα | -0.50 |
| I247^5.53^_Cα--C346^H8^_Cα | 0.61 | I238^5.44^_Cα--L283^6.38^_Cα | 0.50 |
| S196^4.54^_Cα--S268^ICL3^_Cα | -0.61 | L259^5.65^_Cα--I298^6.53^_Cα | -0.50 |
| Y166^3.51^_Cα--S268^ICL3^_Cα | -0.61 | T157^3.42^_Cα--I298^6.53^_Cα | -0.50 |
| T103^2.39^_Cα--S154^3.39^_Cα | -0.61 | P181^4.39^_Cα--F241^5.47^_Cα | -0.50 |
| C79^1.43^_Cα--I256^5.62^_Cα | 0.61 | M151^3.36^_Cα--I301^6.56^_Cα | -0.50 |
| G253^5.59^_Cα--D340^H8^_Cα | 0.61 | C190^4.48^_Cα--P295^6.50^_Cα | -0.50 |
| I256^5.62^_Cα--A337^7.54^_Cα | 0.61 | V169^3.54^--L259^5.65^ | -0.49 |
| G82^1.46^_Cα--G253^5.59^_Cα | 0.61 | I193^4.51^_Cα--I301^6.56^_Cα | -0.49 |

**Table 4. Top 75 (~1%) structural descriptors of the slowest conformational degrees of freedom accessible to morphine-bound and TRV-130-bound MOR identified by tICA dimension 4. Structural descriptors involving correlated residue sidechains are highlighted in red. Commonalities, if any, between the morphine-bound MOR and TRV-130-bound MOR are highlighted in green.**

| **Morphine-bound MOR** | | **TRV-130-bound MOR** | |
| --- | --- | --- | --- |
| **Structural Descriptors** | **Correlation** | **Structural Descriptors** | **Correlation** |
| M99^ICL1^--I105^2.41^ | -0.55 | G136^ECL1^_Cα--R280^6.35^_Cα | -0.60 |
| I93^1.57^--M99^ICL1^ | -0.53 | L110^2.46^--V285^6.40^ | 0.60 |
| M99^ICL1^--F108^2.44^ | -0.52 | L139^3.24^_Cα--R280^6.35^_Cα | -0.59 |
| Y106^2.42^_Cα--V286^6.41^_Cα | -0.52 | Y148^3.33^_Cα--R280^6.35^_Cα | -0.58 |
| T103^2.39^--M99^ICL1^ | 0.52 | I142^3.27^_Cα--R280^6.35^_Cα | -0.58 |
| T101^2.37^--N104^2.40^ | -0.51 | G136^ECL1^_Cα--L283^6.38^_Cα | -0.58 |
| A175^ICL2^_Cα--V286^6.41^_Cα | -0.51 | L112^2.48^_Cα--R280^6.35^_Cα | -0.58 |
| N109^2.45^_Cα--V286^6.41^_Cα | -0.51 | S145^3.30^_Cα--R280^6.35^_Cα | -0.57 |
| V94^1.58^_Cα--V286^6.41^_Cα | -0.50 | G136^ECL1^_Cα--V286^6.41^_Cα | -0.57 |
| T157^3.42^_Cα--V286^6.41^_Cα | -0.50 | M151^3.36^_Cα--R280^6.35^_Cα | -0.57 |
| F178^ICL2^_Cα--V286^6.41^_Cα | -0.50 | Y148^3.33^_Cα--V286^6.41^_Cα | -0.57 |
| T97^ICL1^_Cα--V286^6.41^_Cα | -0.50 | A115^2.51^_Cα--L283^6.38^_Cα | -0.56 |
| T97^ICL1^_Cα--L283^6.38^_Cα | -0.49 | L139^3.24^_Cα--L283^6.38^_Cα | -0.56 |
| L112^2.48^_Cα--V286^6.41^_Cα | -0.49 | N109^2.45^_Cα--R280^6.35^_Cα | -0.56 |
| K100^ICL1^_Cα--L112^2.48^_Cα | 0.49 | L112^2.48^_Cα--L283^6.38^_Cα | -0.56 |
| K100^ICL1^_Cα--N109^2.45^_Cα | 0.49 | Y148^3.33^_Cα--L283^6.38^_Cα | -0.56 |
| Y106^2.42^_Cα--L283^6.38^_Cα | -0.49 | Y106^2.42^_Cα--R280^6.35^_Cα | -0.55 |
| Y91^1.55^_Cα--V286^6.41^_Cα | -0.48 | T208^ECL2^_Cα--R280^6.35^_Cα | -0.55 |
| L259^5.65^--L275^6.30^ | -0.48 | L139^3.24^_Cα--V286^6.41^_Cα | -0.55 |
| V94^1.58^_Cα--L283^6.38^_Cα | -0.48 | I142^3.27^_Cα--L283^6.38^_Cα | -0.55 |
| K100^ICL1^_Cα--A115^2.51^_Cα | 0.48 | T208^ECL2^_Cα--V286^6.41^_Cα | -0.55 |
| S154^3.39^_Cα--V286^6.41^_Cα | -0.48 | E229^5.35^_Cα--V286^6.41^_Cα | -0.55 |
| T160^3.45^_Cα--V286^6.41^_Cα | -0.48 | S154^3.39^_Cα--R280^6.35^_Cα | -0.55 |
| N109^2.45^_Cα--L283^6.38^_Cα | -0.48 | Y148^3.33^_Cα--R277^6.32^_Cα | -0.54 |
| V187^4.45^_Cα--V286^6.41^_Cα | -0.48 | T208^ECL2^_Cα--L283^6.38^_Cα | -0.54 |
| T157^3.42^_Cα--L283^6.38^_Cα | -0.47 | S145^3.30^_Cα--V286^6.41^_Cα | -0.54 |
| K98^ICL1^--K100^ICL1^ | -0.47 | V202^4.60^_Cα--R280^6.35^_Cα | -0.54 |
| C190^4.48^_Cα--V286^6.41^_Cα | -0.47 | I142^3.27^_Cα--V286^6.41^_Cα | -0.54 |
| A175^ICL2^_Cα--L283^6.38^_Cα | -0.46 | S145^3.30^_Cα--L283^6.38^_Cα | -0.54 |
| G85^1.49^_Cα--L259^5.65^_Cα | 0.46 | M151^3.36^_Cα--V286^6.41^_Cα | -0.54 |
| T160^3.45^_Cα--L283^6.38^_Cα | -0.46 | M151^3.36^_Cα--L283^6.38^_Cα | -0.54 |
| F178^ICL2^_Cα--L283^6.38^_Cα | -0.46 | S196^4.54^_Cα--R280^6.35^_Cα | -0.53 |
| G85^1.49^_Cα--I256^5.62^_Cα | 0.46 | A115^2.51^_Cα--R280^6.35^_Cα | -0.53 |
| K100^ICL1^_Cα--T118^2.54^_Cα | 0.46 | Y91^1.55^_Cα--R280^6.35^_Cα | -0.53 |
| V187^4.45^_Cα--L283^6.38^_Cα | -0.46 | V94^1.58^_Cα--R280^6.35^_Cα | -0.53 |
| S154^3.39^_Cα--L283^6.38^_Cα | -0.46 | N109^2.45^_Cα--L283^6.38^_Cα | -0.53 |
| M255^5.61^--I278^6.33^ | -0.46 | W133^ECL1^_Cα--V286^6.41^_Cα | -0.53 |
| Y252^5.58^--V285^6.40^ | -0.46 | T118^2.54^_Cα--L283^6.38^_Cα | -0.53 |
| C190^4.48^_Cα--L283^6.38^_Cα | -0.46 | M151^3.36^_Cα--R277^6.32^_Cα | -0.53 |
| V94^1.58^_Cα--D340^H8^_Cα | -0.46 | W226^5.31^_Cα--V286^6.41^_Cα | -0.53 |
| A115^2.51^_Cα--V286^6.41^_Cα | -0.46 | T97^ICL1^_Cα--R280^6.35^_Cα | -0.53 |
| A184^4.42^_Cα--V286^6.41^_Cα | -0.46 | V202^4.60^_Cα--V286^6.41^_Cα | -0.53 |
| G82^1.46^_Cα--I256^5.62^_Cα | 0.46 | G199^4.57^_Cα--R280^6.35^_Cα | -0.53 |
| G85^1.49^_Cα--G253^5.59^_Cα | 0.45 | M205^4.63^_Cα--R280^6.35^_Cα | -0.53 |
| S76^1.40^_Cα--K100^ICL1^_Cα | 0.45 | S196^4.54^_Cα--L283^6.38^_Cα | -0.52 |
| G82^1.46^_Cα--L259^5.65^_Cα | 0.45 | E229^5.35^_Cα--L283^6.38^_Cα | -0.52 |
| L112^2.48^_Cα--L283^6.38^_Cα | -0.45 | Y91^1.55^_Cα--L283^6.38^_Cα | -0.52 |
| G82^1.46^_Cα--G253^5.59^_Cα | 0.45 | C217^ECL2^_Cα--V286^6.41^_Cα | -0.52 |
| M151^3.36^_Cα--V286^6.41^_Cα | -0.45 | I142^3.27^_Cα--R277^6.32^_Cα | -0.52 |
| I193^4.51^_Cα--V286^6.41^_Cα | -0.45 | Y106^2.42^_Cα--L283^6.38^_Cα | -0.52 |
| C79^1.43^_Cα--K100^ICL1^_Cα | 0.45 | V202^4.60^_Cα--L283^6.38^_Cα | -0.52 |
| K100^ICL1^_Cα--H319^7.36^_Cα | 0.45 | G136^ECL1^_Cα--R277^6.32^_Cα | -0.52 |
| G82^1.46^_Cα--V250^5.56^_Cα | 0.45 | E229^5.35^_Cα--R280^6.35^_Cα | -0.52 |
| I193^4.51^_Cα--L283^6.38^_Cα | -0.45 | S145^3.30^_Cα--R277^6.32^_Cα | -0.52 |
| T103^2.39^_Cα--G253^5.59^_Cα | 0.45 | I298^6.53^_Cα--V334^7.51^_Cα | 0.52 |
| A184^4.42^_Cα--L283^6.38^_Cα | -0.45 | I298^6.53^_Cα--A337^7.54^_Cα | 0.52 |
| L88^1.52^_Cα--L259^5.65^_Cα | 0.45 | A184^4.42^_Cα--L283^6.38^_Cα | -0.52 |
| M151^3.36^_Cα--L283^6.38^_Cα | -0.45 | I193^4.51^_Cα--R280^6.35^_Cα | -0.52 |
| K100^ICL1^_Cα--L283^6.38^_Cα | -0.44 | F241^5.47^_Cα--I301^6.56^_Cα | 0.52 |
| C79^1.43^_Cα--L259^5.65^_Cα | 0.44 | I193^4.51^_Cα--L283^6.38^_Cα | -0.52 |
| G82^1.46^_Cα--I247^5.53^_Cα | 0.44 | C217^ECL2^_Cα--L283^6.38^_Cα | -0.52 |
| K100^ICL1^_Cα--G136^ECL1^_Cα | 0.44 | Y148^3.33^_Cα--F289^6.44^_Cα | -0.52 |
| G85^1.49^_Cα--V250^5.56^_Cα | 0.44 | T103^2.39^_Cα--R280^6.35^_Cα | -0.51 |
| I142^3.27^_Cα--V286^6.41^_Cα | -0.44 | A184^4.42^_Cα--R280^6.35^_Cα | -0.51 |
| C79^1.43^_Cα--I256^5.62^_Cα | 0.44 | W226^5.31^_Cα--C292^6.47^_Cα | -0.51 |
| K100^ICL1^_Cα--M151^3.36^_Cα | 0.44 | G199^4.57^_Cα--L283^6.38^_Cα | -0.51 |
| L88^1.52^_Cα--I256^5.62^_Cα | 0.44 | P244^5.50^_Cα--I301^6.56^_Cα | 0.51 |
| G82^1.46^_Cα--P244^5.50^_Cα | 0.44 | S154^3.39^_Cα--L283^6.38^_Cα | -0.51 |
| V163^3.48^_Cα--L283^6.38^_Cα | -0.44 | W133^ECL1^_Cα--L283^6.38^_Cα | -0.51 |
| V94^1.58^--M99^ICL1^ | -0.44 | L139^3.24^_Cα--R277^6.32^_Cα | -0.51 |
| S196^4.54^_Cα--L283^6.38^_Cα | -0.44 | M205^4.63^_Cα--L283^6.38^_Cα | -0.51 |
| S145^3.30^_Cα--V286^6.41^_Cα | -0.44 | V187^4.45^_Cα--L283^6.38^_Cα | -0.51 |
| K100^ICL1^_Cα--M130^2.66^_Cα | 0.44 | S154^3.39^_Cα--R277^6.32^_Cα | -0.51 |
| M99^ICL1^--C346^H8^ | 0.44 | C190^4.48^_Cα--L283^6.38^_Cα | -0.51 |
| S196^4.54^_Cα--V286^6.41^_Cα | -0.44 | L121^2.57^_Cα--L283^6.38^_Cα | -0.51 |

**Table 5. Top 75 (~1%) structural descriptors of the slowest conformational degrees of freedom accessible to morphine-bound and TRV-130-bound MOR identified by tICA dimension 5. Structural descriptors involving correlated residue sidechains are highlighted in red. Commonalities, if any, between the morphine-bound MOR and TRV-130-bound MOR are highlighted in green.**

| **Morphine-bound MOR** | | **TRV-130-bound MOR** | |
| --- | --- | --- | --- |
| **Structural Descriptors** | **Correlation** | **Structural Descriptors** | **Correlation** |
| V288^6.43^--L335^7.52^ | -0.91 | Y299^6.54^--C321^7.38^ | -0.63 |
| V286^6.41^--L335^7.52^ | 0.87 | L283^6.38^_Cα--H319^7.36^_Cα | -0.63 |
| T118^2.54^--C330^7.47^ | -0.82 | L283^6.38^_Cα--I322^7.39^_Cα | -0.61 |
| G82^1.46^_Cα--L331^7.48^_Cα | 0.81 | V286^6.41^_Cα--H319^7.36^_Cα | -0.60 |
| V78^1.42^--T327^7.44^ | -0.81 | C292^6.47^--C321^7.38^ | 0.59 |
| G82^1.46^_Cα--N328^7.45^_Cα | 0.79 | C292^6.47^_Cα--H319^7.36^_Cα | -0.59 |
| G85^1.49^_Cα--L331^7.48^_Cα | 0.75 | E229^5.35^_Cα--N328^7.45^_Cα | 0.58 |
| C79^1.43^_Cα--L331^7.48^_Cα | 0.74 | V286^6.41^_Cα--I322^7.39^_Cα | -0.58 |
| S76^1.40^_Cα--L331^7.48^_Cα | 0.73 | E229^5.35^_Cα--G325^7.42^_Cα | 0.57 |
| A115^2.51^_Cα--C292^6.47^_Cα | 0.73 | F289^6.44^_Cα--H319^7.36^_Cα | -0.57 |
| G85^1.49^_Cα--V334^7.51^_Cα | 0.72 | L283^6.38^_Cα--V316^7.33^_Cα | -0.56 |
| C79^1.43^_Cα--N328^7.45^_Cα | 0.72 | V169^3.54^_Cα--I322^7.39^_Cα | -0.56 |
| Y91^1.55^_Cα--C292^6.47^_Cα | 0.72 | L283^6.38^_Cα--E310^ECL3^_Cα | -0.56 |
| T118^2.54^_Cα--L331^7.48^_Cα | 0.72 | F289^6.44^_Cα--I322^7.39^_Cα | -0.55 |
| S76^1.40^_Cα--N328^7.45^_Cα | 0.72 | W226^5.31^_Cα--N328^7.45^_Cα | 0.55 |
| T118^2.54^_Cα--N328^7.45^_Cα | 0.72 | A117^2.53^--S329^7.46^ | -0.54 |
| A115^2.51^_Cα--L331^7.48^_Cα | 0.71 | W226^5.31^_Cα--G325^7.42^_Cα | 0.54 |
| L88^1.52^_Cα--V334^7.51^_Cα | 0.71 | A175^ICL2^_Cα--I322^7.39^_Cα | -0.54 |
| L112^2.48^_Cα--C292^6.47^_Cα | 0.70 | R280^6.35^_Cα--I322^7.39^_Cα | -0.54 |
| L88^1.52^_Cα--C292^6.47^_Cα | 0.70 | E229^5.35^_Cα--L331^7.48^_Cα | 0.54 |
| V288^6.43^--N332^7.49^ | -0.70 | V286^6.41^_Cα--V316^7.33^_Cα | -0.54 |
| L121^2.57^_Cα--L331^7.48^_Cα | 0.70 | C292^6.47^_Cα--I322^7.39^_Cα | -0.54 |
| L88^1.52^_Cα--L331^7.48^_Cα | 0.70 | A304^6.59^_Cα--N328^7.45^_Cα | 0.53 |
| A115^2.51^_Cα--N328^7.45^_Cα | 0.69 | C292^6.47^_Cα--V316^7.33^_Cα | -0.53 |
| L121^2.57^_Cα--N328^7.45^_Cα | 0.69 | L283^6.38^_Cα--G325^7.42^_Cα | -0.53 |
| G85^1.49^_Cα--N328^7.45^_Cα | 0.68 | R280^6.35^_Cα--E310^ECL3^_Cα | -0.53 |
| V81^1.45^--C330^7.47^ | -0.68 | A304^6.59^_Cα--L331^7.48^_Cα | 0.52 |
| V286^6.41^_Cα--L331^7.48^_Cα | -0.68 | Y166^3.51^_Cα--I322^7.39^_Cα | -0.52 |
| G82^1.46^_Cα--V334^7.51^_Cα | 0.68 | W226^5.31^_Cα--L331^7.48^_Cα | 0.52 |
| A73^1.37^_Cα--L331^7.48^_Cα | 0.67 | T307^ECL3^_Cα--F313^7.30^_Cα | 0.52 |
| L88^1.52^_Cα--N328^7.45^_Cα | 0.67 | P295^6.50^--L324^7.41^ | -0.52 |
| W293^6.48^--S329^7.46^ | -0.67 | T103^2.39^_Cα--H319^7.36^_Cα | -0.52 |
| S145^3.30^_Cα--F289^6.44^_Cα | 0.66 | K271^6.26^_Cα--E310^ECL3^_Cα | -0.52 |
| I142^3.27^_Cα--F289^6.44^_Cα | 0.66 | F289^6.44^_Cα--V316^7.33^_Cα | -0.52 |
| L139^3.24^_Cα--F289^6.44^_Cα | 0.66 | T103^2.39^_Cα--I322^7.39^_Cα | -0.52 |
| I142^3.27^_Cα--C292^6.47^_Cα | 0.65 | I301^6.56^_Cα--N328^7.45^_Cα | 0.52 |
| L139^3.24^_Cα--C292^6.47^_Cα | 0.65 | Y299^6.54^--S317^7.34^ | -0.52 |
| Q124^2.60^_Cα--L331^7.48^_Cα | 0.65 | T160^3.45^_Cα--I322^7.39^_Cα | -0.52 |
| Y148^3.33^_Cα--F289^6.44^_Cα | 0.65 | P172^ICL2^_Cα--I322^7.39^_Cα | -0.52 |
| G136^ECL1^_Cα--L331^7.48^_Cα | 0.64 | V163^3.48^_Cα--I322^7.39^_Cα | -0.52 |
| N127^2.63^_Cα--L331^7.48^_Cα | 0.64 | L283^6.38^_Cα--F313^7.30^_Cα | -0.51 |
| G136^ECL1^_Cα--F289^6.44^_Cα | 0.64 | R280^6.35^_Cα--H319^7.36^_Cα | -0.51 |
| W133^ECL1^_Cα--L331^7.48^_Cα | 0.64 | I301^6.56^_Cα--L331^7.48^_Cα | 0.51 |
| G82^1.46^_Cα--C292^6.47^_Cα | 0.63 | V169^3.54^_Cα--H319^7.36^_Cα | -0.51 |
| G85^1.49^_Cα--C292^6.47^_Cα | 0.63 | A175^ICL2^_Cα--H319^7.36^_Cα | -0.51 |
| Y91^1.55^_Cα--V334^7.51^_Cα | 0.63 | F178^ICL2^_Cα--I322^7.39^_Cα | -0.51 |
| D114^2.50^--W293^6.48^ | -0.63 | H223^ECL2^_Cα--G325^7.42^_Cα | 0.51 |
| Q124^2.60^_Cα--N328^7.45^_Cα | 0.63 | C292^6.47^_Cα--A337^7.54^_Cα | 0.51 |
| L139^3.24^_Cα--L331^7.48^_Cα | 0.62 | A304^6.59^_Cα--G325^7.42^_Cα | 0.51 |
| V202^4.60^_Cα--F289^6.44^_Cα | 0.62 | C292^6.47^_Cα--D340^H8^_Cα | 0.51 |
| N109^2.45^_Cα--C292^6.47^_Cα | 0.62 | V286^6.41^_Cα--E310^ECL3^_Cα | -0.51 |
| C79^1.43^_Cα--V334^7.51^_Cα | 0.62 | V163^3.48^_Cα--H319^7.36^_Cα | -0.50 |
| S76^1.40^_Cα--V334^7.51^_Cα | 0.62 | H223^ECL2^_Cα--N328^7.45^_Cα | 0.50 |
| A115^2.51^_Cα--V334^7.51^_Cα | 0.62 | T157^3.42^_Cα--I322^7.39^_Cα | -0.50 |
| T118^2.54^_Cα--C292^6.47^_Cα | 0.62 | T103^2.39^_Cα--V334^7.51^_Cα | -0.50 |
| T118^2.54^_Cα--V334^7.51^_Cα | 0.62 | T160^3.45^_Cα--H319^7.36^_Cα | -0.50 |
| Y91^1.55^_Cα--N328^7.45^_Cα | 0.62 | F178^ICL2^_Cα--H319^7.36^_Cα | -0.50 |
| Y91^1.55^_Cα--L331^7.48^_Cα | 0.62 | Y166^3.51^_Cα--H319^7.36^_Cα | -0.50 |
| G136^ECL1^_Cα--N328^7.45^_Cα | 0.62 | N274^6.29^_Cα--E310^ECL3^_Cα | -0.49 |
| M130^2.66^_Cα--L331^7.48^_Cα | 0.62 | F289^6.44^_Cα--D340^H8^_Cα | 0.49 |
| A73^1.37^_Cα--N328^7.45^_Cα | 0.61 | W226^5.31^_Cα--V334^7.51^_Cα | 0.49 |
| S145^3.30^_Cα--C292^6.47^_Cα | 0.61 | A115^2.51^_Cα--N328^7.45^_Cα | 0.49 |
| A175^ICL2^_Cα--L331^7.48^_Cα | -0.61 | N86^1.50^--N332^7.49^ | -0.49 |
| L139^3.24^_Cα--N328^7.45^_Cα | 0.61 | C292^6.47^_Cα--F313^7.30^_Cα | -0.49 |
| V163^3.48^_Cα--L331^7.48^_Cα | -0.61 | C292^6.47^_Cα--V334^7.51^_Cα | 0.49 |
| M151^3.36^_Cα--F289^6.44^_Cα | 0.61 | K271^6.26^_Cα--I322^7.39^_Cα | -0.49 |
| V284^6.39^--L335^7.52^ | -0.61 | F289^6.44^_Cα--F343^H8^_Cα | 0.49 |
| V169^3.54^_Cα--L331^7.48^_Cα | -0.60 | T157^3.42^_Cα--H319^7.36^_Cα | -0.49 |
| V288^6.43^--N328^7.45^ | -0.60 | R280^6.35^_Cα--G325^7.42^_Cα | -0.48 |
| L121^2.57^_Cα--V334^7.51^_Cα | 0.60 | Y106^2.42^_Cα--H319^7.36^_Cα | -0.48 |
| L335^7.52^--R165^3.50^ | 0.60 | C292^6.47^_Cα--E310^ECL3^_Cα | -0.48 |
| Y166^3.51^_Cα--L331^7.48^_Cα | -0.60 | V286^6.41^_Cα--F313^7.30^_Cα | -0.48 |
| T208^ECL2^_Cα--F289^6.44^_Cα | 0.60 | E229^5.35^_Cα--V334^7.51^_Cα | 0.48 |
| N127^2.63^_Cα--N328^7.45^_Cα | 0.60 | F289^6.44^_Cα--E310^ECL3^_Cα | -0.48 |
| A73^1.37^_Cα--V334^7.51^_Cα | 0.59 | R280^6.35^_Cα--V316^7.33^_Cα | -0.48 |

**Table 6. Top 75 (~1%) structural descriptors of the slowest conformational degrees of freedom accessible to morphine-bound and TRV-130-bound MOR identified by tICA dimension 6. Structural descriptors involving correlated residue sidechains are highlighted in red. Commonalities, if any, between the morphine-bound MOR and TRV-130-bound MOR are highlighted in green.**

| **Morphine-bound MOR** | | **TRV-130-bound MOR** | |
| --- | --- | --- | --- |
| **Structural Descriptors** | **Correlation** | **Structural Descriptors** | **Correlation** |
| R165^3.50^--Y252^5.58^ | 0.56 | V286^6.41^--V291^6.46^ | -0.50 |
| M265^ICL3^_Cα--V334^7.51^_Cα | -0.52 | V285^6.40^--I290^6.45^ | -0.49 |
| M265^ICL3^_Cα--L331^7.48^_Cα | -0.51 | M151^3.36^--W293^6.48^ | 0.47 |
| M265^ICL3^_Cα--D340^H8^_Cα | -0.50 | N328^7.45^--TRV-130 | 0.44 |
| A73^1.37^_Cα--M265^ICL3^_Cα | -0.50 | V288^6.43^--V291^6.46^ | 0.44 |
| M265^ICL3^_Cα--N328^7.45^_Cα | -0.50 | W293^6.48^--S329^7.46^ | -0.43 |
| S76^1.40^_Cα--M265^ICL3^_Cα | -0.49 | P244^5.50^_Cα--V262^ICL3^_Cα | 0.41 |
| T70^1.34^_Cα--M265^ICL3^_Cα | -0.49 | F241^5.47^_Cα--V262^ICL3^_Cα | 0.41 |
| M265^ICL3^_Cα--V286^6.41^_Cα | -0.49 | I256^5.62^_Cα--V262^ICL3^_Cα | 0.41 |
| M265^ICL3^_Cα--A337^7.54^_Cα | -0.48 | S196^4.54^_Cα--C292^6.47^_Cα | -0.41 |
| M265^ICL3^_Cα--H319^7.36^_Cα | -0.48 | W293^6.48^--Y326^7.43^ | -0.41 |
| C79^1.43^_Cα--M265^ICL3^_Cα | -0.48 | L259^5.65^_Cα--V262^ICL3^_Cα | 0.41 |
| G82^1.46^_Cα--M265^ICL3^_Cα | -0.48 | V169^3.54^_Cα--V187^4.45^_Cα | -0.40 |
| R263^ICL3^--D272^6.27^ | -0.48 | F152^3.37^--A240^5.46^ | 0.40 |
| T67^1.31^_Cα--M265^ICL3^_Cα | -0.48 | I193^4.51^_Cα--C292^6.47^_Cα | -0.40 |
| M265^ICL3^_Cα--L283^6.38^_Cα | -0.48 | V169^3.54^_Cα--S196^4.54^_Cα | -0.40 |
| M265^ICL3^_Cα--I322^7.39^_Cα | -0.48 | S329^7.46^--TRV-130 | 0.40 |
| M265^ICL3^_Cα--G325^7.42^_Cα | -0.48 | V169^3.54^_Cα--I193^4.51^_Cα | -0.40 |
| G85^1.49^_Cα--M265^ICL3^_Cα | -0.48 | E229^5.35^_Cα--V262^ICL3^_Cα | 0.39 |
| M265^ICL3^_Cα--C292^6.47^_Cα | -0.47 | V163^3.48^_Cα--S196^4.54^_Cα | -0.39 |
| M265^ICL3^_Cα--V316^7.33^_Cα | -0.47 | M265^ICL3^_Cα--I301^6.56^_Cα | 0.39 |
| M265^ICL3^_Cα--F343^H8^_Cα | -0.47 | V262^ICL3^_Cα--I301^6.56^_Cα | 0.39 |
| M265^ICL3^_Cα--F289^6.44^_Cα | -0.47 | W226^5.31^_Cα--V262^ICL3^_Cα | 0.39 |
| M161^3.46^--Y252^5.58^ | 0.47 | M265^ICL3^_Cα--I298^6.53^_Cα | 0.39 |
| M130^2.66^_Cα--M265^ICL3^_Cα | -0.47 | V169^3.54^_Cα--A184^4.42^_Cα | -0.39 |
| L259^5.65^_Cα--V262^ICL3^_Cα | -0.46 | I247^5.53^_Cα--V262^ICL3^_Cα | 0.39 |
| I256^5.62^_Cα--V262^ICL3^_Cα | -0.46 | Y166^3.51^_Cα--S196^4.54^_Cα | -0.39 |
| M265^ICL3^_Cα--R277^6.32^_Cα | -0.46 | T160^3.45^_Cα--S196^4.54^_Cα | -0.39 |
| V285^6.40^--I290^6.45^ | -0.46 | G253^5.59^_Cα--V262^ICL3^_Cα | 0.38 |
| T118^2.54^_Cα--M265^ICL3^_Cα | -0.46 | M265^ICL3^_Cα--T307^ECL3^_Cα | 0.38 |
| L121^2.57^_Cα--M265^ICL3^_Cα | -0.46 | M265^ICL3^_Cα--A304^6.59^_Cα | 0.38 |
| V286^6.41^_Cα--P295^6.50^_Cα | 0.45 | L232^5.38^_Cα--V262^ICL3^_Cα | 0.38 |
| N127^2.63^_Cα--M265^ICL3^_Cα | -0.45 | S196^4.54^_Cα--G253^5.59^_Cα | -0.38 |
| L88^1.52^_Cα--M265^ICL3^_Cα | -0.45 | I238^5.44^_Cα--V262^ICL3^_Cα | 0.38 |
| M265^ICL3^_Cα--R280^6.35^_Cα | -0.45 | T160^3.45^_Cα--I193^4.51^_Cα | -0.38 |
| Q124^2.60^_Cα--M265^ICL3^_Cα | -0.45 | V262^ICL3^_Cα--A304^6.59^_Cα | 0.38 |
| V286^6.41^--V291^6.46^ | -0.45 | S196^4.54^_Cα--I256^5.62^_Cα | -0.38 |
| A287^6.42^--I290^6.45^ | 0.45 | F152^3.37^--V236^5.42^ | 0.38 |
| V286^6.41^--F289^6.44^ | 0.45 | V163^3.48^_Cα--I193^4.51^_Cα | -0.37 |
| V163^3.48^_Cα--V262^ICL3^_Cα | -0.45 | G253^5.59^_Cα--M265^ICL3^_Cα | 0.37 |
| M265^ICL3^_Cα--C346^H8^_Cα | -0.45 | M265^ICL3^_Cα--P295^6.50^_Cα | 0.37 |
| T157^3.42^_Cα--V262^ICL3^_Cα | -0.44 | M151^3.36^_Cα--C292^6.47^_Cα | -0.37 |
| T160^3.45^_Cα--V262^ICL3^_Cα | -0.44 | V163^3.48^_Cα--V262^ICL3^_Cα | 0.37 |
| Y166^3.51^_Cα--V262^ICL3^_Cα | -0.44 | C235^5.41^_Cα--V262^ICL3^_Cα | 0.37 |
| V286^6.41^_Cα--I298^6.53^_Cα | 0.44 | Y166^3.51^_Cα--I193^4.51^_Cα | -0.37 |
| V285^6.40^--L335^7.52^ | 0.44 | I256^5.62^_Cα--M265^ICL3^_Cα | 0.37 |
| P244^5.50^_Cα--V262^ICL3^_Cα | -0.44 | A287^6.42^--C292^6.47^ | -0.37 |
| M151^3.36^_Cα--V262^ICL3^_Cα | -0.44 | V250^5.56^_Cα--V262^ICL3^_Cα | 0.37 |
| S214^ECL2^_Cα--M265^ICL3^_Cα | -0.44 | G199^4.57^_Cα--C292^6.47^_Cα | -0.37 |
| M265^ICL3^_Cα--F313^7.30^_Cα | -0.43 | D114^2.50^--TRV-130 | 0.37 |
| M265^ICL3^_Cα--E349^H8^_Cα | -0.43 | Y166^3.51^_Cα--V187^4.45^_Cα | -0.37 |
| M265^ICL3^_Cα--P295^6.50^_Cα | -0.43 | Y166^3.51^_Cα--V262^ICL3^_Cα | 0.37 |
| W133^ECL1^_Cα--M265^ICL3^_Cα | -0.43 | V262^ICL3^_Cα--I298^6.53^_Cα | 0.37 |
| Y148^3.33^_Cα--V262^ICL3^_Cα | -0.43 | I193^4.51^_Cα--I256^5.62^_Cα | -0.36 |
| A115^2.51^_Cα--M265^ICL3^_Cα | -0.43 | G199^4.57^_Cα--P295^6.50^_Cα | -0.36 |
| M265^ICL3^_Cα--I352^H8^_Cα | -0.43 | V169^3.54^_Cα--C190^4.48^_Cα | -0.36 |
| V262^ICL3^_Cα--G325^7.42^_Cα | -0.43 | Y148^3.33^_Cα--V262^ICL3^_Cα | 0.36 |
| S154^3.39^_Cα--V262^ICL3^_Cα | -0.43 | F156^3.41^--I198^4.56^ | 0.36 |
| S64^Nterm^_Cα--M265^ICL3^_Cα | -0.43 | T160^3.45^_Cα--V262^ICL3^_Cα | 0.36 |
| C190^4.48^--F156^3.41^ | 0.43 | M265^ICL3^_Cα--E310^ECL3^_Cα | 0.36 |
| G136^ECL1^_Cα--V262^ICL3^_Cα | -0.43 | P172^ICL2^_Cα--V187^4.45^_Cα | -0.36 |
| V262^ICL3^_Cα--I322^7.39^_Cα | -0.43 | V187^4.45^_Cα--I256^5.62^_Cα | -0.35 |
| L139^3.24^_Cα--V262^ICL3^_Cα | -0.43 | S196^4.54^_Cα--P295^6.50^_Cα | -0.35 |
| I247^5.53^_Cα--V262^ICL3^_Cα | -0.43 | P172^ICL2^_Cα--S196^4.54^_Cα | -0.35 |
| S145^3.30^_Cα--V262^ICL3^_Cα | -0.43 | P172^ICL2^_Cα--I193^4.51^_Cα | -0.35 |
| I142^3.27^_Cα--V262^ICL3^_Cα | -0.42 | Y91^1.55^_Cα--K100^ICL1^_Cα | -0.35 |
| V262^ICL3^_Cα--N328^7.45^_Cα | -0.42 | A175^ICL2^_Cα--A184^4.42^_Cα | -0.35 |
| M265^ICL3^_Cα--N274^6.29^_Cα | -0.42 | M151^3.36^_Cα--V262^ICL3^_Cα | 0.35 |
| V262^ICL3^_Cα--F289^6.44^_Cα | -0.42 | N332^7.49^--TRV-130 | 0.35 |
| V262^ICL3^_Cα--V286^6.41^_Cα | -0.42 | A287^6.42^--I290^6.45^ | 0.35 |
| W133^ECL1^_Cα--V262^ICL3^_Cα | -0.42 | F241^5.47^_Cα--M265^ICL3^_Cα | 0.35 |
| F241^5.47^_Cα--V262^ICL3^_Cα | -0.42 | W133^ECL1^_Cα--G136^ECL1^_Cα | -0.35 |
| V262^ICL3^_Cα--D340^H8^_Cα | -0.42 | L88^1.52^_Cα--K100^ICL1^_Cα | -0.35 |
| V286^6.41^_Cα--V334^7.51^_Cα | -0.42 | V262^ICL3^_Cα--P295^6.50^_Cα | 0.35 |
| Y91^1.55^_Cα--M265^ICL3^_Cα | -0.42 | Y148^3.33^_Cα--A184^4.42^_Cα | 0.35 |

**Table 7. Top 75 (~1%) structural descriptors of the slowest conformational degrees of freedom accessible to morphine-bound and TRV-130-bound MOR identified by tICA dimension 7. Structural descriptors involving correlated residue sidechains are highlighted in red. Commonalities, if any, between the morphine-bound MOR and TRV-130-bound MOR are highlighted in green.**

| **Morphine-bound MOR** | | **TRV-130-bound MOR** | |
| --- | --- | --- | --- |
| **Structural Descriptors** | **Correlation** | **Structural Descriptors** | **Correlation** |
| V285^6.40^--I290^6.45^ | -0.73 | V286^6.41^--V291^6.46^ | -0.74 |
| V286^6.41^--V291^6.46^ | -0.72 | V285^6.40^--I290^6.45^ | -0.73 |
| A287^6.42^--I290^6.45^ | 0.71 | V288^6.43^--V291^6.46^ | 0.64 |
| V286^6.41^--F289^6.44^ | 0.70 | A287^6.42^--C292^6.47^ | -0.55 |
| V285^6.40^--L335^7.52^ | 0.58 | A287^6.42^--I290^6.45^ | 0.53 |
| V284^6.39^--F289^6.44^ | -0.51 | V286^6.41^--F289^6.44^ | 0.47 |
| M265^ICL3^_Cα--P295^6.50^_Cα | 0.46 | Y252^5.58^--V286^6.41^ | 0.41 |
| I248^5.54^--V286^6.41^ | 0.46 | V262^ICL3^_Cα--I301^6.56^_Cα | -0.34 |
| M265^ICL3^_Cα--I298^6.53^_Cα | 0.46 | V286^6.41^_Cα--C292^6.47^_Cα | 0.34 |
| M265^ICL3^_Cα--H319^7.36^_Cα | 0.45 | V262^ICL3^_Cα--I298^6.53^_Cα | -0.33 |
| M265^ICL3^_Cα--I322^7.39^_Cα | 0.45 | K260^5.66^--L275^6.30^ | -0.33 |
| M265^ICL3^_Cα--V316^7.33^_Cα | 0.45 | V262^ICL3^_Cα--A304^6.59^_Cα | -0.32 |
| M265^ICL3^_Cα--C292^6.47^_Cα | 0.45 | T208^ECL2^_Cα--V262^ICL3^_Cα | -0.32 |
| M265^ICL3^_Cα--F289^6.44^_Cα | 0.45 | F241^5.47^_Cα--V262^ICL3^_Cα | -0.32 |
| M265^ICL3^_Cα--G325^7.42^_Cα | 0.45 | C217^ECL2^_Cα--V262^ICL3^_Cα | -0.32 |
| M265^ICL3^_Cα--T307^ECL3^_Cα | 0.45 | S145^3.30^_Cα--V262^ICL3^_Cα | -0.31 |
| M265^ICL3^_Cα--E310^ECL3^_Cα | 0.45 | I256^5.62^_Cα--V262^ICL3^_Cα | -0.31 |
| M265^ICL3^_Cα--F313^7.30^_Cα | 0.45 | V262^ICL3^_Cα--L283^6.38^_Cα | -0.31 |
| G253^5.59^_Cα--M265^ICL3^_Cα | 0.45 | M151^3.36^_Cα--V262^ICL3^_Cα | -0.31 |
| I256^5.62^_Cα--M265^ICL3^_Cα | 0.45 | S154^3.39^_Cα--V262^ICL3^_Cα | -0.31 |
| L259^5.65^_Cα--M265^ICL3^_Cα | 0.44 | Y148^3.33^_Cα--V262^ICL3^_Cα | -0.31 |
| V286^6.41^_Cα--P295^6.50^_Cα | 0.44 | V262^ICL3^_Cα--F313^7.30^_Cα | -0.31 |
| M265^ICL3^_Cα--N328^7.45^_Cα | 0.44 | V262^ICL3^_Cα--P295^6.50^_Cα | -0.31 |
| M265^ICL3^_Cα--I301^6.56^_Cα | 0.43 | Y166^3.51^_Cα--V262^ICL3^_Cα | -0.31 |
| F241^5.47^_Cα--M265^ICL3^_Cα | 0.43 | P244^5.50^_Cα--V262^ICL3^_Cα | -0.31 |
| V250^5.56^_Cα--M265^ICL3^_Cα | 0.43 | T157^3.42^_Cα--V262^ICL3^_Cα | -0.31 |
| W226^5.31^_Cα--M265^ICL3^_Cα | 0.43 | V163^3.48^_Cα--V262^ICL3^_Cα | -0.31 |
| V285^6.40^--V288^6.43^ | 0.43 | G136^ECL1^_Cα--V262^ICL3^_Cα | -0.31 |
| M265^ICL3^_Cα--A304^6.59^_Cα | 0.43 | I142^3.27^_Cα--V262^ICL3^_Cα | -0.30 |
| R165^3.50^--Y252^5.58^ | -0.43 | E229^5.35^_Cα--V262^ICL3^_Cα | -0.30 |
| I238^5.44^_Cα--M265^ICL3^_Cα | 0.42 | T160^3.45^_Cα--V262^ICL3^_Cα | -0.30 |
| M265^ICL3^_Cα--V286^6.41^_Cα | 0.42 | L259^5.65^_Cα--V262^ICL3^_Cα | -0.30 |
| E229^5.35^_Cα--M265^ICL3^_Cα | 0.42 | T220^ECL2^_Cα--V262^ICL3^_Cα | -0.30 |
| M265^ICL3^_Cα--L331^7.48^_Cα | 0.42 | W133^ECL1^_Cα--V262^ICL3^_Cα | -0.30 |
| P244^5.50^_Cα--M265^ICL3^_Cα | 0.42 | Y252^5.58^--M281^6.36^ | -0.30 |
| M281^6.36^--L339^H8^ | 0.42 | M265^ICL3^_Cα--L283^6.38^_Cα | -0.30 |
| Q124^2.60^_Cα--M265^ICL3^_Cα | 0.42 | M265^ICL3^_Cα--I298^6.53^_Cα | -0.30 |
| N127^2.63^_Cα--M265^ICL3^_Cα | 0.42 | V262^ICL3^_Cα--V316^7.33^_Cα | -0.30 |
| M130^2.66^_Cα--M265^ICL3^_Cα | 0.42 | V202^4.60^_Cα--V262^ICL3^_Cα | -0.30 |
| L121^2.57^_Cα--M265^ICL3^_Cα | 0.42 | V262^ICL3^_Cα--V286^6.41^_Cα | -0.30 |
| I247^5.53^_Cα--M265^ICL3^_Cα | 0.41 | V262^ICL3^_Cα--E310^ECL3^_Cα | -0.30 |
| W133^ECL1^_Cα--M265^ICL3^_Cα | 0.41 | V169^3.54^_Cα--V262^ICL3^_Cα | -0.30 |
| L232^5.38^_Cα--M265^ICL3^_Cα | 0.41 | L139^3.24^_Cα--V262^ICL3^_Cα | -0.30 |
| C235^5.41^_Cα--M265^ICL3^_Cα | 0.41 | L232^5.38^_Cα--V262^ICL3^_Cα | -0.29 |
| M265^ICL3^_Cα--L283^6.38^_Cα | 0.41 | W226^5.31^_Cα--V262^ICL3^_Cα | -0.29 |
| S214^ECL2^_Cα--M265^ICL3^_Cα | 0.41 | M205^4.63^_Cα--V262^ICL3^_Cα | -0.29 |
| M151^3.36^_Cα--M265^ICL3^_Cα | 0.41 | S214^ECL2^_Cα--V262^ICL3^_Cα | -0.29 |
| C217^ECL2^_Cα--M265^ICL3^_Cα | 0.41 | M265^ICL3^_Cα--I301^6.56^_Cα | -0.29 |
| H223^ECL2^_Cα--M265^ICL3^_Cα | 0.41 | G253^5.59^_Cα--V262^ICL3^_Cα | -0.29 |
| T118^2.54^_Cα--M265^ICL3^_Cα | 0.41 | N127^2.63^_Cα--V262^ICL3^_Cα | -0.29 |
| V286^6.41^_Cα--F289^6.44^_Cα | -0.41 | L283^6.38^_Cα--C292^6.47^_Cα | 0.29 |
| Y148^3.33^_Cα--M265^ICL3^_Cα | 0.40 | I247^5.53^_Cα--V262^ICL3^_Cα | -0.29 |
| T208^ECL2^_Cα--M265^ICL3^_Cα | 0.40 | I238^5.44^_Cα--V262^ICL3^_Cα | -0.29 |
| G136^ECL1^_Cα--M265^ICL3^_Cα | 0.40 | M265^ICL3^_Cα--F313^7.30^_Cα | -0.29 |
| S154^3.39^_Cα--M265^ICL3^_Cα | 0.40 | V262^ICL3^_Cα--R280^6.35^_Cα | -0.29 |
| C79^1.43^_Cα--M265^ICL3^_Cα | 0.40 | M265^ICL3^_Cα--R280^6.35^_Cα | -0.29 |
| L139^3.24^_Cα--M265^ICL3^_Cα | 0.40 | M265^ICL3^_Cα--E310^ECL3^_Cα | -0.29 |
| T220^ECL2^_Cα--M265^ICL3^_Cα | 0.40 | Q124^2.60^_Cα--V262^ICL3^_Cα | -0.28 |
| Y166^3.51^_Cα--M265^ICL3^_Cα | 0.40 | M265^ICL3^_Cα--V286^6.41^_Cα | -0.28 |
| S145^3.30^_Cα--M265^ICL3^_Cα | 0.40 | M130^2.66^_Cα--V262^ICL3^_Cα | -0.28 |
| I142^3.27^_Cα--M265^ICL3^_Cα | 0.39 | M265^ICL3^_Cα--A304^6.59^_Cα | -0.28 |
| S76^1.40^_Cα--M265^ICL3^_Cα | 0.39 | M265^ICL3^_Cα--P295^6.50^_Cα | -0.28 |
| G82^1.46^_Cα--M265^ICL3^_Cα | 0.39 | C235^5.41^_Cα--V262^ICL3^_Cα | -0.28 |
| A115^2.51^_Cα--M265^ICL3^_Cα | 0.39 | F178^ICL2^_Cα--V262^ICL3^_Cα | -0.28 |
| M265^ICL3^_Cα--V334^7.51^_Cα | 0.39 | V262^ICL3^_Cα--C292^6.47^_Cα | -0.28 |
| R211^ECL2^_Cα--M265^ICL3^_Cα | 0.39 | V250^5.56^_Cα--V262^ICL3^_Cα | -0.28 |
| V163^3.48^_Cα--M265^ICL3^_Cα | 0.39 | V262^ICL3^_Cα--F289^6.44^_Cα | -0.28 |
| T157^3.42^_Cα--M265^ICL3^_Cα | 0.39 | M265^ICL3^_Cα--T307^ECL3^_Cα | -0.28 |
| V202^4.60^_Cα--M265^ICL3^_Cα | 0.39 | L259^5.65^_Cα--L283^6.38^_Cα | -0.28 |
| M205^4.63^_Cα--M265^ICL3^_Cα | 0.38 | V262^ICL3^_Cα--T307^ECL3^_Cα | -0.28 |
| V169^3.54^_Cα--M265^ICL3^_Cα | 0.38 | A175^ICL2^_Cα--V262^ICL3^_Cα | -0.28 |
| T160^3.45^_Cα--M265^ICL3^_Cα | 0.38 | R211^ECL2^_Cα--V262^ICL3^_Cα | -0.28 |
| L112^2.48^_Cα--M265^ICL3^_Cα | 0.38 | V262^ICL3^_Cα--H319^7.36^_Cα | -0.28 |
| R263^ICL3^--D272^6.27^ | 0.38 | P172^ICL2^_Cα--V262^ICL3^_Cα | -0.27 |
| M265^ICL3^_Cα--R280^6.35^_Cα | 0.38 | V262^ICL3^--L275^6.30^ | 0.27 |

**Table 8. Top 75 (~1%) structural descriptors of the slowest conformational degrees of freedom accessible to morphine-bound and TRV-130-bound MOR identified by tICA dimension 8. Structural descriptors involving correlated residue sidechains are highlighted in red. Commonalities, if any, between the morphine-bound MOR and TRV-130-bound MOR are highlighted in green.**

| **Morphine-bound MOR** | | **TRV-130-bound MOR** | |
| --- | --- | --- | --- |
| **Structural Descriptors** | **Correlation** | **Structural Descriptors** | **Correlation** |
| V236^5.42^--Morphine | -0.75 | F221^ECL2^--TRV-130 | 0.53 |
| H297^6.52^--Morphine | -0.74 | N328^7.45^--TRV-130 | -0.50 |
| N127^2.63^--Morphine | 0.74 | D114^2.50^--TRV-130 | -0.48 |
| W133^ECL1^--Morphine | 0.73 | E229^5.35^--TRV-130 | 0.46 |
| H319^7.36^--Morphine | 0.72 | L158^3.43^--TRV-130 | -0.46 |
| Y75^1.39^--Morphine | 0.71 | C235^5.41^--TRV-130 | 0.44 |
| C217^ECL2^--Morphine | 0.70 | L110^2.46^--TRV-130 | -0.42 |
| V300^6.55^--Morphine | -0.67 | W293^6.48^--Y326^7.43^ | 0.41 |
| C140^3.25^--Morphine | 0.65 | Y148^3.33^_Cα--V262^ICL3^_Cα | 0.41 |
| D216^ECL2^--Morphine | 0.62 | S196^4.54^_Cα--V262^ICL3^_Cα | 0.41 |
| M151^3.36^--Morphine | -0.61 | S145^3.30^_Cα--V262^ICL3^_Cα | 0.41 |
| V143^3.28^--Morphine | 0.59 | I142^3.27^_Cα--V262^ICL3^_Cα | 0.41 |
| F237^5.43^--Morphine | -0.53 | M151^3.36^_Cα--V262^ICL3^_Cα | 0.41 |
| N150^3.35^--Morphine | -0.53 | S154^3.39^--W293^6.48^ | 0.41 |
| K233^5.39^--Morphine | -0.51 | Y166^3.51^_Cα--V262^ICL3^_Cα | 0.41 |
| L121^2.57^--Morphine | 0.50 | I193^4.51^_Cα--V262^ICL3^_Cα | 0.40 |
| T218^ECL2^--Morphine | 0.44 | C190^4.48^_Cα--V262^ICL3^_Cα | 0.40 |
| L232^5.38^--Morphine | -0.40 | T160^3.45^_Cα--V262^ICL3^_Cα | 0.40 |
| F152^3.37^--A240^5.46^ | -0.37 | V163^3.48^_Cα--V262^ICL3^_Cα | 0.40 |
| V262^ICL3^_Cα--M265^ICL3^_Cα | 0.37 | L139^3.24^_Cα--V262^ICL3^_Cα | 0.40 |
| Y148^3.33^--Morphine | -0.36 | V187^4.45^_Cα--V262^ICL3^_Cα | 0.40 |
| S268^ICL3^_Cα--K271^6.26^_Cα | 0.34 | G199^4.57^_Cα--V262^ICL3^_Cα | 0.40 |
| F152^3.37^--V236^5.42^ | -0.34 | V262^ICL3^_Cα--P295^6.50^_Cα | 0.40 |
| S125^2.61^--Morphine | 0.32 | T157^3.42^_Cα--V262^ICL3^_Cα | 0.40 |
| V169^3.54^_Cα--R277^6.32^_Cα | -0.31 | V169^3.54^_Cα--V262^ICL3^_Cα | 0.40 |
| S268^ICL3^--K271^6.26^ | -0.31 | V202^4.60^_Cα--V262^ICL3^_Cα | 0.39 |
| T120^2.56^--Morphine | 0.31 | S154^3.39^_Cα--V262^ICL3^_Cα | 0.39 |
| C190^4.48^--F156^3.41^ | 0.31 | I234^5.40^--TRV-130 | 0.39 |
| M265^ICL3^_Cα--N274^6.29^_Cα | 0.30 | I247^5.53^_Cα--V262^ICL3^_Cα | 0.38 |
| P172^ICL2^_Cα--N274^6.29^_Cα | -0.30 | L259^5.65^--V262^ICL3^ | -0.38 |
| P172^ICL2^_Cα--K271^6.26^_Cα | -0.30 | P244^5.50^_Cα--V262^ICL3^_Cα | 0.38 |
| Y166^3.51^_Cα--R277^6.32^_Cα | -0.30 | L259^5.65^_Cα--V262^ICL3^_Cα | 0.38 |
| A175^ICL2^_Cα--N274^6.29^_Cα | -0.30 | A184^4.42^_Cα--V262^ICL3^_Cα | 0.38 |
| S196^4.54^_Cα--L259^5.65^_Cα | 0.30 | G136^ECL1^_Cα--V262^ICL3^_Cα | 0.38 |
| G199^4.57^_Cα--L259^5.65^_Cα | 0.30 | T118^2.54^--TRV-130 | -0.38 |
| V202^4.60^_Cα--L259^5.65^_Cα | 0.30 | A175^ICL2^_Cα--V262^ICL3^_Cα | 0.37 |
| F156^3.41^--I198^4.56^ | -0.30 | F178^ICL2^_Cα--V262^ICL3^_Cα | 0.37 |
| V169^3.54^_Cα--N274^6.29^_Cα | -0.30 | K100^ICL1^_Cα--V262^ICL3^_Cα | 0.37 |
| K269^ICL3^--K271^6.26^ | 0.30 | M205^4.63^_Cα--V262^ICL3^_Cα | 0.37 |
| V169^3.54^_Cα--K271^6.26^_Cα | -0.29 | R258^5.64^--V262^ICL3^ | -0.37 |
| M265^ICL3^_Cα--K271^6.26^_Cα | 0.29 | T208^ECL2^_Cα--V262^ICL3^_Cα | 0.37 |
| F178^ICL2^_Cα--N274^6.29^_Cα | -0.29 | T218^ECL2^--TRV-130 | 0.37 |
| P172^ICL2^_Cα--R277^6.32^_Cα | -0.29 | Y106^2.42^_Cα--V262^ICL3^_Cα | 0.37 |
| A184^4.42^_Cα--L259^5.65^_Cα | 0.28 | W293^6.48^--N332^7.49^ | 0.37 |
| M205^4.63^_Cα--L259^5.65^_Cα | 0.28 | W293^6.48^--S329^7.46^ | 0.37 |
| A175^ICL2^_Cα--K271^6.26^_Cα | -0.28 | I256^5.62^_Cα--V262^ICL3^_Cα | 0.37 |
| G199^4.57^_Cα--V262^ICL3^_Cα | 0.28 | N109^2.45^_Cα--V262^ICL3^_Cα | 0.36 |
| V94^1.58^_Cα--V262^ICL3^_Cα | 0.28 | V262^ICL3^_Cα--C292^6.47^_Cα | 0.36 |
| A175^ICL2^_Cα--R277^6.32^_Cα | -0.28 | P172^ICL2^_Cα--V262^ICL3^_Cα | 0.36 |
| A184^4.42^_Cα--I256^5.62^_Cα | 0.28 | P181^4.39^_Cα--V262^ICL3^_Cα | 0.36 |
| S196^4.54^_Cα--I256^5.62^_Cα | 0.28 | A113^2.49^--TRV-130 | -0.36 |
| F123^2.59^--Morphine | 0.28 | T220^ECL2^_Cα--V262^ICL3^_Cα | 0.36 |
| I296^6.51^--Morphine | -0.28 | V262^ICL3^_Cα--F289^6.44^_Cα | 0.35 |
| P181^4.39^_Cα--L259^5.65^_Cα | 0.28 | C217^ECL2^_Cα--V262^ICL3^_Cα | 0.35 |
| G199^4.57^_Cα--I256^5.62^_Cα | 0.27 | M151^3.36^--W293^6.48^ | -0.35 |
| T160^3.45^_Cα--N274^6.29^_Cα | -0.27 | E229^5.35^_Cα--V262^ICL3^_Cα | 0.35 |
| S196^4.54^_Cα--V262^ICL3^_Cα | 0.27 | N230^5.36^--TRV-130 | 0.35 |
| F178^ICL2^_Cα--K271^6.26^_Cα | -0.27 | N332^7.49^--TRV-130 | -0.35 |
| M265^ICL3^_Cα--D340^H8^_Cα | 0.27 | L112^2.48^_Cα--V262^ICL3^_Cα | 0.35 |
| V262^ICL3^_Cα--D340^H8^_Cα | 0.27 | F241^5.47^_Cα--V262^ICL3^_Cα | 0.35 |
| V163^3.48^_Cα--N274^6.29^_Cα | -0.27 | V262^ICL3^_Cα--I298^6.53^_Cα | 0.35 |
| V163^3.48^_Cα--R277^6.32^_Cα | -0.27 | L232^5.38^_Cα--V262^ICL3^_Cα | 0.34 |
| P181^4.39^_Cα--I256^5.62^_Cα | 0.27 | T103^2.39^_Cα--V262^ICL3^_Cα | 0.34 |
| V202^4.60^_Cα--I256^5.62^_Cα | 0.27 | V250^5.56^_Cα--V262^ICL3^_Cα | 0.34 |
| V202^4.60^_Cα--V262^ICL3^_Cα | 0.27 | V262^ICL3^_Cα--V316^7.33^_Cα | 0.34 |
| Y166^3.51^_Cα--N274^6.29^_Cα | -0.27 | W133^ECL1^_Cα--V262^ICL3^_Cα | 0.34 |
| T97^ICL1^_Cα--V262^ICL3^_Cα | 0.27 | V262^ICL3^_Cα--I301^6.56^_Cα | 0.34 |
| M205^4.63^_Cα--V262^ICL3^_Cα | 0.27 | V262^ICL3^_Cα--V286^6.41^_Cα | 0.34 |
| A184^4.42^_Cα--V262^ICL3^_Cα | 0.26 | W226^5.31^_Cα--V262^ICL3^_Cα | 0.34 |
| T103^2.39^_Cα--V262^ICL3^_Cα | 0.26 | S329^7.46^--TRV-130 | -0.33 |
| V163^3.48^_Cα--K271^6.26^_Cα | -0.26 | V262^ICL3^_Cα--A304^6.59^_Cα | 0.33 |
| Y166^3.51^_Cα--K271^6.26^_Cα | -0.26 | V262^ICL3^_Cα--R277^6.32^_Cα | 0.33 |
| L121^2.57^_Cα--Y148^3.33^_Cα | 0.26 | V262^ICL3^_Cα--H319^7.36^_Cα | 0.33 |
| S145^3.30^_Cα--L259^5.65^_Cα | 0.26 | C235^5.41^_Cα--V262^ICL3^_Cα | 0.33 |
| M265^ICL3^_Cα--R277^6.32^_Cα | 0.26 | G253^5.59^_Cα--V262^ICL3^_Cα | 0.33 |

**Table 9. Top 75 (~1%) structural descriptors of the slowest conformational degrees of freedom accessible to morphine-bound and TRV-130-bound MOR identified by tICA dimension 9. Structural descriptors involving correlated residue sidechains are highlighted in red. Commonalities, if any, between the morphine-bound MOR and TRV-130-bound MOR are highlighted in green.**

| **Morphine-bound MOR** | | **TRV-130-bound MOR** | |
| --- | --- | --- | --- |
| **Structural Descriptors** | **Correlation** | **Structural Descriptors** | **Correlation** |
| Y91^1.55^--Y96^ICL1^ | -0.51 | L110^2.46^--Y336^7.53^ | 0.56 |
| V236^5.42^--Morphine | 0.43 | S261^5.67^--A264^ICL3^ | -0.45 |
| W133^ECL1^--Morphine | -0.43 | K260^5.66^--A264^ICL3^ | -0.43 |
| N127^2.63^--Morphine | -0.43 | V262^ICL3^_Cα--M265^ICL3^_Cα | 0.41 |
| H297^6.52^--Morphine | 0.43 | I93^1.57^--F343^H8^ | 0.30 |
| H319^7.36^--Morphine | -0.43 | S196^4.54^_Cα--D340^H8^_Cα | -0.27 |
| Y75^1.39^--Morphine | -0.41 | K260^5.66^--L275^6.30^ | -0.26 |
| A337^7.54^_Cα--E349^H8^_Cα | 0.40 | I193^4.51^_Cα--D340^H8^_Cα | -0.26 |
| C217^ECL2^--Morphine | -0.39 | S196^4.54^_Cα--F343^H8^_Cα | -0.26 |
| C140^3.25^--Morphine | -0.39 | N109^2.45^_Cα--D340^H8^_Cα | -0.25 |
| M205^4.63^_Cα--C346^H8^_Cα | 0.38 | L112^2.48^_Cα--D340^H8^_Cα | -0.25 |
| A337^7.54^_Cα--C346^H8^_Cα | 0.38 | Y106^2.42^_Cα--D340^H8^_Cα | -0.25 |
| C217^ECL2^_Cα--C346^H8^_Cα | 0.38 | A264^ICL3^--D272^6.27^ | 0.24 |
| T208^ECL2^_Cα--C346^H8^_Cα | 0.38 | I193^4.51^_Cα--F343^H8^_Cα | -0.24 |
| Y148^3.33^_Cα--E349^H8^_Cα | 0.37 | I107^2.43^--F343^H8^ | 0.24 |
| M205^4.63^_Cα--E349^H8^_Cα | 0.37 | I142^3.27^_Cα--D340^H8^_Cα | -0.23 |
| S145^3.30^_Cα--C346^H8^_Cα | 0.37 | C190^4.48^_Cα--D340^H8^_Cα | -0.23 |
| D216^ECL2^--Morphine | -0.37 | A184^4.42^_Cα--D340^H8^_Cα | -0.23 |
| V202^4.60^_Cα--C346^H8^_Cα | 0.36 | V286^6.41^--N332^7.49^ | -0.23 |
| L112^2.48^_Cα--C346^H8^_Cα | 0.36 | L139^3.24^_Cα--D340^H8^_Cα | -0.23 |
| Y148^3.33^_Cα--C346^H8^_Cα | 0.36 | V89^1.53^--F343^H8^ | 0.22 |
| V202^4.60^_Cα--E349^H8^_Cα | 0.36 | V187^4.45^_Cα--D340^H8^_Cα | -0.22 |
| A304^6.59^_Cα--C346^H8^_Cα | 0.36 | G199^4.57^_Cα--D340^H8^_Cα | -0.22 |
| A115^2.51^_Cα--C346^H8^_Cα | 0.36 | I256^5.62^--L275^6.30^ | -0.22 |
| A337^7.54^_Cα--I352^H8^_Cα | 0.36 | F347^H8^--C351^H8^ | 0.21 |
| A304^6.59^_Cα--E349^H8^_Cα | 0.35 | S145^3.30^_Cα--D340^H8^_Cα | -0.21 |
| T118^2.54^_Cα--C346^H8^_Cα | 0.35 | P181^4.39^_Cα--D340^H8^_Cα | -0.21 |
| V300^6.55^--Morphine | 0.35 | P181^4.39^_Cα--F343^H8^_Cα | -0.21 |
| M151^3.36^--Morphine | 0.35 | G199^4.57^_Cα--F343^H8^_Cα | -0.21 |
| S145^3.30^_Cα--E349^H8^_Cα | 0.35 | T103^2.39^_Cα--D340^H8^_Cα | -0.20 |
| P172^ICL2^_Cα--N274^6.29^_Cα | -0.35 | I256^5.62^--I278^6.33^ | -0.20 |
| V94^1.58^_Cα--C346^H8^_Cα | 0.35 | A115^2.51^_Cα--D340^H8^_Cα | -0.20 |
| M151^3.36^_Cα--E349^H8^_Cα | 0.35 | M205^4.63^_Cα--F343^H8^_Cα | -0.20 |
| C217^ECL2^_Cα--E349^H8^_Cα | 0.35 | I142^3.27^_Cα--F343^H8^_Cα | -0.20 |
| I142^3.27^_Cα--C346^H8^_Cα | 0.35 | C190^4.48^_Cα--F343^H8^_Cα | -0.20 |
| I301^6.56^_Cα--E349^H8^_Cα | 0.35 | S196^4.54^_Cα--L331^7.48^_Cα | -0.20 |
| A175^ICL2^_Cα--N274^6.29^_Cα | -0.34 | Y149^3.34^--S196^4.54^ | 0.20 |
| T220^ECL2^_Cα--E349^H8^_Cα | 0.34 | A184^4.42^_Cα--F343^H8^_Cα | -0.20 |
| I301^6.56^_Cα--C346^H8^_Cα | 0.34 | M205^4.63^_Cα--E349^H8^_Cα | -0.20 |
| A115^2.51^_Cα--E349^H8^_Cα | 0.34 | S145^3.30^_Cα--F343^H8^_Cα | -0.20 |
| T220^ECL2^_Cα--C346^H8^_Cα | 0.34 | G136^ECL1^_Cα--D340^H8^_Cα | -0.20 |
| T208^ECL2^_Cα--E349^H8^_Cα | 0.34 | V202^4.60^_Cα--D340^H8^_Cα | -0.20 |
| L112^2.48^_Cα--E349^H8^_Cα | 0.33 | S196^4.54^_Cα--N328^7.45^_Cα | -0.20 |
| M151^3.36^_Cα--C346^H8^_Cα | 0.33 | N86^1.50^--N332^7.49^ | 0.19 |
| T118^2.54^_Cα--E349^H8^_Cα | 0.33 | V202^4.60^_Cα--F343^H8^_Cα | -0.19 |
| P172^ICL2^_Cα--K271^6.26^_Cα | -0.33 | M205^4.63^_Cα--D340^H8^_Cα | -0.19 |
| L121^2.57^_Cα--C346^H8^_Cα | 0.33 | S196^4.54^_Cα--C346^H8^_Cα | -0.19 |
| W133^ECL1^_Cα--C346^H8^_Cα | 0.33 | V334^7.51^--V89^1.53^ | -0.19 |
| S268^ICL3^_Cα--K271^6.26^_Cα | 0.33 | I193^4.51^_Cα--N328^7.45^_Cα | -0.19 |
| I322^7.39^_Cα--C346^H8^_Cα | 0.33 | I193^4.51^_Cα--L331^7.48^_Cα | -0.19 |
| F178^ICL2^_Cα--N274^6.29^_Cα | -0.33 | S145^3.30^_Cα--E349^H8^_Cα | -0.19 |
| N328^7.45^_Cα--C346^H8^_Cα | 0.33 | Y91^1.55^_Cα--D340^H8^_Cα | -0.19 |
| P172^ICL2^_Cα--R277^6.32^_Cα | -0.32 | S196^4.54^_Cα--E349^H8^_Cα | -0.19 |
| L139^3.24^_Cα--C346^H8^_Cα | 0.32 | N109^2.45^_Cα--F343^H8^_Cα | -0.18 |
| Q124^2.60^_Cα--C346^H8^_Cα | 0.32 | L139^3.24^_Cα--F343^H8^_Cα | -0.18 |
| G136^ECL1^_Cα--C346^H8^_Cα | 0.32 | V202^4.60^_Cα--E349^H8^_Cα | -0.18 |
| A175^ICL2^_Cα--K271^6.26^_Cα | -0.32 | T208^ECL2^_Cα--D340^H8^_Cα | -0.18 |
| G199^4.57^_Cα--C346^H8^_Cα | 0.31 | V187^4.45^_Cα--F343^H8^_Cα | -0.18 |
| S214^ECL2^_Cα--C346^H8^_Cα | 0.31 | I142^3.27^_Cα--E349^H8^_Cα | -0.18 |
| Y91^1.55^_Cα--C346^H8^_Cα | 0.31 | F156^3.41^--S195^4.53^ | 0.18 |
| I142^3.27^_Cα--E349^H8^_Cα | 0.31 | L112^2.48^_Cα--F343^H8^_Cα | -0.18 |
| I298^6.53^_Cα--C346^H8^_Cα | 0.31 | A264^ICL3^--S266^ICL3^ | 0.18 |
| N127^2.63^_Cα--C346^H8^_Cα | 0.31 | M151^3.36^_Cα--S196^4.54^_Cα | -0.18 |
| I298^6.53^_Cα--E349^H8^_Cα | 0.31 | T208^ECL2^_Cα--F343^H8^_Cα | -0.18 |
| E229^5.35^_Cα--E349^H8^_Cα | 0.31 | M205^4.63^_Cα--C346^H8^_Cα | -0.17 |
| Y91^1.55^_Cα--E349^H8^_Cα | 0.31 | G199^4.57^_Cα--E349^H8^_Cα | -0.17 |
| L121^2.57^_Cα--E349^H8^_Cα | 0.30 | D340^H8^_Cα--F343^H8^_Cα | -0.17 |
| A304^6.59^_Cα--F343^H8^_Cα | 0.30 | V169^3.54^--L275^6.30^ | 0.17 |
| N328^7.45^_Cα--E349^H8^_Cα | 0.30 | Y106^2.42^_Cα--F343^H8^_Cα | -0.17 |
| G199^4.57^_Cα--E349^H8^_Cα | 0.30 | I142^3.27^_Cα--C346^H8^_Cα | -0.17 |
| G325^7.42^_Cα--C346^H8^_Cα | 0.30 | L259^5.65^_Cα--M265^ICL3^_Cα | 0.17 |
| S268^ICL3^--K271^6.26^ | -0.30 | S196^4.54^_Cα--G325^7.42^_Cα | -0.17 |
| F178^ICL2^_Cα--K271^6.26^_Cα | -0.30 | V89^1.53^--A337^7.54^ | 0.17 |
| A113^2.49^--Morphine | 0.30 | V169^3.54^_Cα--L283^6.38^_Cα | -0.16 |
| I301^6.56^_Cα--F343^H8^_Cα | 0.30 | N104^2.40^--F343^H8^ | 0.16 |

**Table 10. Top 75 (~1%) structural descriptors of the slowest conformational degrees of freedom accessible to morphine-bound and TRV-130-bound MOR identified by tICA dimension 10. Structural descriptors involving correlated residue sidechains are highlighted in red. Commonalities, if any, between the morphine-bound MOR and TRV-130-bound MOR are highlighted in green.**

| **Morphine-bound MOR** | | **TRV-130-bound MOR** | |
| --- | --- | --- | --- |
| **Structural Descriptors** | **Correlation** | **Structural Descriptors** | **Correlation** |
| N342^H8^--C346^H8^ | 0.40 | E229^5.35^_Cα--A337^7.54^_Cα | -0.41 |
| N342^H8^--R345^H8^ | 0.40 | F241^5.47^_Cα--A337^7.54^_Cα | -0.40 |
| T70^1.34^_Cα--V169^3.54^_Cα | 0.40 | L232^5.38^_Cα--A337^7.54^_Cα | -0.40 |
| T70^1.34^_Cα--Y166^3.51^_Cα | 0.39 | I238^5.44^_Cα--A337^7.54^_Cα | -0.38 |
| A73^1.37^_Cα--V169^3.54^_Cα | 0.38 | W226^5.31^_Cα--A337^7.54^_Cα | -0.38 |
| A73^1.37^_Cα--Y166^3.51^_Cα | 0.38 | C235^5.41^_Cα--A337^7.54^_Cα | -0.37 |
| T67^1.31^_Cα--V169^3.54^_Cα | 0.38 | H223^ECL2^_Cα--A337^7.54^_Cα | -0.37 |
| E341^H8^--R345^H8^ | 0.38 | M151^3.36^_Cα--A337^7.54^_Cα | -0.36 |
| T70^1.34^_Cα--T157^3.42^_Cα | 0.38 | V202^4.60^_Cα--A337^7.54^_Cα | -0.36 |
| A73^1.37^_Cα--T157^3.42^_Cα | 0.37 | G199^4.57^_Cα--A337^7.54^_Cα | -0.36 |
| T70^1.34^_Cα--V163^3.48^_Cα | 0.37 | S154^3.39^_Cα--A337^7.54^_Cα | -0.36 |
| T67^1.31^_Cα--Y166^3.51^_Cα | 0.37 | T220^ECL2^_Cα--A337^7.54^_Cα | -0.36 |
| T70^1.34^_Cα--T160^3.45^_Cα | 0.37 | Y148^3.33^_Cα--A337^7.54^_Cα | -0.35 |
| T70^1.34^_Cα--D340^H8^_Cα | 0.37 | S196^4.54^_Cα--A337^7.54^_Cα | -0.35 |
| A73^1.37^_Cα--V163^3.48^_Cα | 0.36 | V334^7.51^_Cα--A337^7.54^_Cα | 0.35 |
| T67^1.31^_Cα--D340^H8^_Cα | 0.36 | M205^4.63^_Cα--A337^7.54^_Cα | -0.35 |
| A73^1.37^_Cα--T160^3.45^_Cα | 0.36 | I193^4.51^_Cα--A337^7.54^_Cα | -0.34 |
| S145^3.30^_Cα--A337^7.54^_Cα | 0.35 | P244^5.50^_Cα--A337^7.54^_Cα | -0.33 |
| T70^1.34^_Cα--S154^3.39^_Cα | 0.35 | R280^6.35^_Cα--D340^H8^_Cα | 0.33 |
| F338^7.55^--K344^H8^ | 0.35 | C190^4.48^_Cα--A337^7.54^_Cα | -0.32 |
| T70^1.34^_Cα--A175^ICL2^_Cα | 0.35 | Y106^2.42^_Cα--A337^7.54^_Cα | -0.32 |
| S64^Nterm^_Cα--D340^H8^_Cα | 0.35 | N109^2.45^_Cα--A337^7.54^_Cα | -0.31 |
| A73^1.37^_Cα--D340^H8^_Cα | 0.35 | T157^3.42^_Cα--A337^7.54^_Cα | -0.31 |
| T67^1.31^_Cα--I256^5.62^_Cα | 0.35 | V163^3.48^_Cα--A337^7.54^_Cα | -0.30 |
| T118^2.54^_Cα--D340^H8^_Cα | 0.35 | V94^1.58^_Cα--R277^6.32^_Cα | 0.30 |
| T67^1.31^_Cα--L259^5.65^_Cα | 0.34 | T160^3.45^_Cα--A337^7.54^_Cα | -0.30 |
| T67^1.31^_Cα--V163^3.48^_Cα | 0.34 | A111^2.47^--A337^7.54^ | 0.30 |
| L121^2.57^_Cα--D340^H8^_Cα | 0.34 | S145^3.30^_Cα--A337^7.54^_Cα | -0.30 |
| I142^3.27^_Cα--A337^7.54^_Cα | 0.34 | A337^7.54^_Cα--C346^H8^_Cα | 0.29 |
| T70^1.34^_Cα--I256^5.62^_Cα | 0.34 | I278^6.33^--L339^H8^ | 0.29 |
| M205^4.63^_Cα--A337^7.54^_Cα | 0.34 | I247^5.53^_Cα--A337^7.54^_Cα | -0.29 |
| V202^4.60^_Cα--A337^7.54^_Cα | 0.34 | V187^4.45^_Cα--A337^7.54^_Cα | -0.29 |
| A73^1.37^_Cα--S154^3.39^_Cα | 0.34 | T97^ICL1^_Cα--R277^6.32^_Cα | 0.28 |
| L112^2.48^_Cα--A337^7.54^_Cα | 0.34 | I142^3.27^_Cα--A337^7.54^_Cα | -0.28 |
| T67^1.31^_Cα--A175^ICL2^_Cα | 0.34 | T208^ECL2^_Cα--A337^7.54^_Cα | -0.28 |
| T208^ECL2^_Cα--A337^7.54^_Cα | 0.34 | R277^6.32^_Cα--D340^H8^_Cα | 0.28 |
| T67^1.31^_Cα--G253^5.59^_Cα | 0.34 | L112^2.48^_Cα--A337^7.54^_Cα | -0.28 |
| A115^2.51^_Cα--D340^H8^_Cα | 0.34 | Y166^3.51^_Cα--A337^7.54^_Cα | -0.27 |
| V334^7.51^_Cα--D340^H8^_Cα | 0.34 | V94^1.58^_Cα--R280^6.35^_Cα | 0.27 |
| C217^ECL2^_Cα--A337^7.54^_Cα | 0.34 | V250^5.56^_Cα--A337^7.54^_Cα | -0.27 |
| F289^6.44^--Y336^7.53^ | -0.34 | Y336^7.53^--D340^H8^ | -0.27 |
| A115^2.51^_Cα--A337^7.54^_Cα | 0.34 | T97^ICL1^_Cα--R280^6.35^_Cα | 0.27 |
| T70^1.34^_Cα--P172^ICL2^_Cα | 0.34 | V288^6.43^--W293^6.48^ | -0.27 |
| S154^3.39^_Cα--A337^7.54^_Cα | 0.34 | R280^6.35^_Cα--F343^H8^_Cα | 0.26 |
| T67^1.31^_Cα--T157^3.42^_Cα | 0.34 | A337^7.54^_Cα--F343^H8^_Cα | 0.26 |
| T70^1.34^_Cα--L259^5.65^_Cα | 0.34 | K100^ICL1^_Cα--R277^6.32^_Cα | 0.26 |
| Q124^2.60^_Cα--D340^H8^_Cα | 0.34 | T103^2.39^_Cα--A337^7.54^_Cα | -0.26 |
| T67^1.31^_Cα--T160^3.45^_Cα | 0.33 | V282^6.37^--L339^H8^ | 0.26 |
| W133^ECL1^_Cα--D340^H8^_Cα | 0.33 | F289^6.44^--C292^6.47^ | 0.25 |
| T70^1.34^_Cα--V250^5.56^_Cα | 0.33 | C217^ECL2^_Cα--A337^7.54^_Cα | -0.25 |
| T70^1.34^_Cα--G253^5.59^_Cα | 0.33 | A337^7.54^_Cα--E349^H8^_Cα | 0.25 |
| S64^Nterm^_Cα--V169^3.54^_Cα | 0.33 | A184^4.42^_Cα--A337^7.54^_Cα | -0.25 |
| T67^1.31^_Cα--V250^5.56^_Cα | 0.33 | L283^6.38^_Cα--D340^H8^_Cα | 0.24 |
| T118^2.54^_Cα--A337^7.54^_Cα | 0.33 | I193^4.51^_Cα--D340^H8^_Cα | -0.24 |
| T67^1.31^_Cα--P172^ICL2^_Cα | 0.33 | K100^ICL1^_Cα--P295^6.50^_Cα | 0.24 |
| Y148^3.33^_Cα--A337^7.54^_Cα | 0.33 | Y91^1.55^_Cα--R280^6.35^_Cα | 0.24 |
| G199^4.57^_Cα--A337^7.54^_Cα | 0.33 | F289^6.44^_Cα--A337^7.54^_Cα | -0.24 |
| N127^2.63^_Cα--D340^H8^_Cα | 0.33 | L139^3.24^_Cα--A337^7.54^_Cα | -0.24 |
| L139^3.24^_Cα--A337^7.54^_Cα | 0.33 | T103^2.39^_Cα--R277^6.32^_Cα | 0.24 |
| N109^2.45^_Cα--A337^7.54^_Cα | 0.33 | V94^1.58^_Cα--N274^6.29^_Cα | 0.24 |
| S64^Nterm^_Cα--I256^5.62^_Cα | 0.33 | Y91^1.55^_Cα--R277^6.32^_Cα | 0.23 |
| G82^1.46^_Cα--D340^H8^_Cα | 0.33 | S196^4.54^_Cα--D340^H8^_Cα | -0.23 |
| G136^ECL1^_Cα--A337^7.54^_Cα | 0.33 | N109^2.45^_Cα--R277^6.32^_Cα | 0.23 |
| I193^4.51^_Cα--A337^7.54^_Cα | 0.33 | F241^5.47^_Cα--V334^7.51^_Cα | -0.23 |
| T70^1.34^_Cα--M151^3.36^_Cα | 0.33 | M281^6.36^--D340^H8^ | -0.23 |
| S64^Nterm^_Cα--L259^5.65^_Cα | 0.33 | Y106^2.42^_Cα--R277^6.32^_Cα | 0.23 |
| T67^1.31^_Cα--N274^6.29^_Cα | 0.32 | K100^ICL1^_Cα--I298^6.53^_Cα | 0.23 |
| A337^7.54^--K344^H8^ | 0.32 | C190^4.48^_Cα--D340^H8^_Cα | -0.23 |
| C190^4.48^_Cα--A337^7.54^_Cα | 0.32 | V169^3.54^_Cα--A337^7.54^_Cα | -0.23 |
| G136^ECL1^_Cα--D340^H8^_Cα | 0.32 | R277^6.32^_Cα--F343^H8^_Cα | 0.23 |
| T70^1.34^_Cα--N274^6.29^_Cα | 0.32 | T97^ICL1^_Cα--N274^6.29^_Cα | 0.23 |
| S76^1.40^_Cα--D340^H8^_Cα | 0.32 | G199^4.57^_Cα--L331^7.48^_Cα | -0.22 |
| L121^2.57^_Cα--A337^7.54^_Cα | 0.32 | K100^ICL1^_Cα--R280^6.35^_Cα | 0.22 |
| T70^1.34^_Cα--F178^ICL2^_Cα | 0.32 | V316^7.33^_Cα--F343^H8^_Cα | 0.22 |
| I107^2.43^--A337^7.54^ | -0.32 | H319^7.36^_Cα--F343^H8^_Cα | 0.22 |

**Table 11. Top transmitters identified in the morphine-bound and TRV-130-bound MOR systems.** Only residues with a hub score larger than 0.6 are reported. Transmitters in one ligand-bound MOR system that act as receivers in the other are highlighted in red. Transmitters in one ligand-bound MOR system that act as connectors in the other are highlighted in green. Transmitters that are common to the two-ligand bound MOR systems are highlighted in bold.

| **Morphine-bound MOR** | | **TRV-130-bound MOR** | |
| --- | --- | --- | --- |
| **Residue** | **Hub value** | **Residue** | **Hub value** |
| **R165^3.50^** | **1.00** | **Y336^7.53^** | **0.96** |
| **V282^6.37^** | **0.90** | **R165^3.50^** | **0.95** |
| **L339^H8^** | **0.89** | L110^2.46^ | 0.93 |
| **Y336^7.53^** | **0.87** | V285^6.40^ | 0.90 |
| **I278^6.33^** | **0.86** | V286^6.41^ | 0.89 |
| **M161^3.46^** | **0.86** | **M161^3.46^** | **0.87** |
| Y252^5.58^ | 0.85 | **V282^6.37^** | **0.85** |
| D340^H8^ | 0.84 | L283^6.38^ | 0.84 |
| **M281^6.36^** | **0.82** | **I278^6.33^** | **0.83** |
| **F338^7.55^** | **0.77** | S162^3.47^ | 0.82 |
| **L275^6.30^** | **0.76** | **M281^6.36^** | **0.81** |
| **L259^5.65^** | **0.76** | **I256^5.62^** | **0.79** |
| **I256^5.62^** | **0.74** | **L275^6.30^** | **0.79** |
| **K271^6.26^** | **0.68** | **L339^H8^** | **0.79** |
| V262^ICL3^ | 0.67 | **L259^5.65^** | **0.77** |
| **R258^5.64^** | **0.67** | T279^6.34^ | 0.74 |
| **M264^ICL3^** | **0.66** | V92^1.56^ | 0.73 |
| R263^ICL3^ | 0.65 | K260^5.66^ | 0.73 |
| S268^ICL3^ | 0.63 | V169^3.54^ | 0.69 |
| **F84^1.48^** | **0.61** | F156^3.41^ | 0.69 |
| **L88^1.52^** | **0.61** | **F338^7.55^** | **0.69** |
|  |  | Y166^3.51^ | 0.68 |
|  |  | **F84^1.48^** | **0.68** |
|  |  | **L88^1.52^** | **0.68** |
|  |  | **R258^5.64^** | **0.65** |
|  |  | R276^6.31^ | 0.65 |
|  |  | A168^3.53^ | 0.64 |
|  |  | Y96^ICL1^ | 0.63 |
|  |  | **K271^6.26^** | **0.63** |
|  |  | **M264^ICL3^** | **0.63** |
|  |  | P172^ICL2^ | 0.63 |
|  |  | S317^7.34^ | 0.62 |
|  |  | N86^1.50^ | 0.62 |
|  |  | T315^7.32^ | 0.61 |
|  |  | G253^5.59^ | 0.61 |
|  |  | V89^1.53^ | 0.60 |

**Table 12. Top receivers identified in the morphine-bound and TRV-130-bound MOR systems. Only residues with an authority score larger than 0.6 are reported. Receivers in one ligand-bound MOR system that act as transmitters in the other are highlighted in red. Receivers in one ligand-bound MOR system that act as connectors in the other are highlighted in green. Receivers that are common to the two-ligand bound MOR systems are highlighted in bold.**

| **Morphine-bound MOR** | | **TRV-130-bound MOR** | |
| --- | --- | --- | --- |
| **Residue** | **Authority value** | **Residue** | **Authority value** |
| N328^7.45^ | 1.00 | L121^2.57^ | 1.00 |
| D164^3.49^ | 0.92 | D147^3.32^ | 0.92 |
| Y106^2.42^ | 0.83 | **A184^4.42^** | **0.90** |
| D114^2.50^ | 0.78 | V143^3.28^ | 0.87 |
| **R345^H8^** | **0.78** | T118^2.54^ | 0.87 |
| **I93^1.57^** | **0.77** | V78^1.42^ | 0.87 |
| C346^H8^ | 0.76 | Q124^2.60^ | 0.87 |
| **K344^H8^** | **0.75** | A323^7.40^ | 0.85 |
| W293^6.48^ | 0.75 | T160^3.45^ | 0.85 |
| **F108^2.44^** | **0.75** | A102^2.38^ | 0.84 |
| **G267^ICL3^** | **0.74** | **Y75^1.39^** | **0.83** |
| **V163^3.48^** | **0.74** | **F108^2.44^** | **0.82** |
| K269^ICL3^ | 0.74 | **V163^3.48^** | **0.81** |
| R179^ICL2^ | 0.74 | T294^6.49^ | 0.81 |
| M151^3.36^ | 0.74 | I242^5.48^ | 0.81 |
| R276^6.31^ | 0.74 | T153^3.38^ | 0.81 |
| **L257^5.63^** | **0.73** | C330^7.47^ | 0.80 |
| V288^6.43^ | 0.73 | **L257^5.63^** | **0.77** |
| G325^7.42^ | 0.72 | **P244^5.50^** | **0.77** |
| C292^6.47^ | 0.71 | I105^2.41^ | 0.76 |
| **T97^ICL1^** | **0.71** | I298^6.53^ | 0.75 |
| **A184^4.42^** | **0.70** | N191^4.49^ | 0.75 |
| **M99^ICL1^** | **0.69** | **T97^ICL1^** | **0.74** |
| S154^3.39^ | 0.68 | V187^4.45^ | 0.74 |
| R273^6.28^ | 0.67 | **T249^5.55^** | **0.74** |
| I155^3.40^ | 0.67 | I247^5.53^ | 0.73 |
| F241^5.47^ | 0.67 | **A113^2.49^** | **0.72** |
| Y166^3.51^ | 0.66 | Y149^3.34^ | 0.72 |
| V92^1.56^ | 0.66 | T120^2.56^ | 0.72 |
| N342^H8^ | 0.66 | **M99^ICL1^** | **0.71** |
| N150^3.35^ | 0.66 | G199^4.57^ | 0.71 |
| D272^6.27^ | 0.66 | T180^ICL2^ | 0.71 |
| I290^6.45^ | 0.66 | **I93^1.57^** | **0.70** |
| F347^H8^ | 0.65 | K303^6.58^ | 0.70 |
| A287^6.42^ | 0.65 | K233^5.39^ | 0.70 |
| C170^3.55^ | 0.65 | C235^5.41^ | 0.70 |
| A168^3.53^ | 0.64 | V300^6.55^ | 0.70 |
| P172^ICL2^ | 0.64 | L139^3.24^ | 0.70 |
| E270^6.25^ | 0.64 | **R345^H8^** | **0.69** |
| A240^5.46^ | 0.63 | I308^ECL3^ | 0.69 |
| **S261^5.67^** | **0.62** | M243^5.49^ | 0.69 |
| T101^2.37^ | 0.62 | **S261^5.67^** | **0.69** |
| A117^2.53^ | 0.62 | L331^7.48^ | 0.68 |
| G253^5.59^ | 0.61 | P201^4.59^ | 0.68 |
| **A113^2.49^** | **0.61** | I238^5.44^ | 0.68 |
| **T249^5.55^** | **0.61** | F237^5.43^ | 0.67 |
| S329^7.46^ | 0.60 | P309^ECL3^ | 0.67 |
| **P244^5.50^** | **0.60** | K98^ICL1^ | 0.67 |
| **Y75^1.39^** | **0.60** | A115^2.51^ | 0.67 |
|  |  | V81^1.45^ | 0.67 |
|  |  | C79^1.43^ | 0.66 |
|  |  | P295^6.50^ | 0.66 |
|  |  | V236^5.42^ | 0.65 |
|  |  | C251^5.57^ | 0.65 |
|  |  | **K344^H8^** | **0.65** |
|  |  | W192^4.50^ | 0.64 |
|  |  | I301^6.56^ | 0.64 |
|  |  | N274^6.29^ | 0.64 |
|  |  | S196^4.54^ | 0.64 |
|  |  | I146^3.31^ | 0.63 |
|  |  | S145^3.30^ | 0.63 |
|  |  | I193^4.51^ | 0.63 |
|  |  | S119^2.55^ | 0.63 |
|  |  | S266^ICL3^ | 0.62 |
|  |  | F178^ICL2^ | 0.62 |
|  |  | N183^4.41^ | 0.62 |
|  |  | A304^6.59^ | 0.62 |
|  |  | K185^4.43^ | 0.62 |
|  |  | P333^7.50^ | 0.62 |
|  |  | I186^4.44^ | 0.62 |
|  |  | S195^4.53^ | 0.61 |
|  |  | V202^4.60^ | 0.61 |
|  |  | N127^2.63^ | 0.61 |
|  |  | **G267^ICL3^** | **0.61** |
|  |  | M90^1.54^ | 0.61 |
|  |  | L112^2.48^ | 0.61 |
|  |  | G82^1.46^ | 0.61 |
|  |  | T307^ECL3^ | 0.60 |

**Table 13. Top connectors (i.e. residues that act as both transmitters and receivers) identified in the morphine-bound and TRV-130-bound MOR systems. Residues are ordered by residue numbers, and those that are unique to a particular system are shown in black. Connectors in one ligand-bound MOR system that act as transmitters in the other are highlighted in green. Connectors in one ligand-bound MOR system that act as receivers in the other are highlighted in red. Connectors that are common in the two-ligand bound MOR systems are highlighted in bold.**

| **Morphine-bound MOR** | | | **TRV-130-bound MOR** | | |
| --- | --- | --- | --- | --- | --- |
| **Residue** | **Authority value** | **Hub value** | **Residue** | **Authority value** | **Hub value** |
|  |  |  | T101^2.37^ | 0.70 | 0.65 |
| **T103^2.39^** | **0.65** | **0.82** | **T103^2.39^** | **0.62** | **0.86** |
| **N104^2.40^** | **0.72** | **0.72** | **N104^2.40^** | **0.64** | **0.77** |
|  |  |  | Y106^2.42^ | 0.94 | 0.65 |
| **I107^2.43^** | **0.73** | **0.85** | **I107^2.43^** | **0.61** | **0.84** |
|  |  |  | N109^2.45^ | 0.71 | 0.60 |
| L110^2.46^ | 0.74 | 0.67 |  |  |  |
|  |  |  | D114^2.50^ | 0.60 | 0.92 |
|  |  |  | A117^2.53^ | 0.91 | 0.60 |
|  |  |  | Y148^3.33^ | 0.86 | 0.61 |
|  |  |  | N150^3.35^ | 0.72 | 0.86 |
|  |  |  | M151^3.36^ | 0.75 | 0.82 |
|  |  |  | F152^3.37^ | 0.65 | 0.77 |
|  |  |  | S154^3.39^ | 0.73 | 0.92 |
|  |  |  | I155^3.40^ | 0.80 | 0.94 |
| **T157^3.42^** | **0.85** | **0.64** | **T157^3.42^** | **0.68** | **0.85** |
| **L158^3.43^** | **0.63** | **0.73** | **L158^3.43^** | **0.65** | **0.94** |
| **C159^3.44^** | **0.79** | **0.65** | **C159^3.44^** | **0.74** | **0.87** |
| S162^3.47^ | 0.61 | 0.78 |  |  |  |
|  |  |  | D164^3.49^ | 0.77 | 0.64 |
| V169^3.54^ | 0.69 | 0.64 |  |  |  |
|  |  |  | R179^ICL2^ | 0.65 | 0.76 |
|  |  |  | N188^4.46^ | 0.76 | 0.60 |
|  |  |  | A240^5.46^ | 0.71 | 0.80 |
|  |  |  | F241^5.47^ | 0.73 | 0.72 |
|  |  |  | V245^5.51^ | 0.81 | 0.70 |
| **I248^5.54^** | **0.67** | **0.72** | **I248^5.54^** | **0.70** | **0.81** |
|  |  |  | Y252^5.58^ | 0.61 | 0.81 |
| **M255^5.61^** | **0.70** | **0.68** | **M255^5.61^** | **0.67** | **0.75** |
| K260^5.66^ | 0.66 | 0.63 |  |  |  |
| N274^6.29^ | 0.65 | 0.67 |  |  |  |
| **R277^6.32^** | **0.81** | **0.63** | **R277^6.32^** | **0.65** | **0.62** |
| T279^6.34^ | 0.71 | 0.67 |  |  |  |
| **R280^6.35^** | **0.68** | **0.82** | **R280^6.35^** | **0.63** | **0.80** |
| L283^6.38^ | 0.67 | 0.83 |  |  |  |
| **V284^6.39^** | **0.84** | **0.60** | **V284^6.39^** | **0.79** | **0.66** |
| V285^6.40^ | 0.70 | 0.82 |  |  |  |
| V286^6.41^ | 0.67 | 0.79 |  |  |  |
|  |  |  | A287^6.42^ | 0.75 | 0.69 |
|  |  |  | V288^6.43^ | 0.77 | 0.66 |
| **F289^6.44^** | **0.67** | **0.61** | **F289^6.44^** | **0.62** | **0.98** |
|  |  |  | I290^6.45^ | 0.81 | 0.75 |
|  |  |  | V291^6.46^ | 0.72 | 0.69 |
|  |  |  | C292^6.47^ | 0.77 | 0.72 |
|  |  |  | W293^6.48^ | 0.65 | 1.00 |
|  |  |  | I296^6.51^ | 0.76 | 0.62 |
|  |  |  | H297^6.52^ | 0.77 | 0.67 |
|  |  |  | W318^7.35^ | 0.61 | 0.66 |
|  |  |  | H319^7.36^ | 0.66 | 0.61 |
|  |  |  | C321^7.38^ | 0.71 | 0.63 |
|  |  |  | I322^7.39^ | 0.83 | 0.66 |
|  |  |  | L324^7.41^ | 0.76 | 0.63 |
|  |  |  | G325^7.42^ | 0.77 | 0.85 |
|  |  |  | Y326^7.43^ | 0.86 | 0.82 |
|  |  |  | T327^7.44^ | 0.84 | 0.70 |
|  |  |  | N328^7.45^ | 0.84 | 0.90 |
|  |  |  | S329^7.46^ | 0.74 | 0.80 |
| **N332^7.49^** | **0.87** | **0.64** | **N332^7.49^** | **0.64** | **0.98** |
| **V334^7.51^** | **0.63** | **0.60** | **V334^7.51^** | **0.60** | **0.62** |
| **L335^7.52^** | **0.70** | **0.74** | **L335^7.52^** | **0.67** | **0.81** |
| **A337^7.54^** | **0.74** | **0.75** | **A337^7.54^** | **0.67** | **0.73** |
|  |  |  | D340^H8^ | 0.63 | 0.81 |
| **E341^H8^** | **0.74** | **0.72** | **E341^H8^** | **0.68** | **0.60** |
|  |  |  | N342^H8^ | 0.69 | 0.67 |
| **F343^H8^** | **0.79** | **0.65** | **F343^H8^** | **0.65** | **0.74** |
|  |  |  | C346^H8^ | 0.61 | 0.67 |

**Table 14.** **Most probable de****activation pathways from the active macrostate #11 to the inactive macrostate #12 of the morphine-bound MOR system, predicted using Transition path theory. Only pathways with more than 1% of flux are reported. Given the detail-balance constraint, activation proceeds, with the same fluxes, through pathways with macrostates in the reverse order.**

| **% Path** | **% of total** | **Path (macrostate ids)** |
| --- | --- | --- |
| 40.4 | 40.4 | 11 🡪 12 |
| 33.3 | 73.7 | 11 🡪 7 🡪 12 |
| 14.3 | 88.0 | 11 🡪 8 🡪 12 |
| 6.0 | 94.0 | 11 🡪 7 🡪 8 🡪 12 |
| 1.8 | 95.8 | 11 🡪 7 🡪 14 🡪 12 |
| 1.2 | 97.0 | 11 🡪 10 🡪 12 |

**Table 15. Most probable deactivation pathways from the active macrostate #9 to the inactive macrostate #12 of the TRV-130-bound MOR system, predicted using Transition path theory.** Only pathways with more than 1% of flux are reported. Given the detail-balance constraint, activation proceeds, with the same fluxes, through pathways with macrostates in the reverse order.

| **% Path** | **% of total** | **Path (macrostate ids)** |
| --- | --- | --- |
| 31.9 | 31.9 | 9 🡪 7 🡪 12 |
| 19.6 | 51.5 | 9 🡪 7 🡪 13 🡪 14 🡪 12 |
| 17.9 | 69.4 | 9 🡪 7 🡪 11 🡪 14 🡪 12 |
| 17.3 | 86.7 | 9 🡪 7 🡪 14 🡪 12 |
| 3.9 | 90.6 | 9 🡪 14 🡪 12 |
| 3.2 | 93.8 | 9 🡪 7 🡪 11 🡪13 🡪 14 🡪 12 |
| 1.3 | 95.1 | 9 🡪 7 🡪 3 🡪 11 🡪 12 |
